# Nuclear cGAS governs transcription to control lipid metabolism, tumor growth, and therapy response

**DOI:** 10.1172/JCI205579

**Published:** 2026-11-02

**Authors:** Yu Deng, Matthew J. Niederhuber, Zhichuan Zhu, Austin J. Hepperla, Le Yu, Quentin Hahn, Joshua Hartsell, Tooba Rashid, Piotr A. Mieczkowski, Hemant Kelkar, Jeremy M. Simon, Yanqiong Zhang, Alessandra Livraghi-Butrico, Meredith J. Zeller, Joseph G.C. Kennedy, Jazmin F. DePriest, Jiong Li, Yan Zhang, Xing Qiu, H. Ümit Kaniskan, Jian Jin, Corbin D. Jones, Laura E. Herring, Douglas H. Phanstiel, Xiaojing Liu, Jesse R. Raab, Brian D. Strahl, Pengda Liu

**Affiliations:** 1 Lineberger Comprehensive Cancer Center,; 2 Department of Biochemistry and Biophysics,; 3 Bioinformatics and Analytics Research Collaborative (BARC),; 4 Carolina Institute for Developmental Disabilities,; 5 University of North Carolina (UNC) Neuroscience Center, and; 6 Department of Genetics, University of North Carolina at Chapel Hill, Chapel Hill, North Carolina, USA.; 7 Department of Molecular and Structural Biochemistry, North Carolina State University, Raleigh, North Carolina, USA.; 8 Curriculum in Bioinformatics and Computational Biology, University of North Carolina at Chapel Hill, Chapel Hill, North Carolina, USA.; 9 Department of Data Science, Dana-Farber Cancer Institute Department of Biostatistics, Harvard T.H. Chan School of Public Health, Boston, Massachusetts, USA.; 10 Marsico Lung Institute/UNC Cystic Fibrosis Center, School of Medicine, University of North Carolina at Chapel Hill, Chapel Hill, North Carolina, USA.; 11 Department of Medicinal Chemistry, School of Pharmacy;; 12 Philips Institute for Oral and Craniofacial Molecular Biology, School of Dentistry; and; 13 Massey Comprehensive Cancer Center, Virginia Commonwealth University, Richmond, Virginia, USA.; 14 Mount Sinai Center for Therapeutics Discovery, Departments of Pharmacological Sciences, Oncological Sciences and Neuroscience, Tisch Cancer Institute, Icahn School of Medicine at Mount Sinai, New York, USA.; 15 Department of Biology;; 16 UNC Proteomics Core Facility, Department of Pharmacology;; 17 Thurston Arthritis Research Center; and; 18 Department of Cell Biology and Physiology, University of North Carolina at Chapel Hill, Chapel Hill, North Carolina, USA.

**Keywords:** Cell biology, Metabolism, Oncology, Epigenetics

## Abstract

Cyclic GMP-AMP synthase (cGAS) is a cytosolic DNA sensor critical for host defense against microbial infections. To prevent aberrant activation, nuclear cGAS is restrained by nucleosome tethering or by barrier-to-autointegration factor–mediated restriction of its access to DNA. However, its physiological roles beyond enzymatic suppression in the nucleus remain poorly understood. Here, we present a genome-wide map of cGAS chromatin localization in human cells and unexpectedly find its binding beyond nucleosome tethering, where cGAS is recruited to chromatin by AP-1 transcription factors via a potentially previously uncharacterized transactivation domain. Integrated *CUT&RUN*, RNA-seq, and metabolomics analyses reveal that cGAS transcriptionally regulates cholesterol homeostasis. Mechanistically, cGAS promotes *LGALS3* transcription, which inhibits ACAA2 activity to suppress fatty acid oxidation and acetyl-CoA production, thereby lowering intracellular cholesterol levels. Reduced cholesterol lowers plasma membrane phosphatidylinositol [3,4,5] triphosphate [PI(3,4,5)P_3_] levels and decreases mTOR complex 2 (mTORC2) activation. In contrast, cGAS depletion results in cholesterol accumulation and elevated PI(3,4,5)P_3_, driving mTORC2/Akt signaling, promoting xenografted tumor growth, and conferring resistance to DNA-damaging agents. In vivo, the elevated hepatic cholesterol levels observed in *cGas^–/–^* mice were largely reversed by inhibition of Akt but not STING, whereas cGAS deficiency had minimal effects on lung physiology. Collectively, our findings uncover an unexpected role for nuclear cGAS in orchestrating a transcriptional program that links lipid metabolism to cell proliferation, stress response, and cholesterol homeostasis in liver.

## Abstract 2

Nuclear cGAS is recruited by AP-1 transcription factors to regulate transcriptional programs controlling cholesterol homeostasis and cellular behavior.

## Introduction

Sensing aberrant cytosolic DNA is a critical host defense mechanism mediated primarily by cyclic GMP-AMP synthase (cGAS) in mammals (1, 2). Cytosolic DNA from foreign sources, such as viral or bacterial infection, or endogenous sources, such as damaged mitochondrial or genomic DNA, activates cGAS to produce the second messenger 2′3′-cGAMP, which binds and activates ER-localized stimulator of interferon genes (STING), inducing type I interferon transcription (3) and promoting adaptive immunity and clearance of infected or damaged cells (4). Dysregulated cGAS activation can cause autoimmune disease (5), whereas cGAS agonists show promise in enhancing antitumor immunity and therapeutic responses (6).

Although cGAS is best known as a cytosolic DNA sensor, it also localizes to the nucleus, where it associates with DNA double-strand breaks and suppresses homologous recombination through interaction with poly (ADP-ribose) polymerase 1 (PARP1) (7). Nuclear cGAS also localizes to replication forks, centromeres, and LINE repeats, where it may contribute to genome stability, though its functions at the latter sites remain poorly understood (8). Nuclear cGAS is tightly bound to the acidic patches of nucleosomal H2A/H2B, keeping it inactive (9). Additional mechanisms restrain its activation, including N-terminal phosphorylation by mitotic kinases (10), mTOR complex 2 (mTORC2) (11), or RSK2 (12); inhibition by the barrier-to-autointegration factor (BAF) (13); and SPSB3-mediated degradation of nucleosome-bound cGAS (14). Collectively, these mechanisms prevent inappropriate cGAS activation in the DNA-rich nucleus.

It may seem counterintuitive for cells to sequester a substantial fraction of cGAS in chromatin, particularly given the lack of evidence that nuclear cGAS is acutely mobilized to the cytoplasm upon DNA virus infection. We therefore hypothesized that chromatin-associated cGAS may have functions beyond immune sensing. Using *CUT&RUN* with cGAS mutants defective in DNA binding (KKEA) or nucleosome binding (R255E), we found that most chromatin-associated cGAS is neither tethered to nucleosomes nor dependent on DNA binding. Instead, cGAS is recruited to promoters by AP-1 transcription factors through a potentially previously unrecognized transactivation domain (TAD). Integrated *CUT&RUN* and RNA-seq analyses revealed that cGAS promotes galectin-3 (*LGALS3*) transcription, which suppresses cholesterol homeostasis by inhibiting acetyl-CoA acyltransferase 2 (ACAA2), a key enzyme in fatty acid β-oxidation. Consistent with this mechanism, metabolomic analyses showed altered cholesterol metabolism, leading to reduced mTORC2/Akt signaling, smaller cell size, impaired tumor growth, and increased sensitivity to apoptosis-inducing agents. Together, our findings identify a nucleosome-independent role for nuclear cGAS in AP-1–dependent transcription and cholesterol homeostasis, linking nuclear cGAS to mTORC2/Akt signaling, tumor growth, and apoptotic sensitivity.

## Results

### cGAS CUT&RUN reveals a nucleosome-independent genomic distribution distinct from its innate immune function.

Consistent with previous reports (7–9), cGAS localized to both the cytoplasm and nucleus across multiple human and mouse cell types (Supplemental Figure 1, A and B; supplemental material available online with this article; https://doi.org/10.1172/JCI205579DS1), with most nuclear cGAS associated with chromatin (Figure 1A and Supplemental Figure 1, C–G). We generated *cGAS^−/−^* MDA-MB-231 cells and reconstituted them with WT or DNA-binding–deficient KKEA-cGAS (Supplemental Figure 1H). Both proteins showed cytoplasmic and nuclear localization similar to endogenous cGAS (Supplemental Figure 1, I and J). Given the reported high-affinity binding of cGAS to nucleosomes (15–20), we asked whether chromatin-associated cGAS regulates 3D genome architecture. Micro-C analysis of empty vector–reconstituted (EV-reconstituted) and WT-cGAS–reconstituted cells revealed no appreciable differences in chromatin organization (Figure 1B and Supplemental Figure 1K), suggesting that cGAS does not substantially regulate 3D chromatin architecture.

Given that nuclear cGAS has functions beyond innate immunity, including inhibition of PARP1-mediated DNA repair (21), regulation of replication fork progression (7), and centromere binding (8), we asked whether chromatin-associated cGAS regulates transcription. *CUT&RUN* analysis of EV-, WT- and KKEA-cGAS-expressing MDA-MB-231 *cGAS^−/−^* cells revealed robust genome-wide cGAS binding. WT- and KKEA-cGAS showed highly overlapping peaks (Figure 1, C–E), indicating similar genomic localization independent of DNA binding. These peaks partially overlapped with H3K4me3, a promoter-associated mark (Figure 1E), which was further confirmed at individual loci (Supplemental Figure 1L). Thus, chromatin-associated cGAS localizes genome-wide independently of its DNA-binding activity.

Because cGAS binds the acidic patch of nucleosomes (15–20), we included the nucleosome-binding–deficient R255E-cGAS mutant in *CUT&RUN* analysis. To control for altered cGAS expression or localization following reconstitution, we also mapped endogenous cGAS in parental shScr cells. Approximately 50% of endogenous cGAS peaks were recapitulated by reconstituted WT-cGAS (Figure 1F and Supplemental Figure 1, M–O), validating these sites while highlighting potential reconstitution-associated false positives. We therefore classified peaks absent in R255E-cGAS as “nucleosomal” peaks. Remaining peaks were divided into “neo-gained” peaks, unique to R255E-cGAS, and “true-cGAS” peaks, shared by endogenous, WT-, and R255E-cGAS, representing non-nucleosomal cGAS binding sites.

In MDA-MB-231 cells, we identified 212,634 high-confidence cGAS *CUT&RUN* peaks at non-nucleosomal regions and 6,374 at nucleosomal regions (Figure 1F). Among these, 18,960 true-cGAS peaks represented high-confidence, non-nucleosomal cGAS-binding sites and were the focus of this study. Notably, nucleosome-binding–deficient R255E-cGAS exhibited more and stronger non-nucleosomal peaks than nucleosomal peaks (Figure 1G and Figure 2A), suggesting that nucleosome tethering not only maintains cGAS inactivity (9) but also may restrict its localization to non-nucleosomal chromatin regions.

Further analysis revealed distinct genomic distributions of high-confidence true-cGAS peaks. Promoters were preferentially enriched in true-cGAS peaks (16.9%) compared with nucleosomal (9.3%) and neo-gained (8.7%) peaks (Figure 2B), suggesting a potential role for cGAS in transcriptional regulation. Genome browser analysis confirmed cGAS occupancy across these categories, including overlapping endogenous, WT-, and R255E-cGAS peaks at the *LGALS3* promoter (Figure 2C). These findings suggest 3 potential chromatin-associated states of cGAS: nucleosome-tethered, nucleosome- and transcription factor–associated, and transcription factor/chromatin remodeler–associated (Figure 2D). These distinct states are explored below.

### Integrative CUT&RUN, RNA-seq, and nuclear cGAS TurboID analyses reveal that cGAS interacts with AP-1 transcription factors to regulate genes involved in cholesterol homeostasis.

To determine whether non-nucleosomal chromatin-associated cGAS regulates transcription, we performed RNA-seq in EV-, WT-cGAS–, and KKEA-cGAS–expressing MDA-MB-231 *cGAS^−/−^* cells (Figure 3A). Integrative analysis of cGAS *CUT&RUN* and RNA-seq identified cholesterol homeostasis as the top cGAS-regulated pathway, followed by fatty acid metabolism (Figure 3B and Supplemental Figure 2A). Among cholesterol-related genes, WT- and KKEA-cGAS increased *LGALS3* and *PPARG* transcription relative to EV (Figure 3C and Supplemental Figure 2B). However, high-confidence true-cGAS peaks were detected at the *LGALS3*, but not *PPARG*, locus (Figure 3D and Supplemental Figure 2C). WT-, KKEA-, and R255E-cGAS all occupied the *LGALS3* promoter (Figure 3E and Figure 2C), and R255E-cGAS promoted *LGALS3* transcription similarly to WT-cGAS (Figure 3F). Consistently, cGAS deletion reduced *LGALS3* expression in HeLa, A498, and BPH1 cells (Supplemental Figure 2, D–F). These findings identify *LGALS3* as a potential direct transcriptional target of cGAS.

To explore the recruitment of cGAS to non-nucleosomal genomic regions, we conducted transcription factor motif enrichment analysis. AME (22) and de novo STREME analyses revealed strong enrichment of AP-1 motifs (RTGASTCAY) within true-cGAS *CUT&RUN* peaks (Figure 4A and Supplemental Figure 2G). Notably, AP-1 motifs were enriched in true-cGAS but depleted in nucleosomal peaks, suggesting that nucleosome tethering may influence cGAS association with AP-1 factors. Notably, we observed a shift in transcription factor motifs from nucleosomal cGAS peaks to true-cGAS peaks (Figure 4A), suggesting that cGAS binding to AP-1 transcription factors is influenced by its tethering to H2A/H2B acidic patches in certain genomic regions. To identify nuclear cGAS-interacting proteins, we performed nuclear cGAS TurboID proteomics (Supplemental Figure 2, H–L), identifying 675 proteins enriched ≥2-fold over controls. Known cGAS partners, including PARP1 (7) and BAF (13), validated the approach. Among the top transcription factors were the AP-1 family members JunB and FosL1 (Figure 4B), consistent with the motif analysis.

We validated cGAS interactions with endogenous JunB and FosL1 in MDA-MB-231 cells (Figure 4, C and D), with similar interactions observed for exogenous cGAS and in THP1 cells (Supplemental Figure 2, M–O). JunB depletion reduced chromatin-associated cGAS (Figure 4E), while FosL1 degradation using the FosL1-PROTAC VZP1052 (23) produced a similar effect in MDA-MB-231 and THP1 cells (Supplemental Figure 2, P–S). Using a transcription factor targeting PROteolysis-TArgeting Chimera (TF-PROTAC) strategy (24, 25), we generated AP-1 TF-PROTACs by conjugating the canonical AP-1–binding sequence TGACTCA to von Hippel-Lindau (VHL) ligands. Several compounds (#7–11) reduced JunB/FosL1 levels and cGAS chromatin association (Figure 4F and Supplemental Figure 2, T and U). Depletion of JunB or FosL1 also reduced *LGALS3* transcription (Figure 4, G and H, and Supplemental Figure 2, V–Y). Together, these findings support AP-1–dependent recruitment of nuclear cGAS to non-nucleosomal chromatin, particularly through JunB and FosL1.

### cGAS contains TADs necessary for transcription factor binding.

To determine how nuclear cGAS promotes transcription through JunB and FosL1, we asked whether cGAS contains TADs, which mediate transcriptional activation (26). The 9aaTAD algorithm (27) predicted 5 putative TADs, with TAD2 identified as a high-confidence TAD containing 2 canonical Φ-Φ-x-x-Φ motifs (Supplemental Figure 3, A and B). Using an AP-1–mCherry reporter assay (28), we found that JunB depletion or FosL1 degradation by VZP1052 reduced AP-1 activity in control but not *cGAS^−/−^* cells, where cGAS re-expression restored this activity (Supplemental Figure 3, C–E). Deletion of individual TADs reduced AP-1 reporter activity and cGAS binding to JunB or FosL1 and impaired *LGALS3* transcription (Supplemental Figure 3, F–I). Together, these findings indicate that multiple cGAS TADs contribute to its interaction with AP-1 factors and transcriptional activity.

To minimize effects of protein conformational changes caused by TAD deletions, we focused on evolutionarily conserved Phe residues within these domains (Figure 5, A and B). Compared with F226A/F230A, the F491A/F503A and 4FA (F226A/F230A/F491A/F503A) mutants markedly reduced AP-1 reporter activity (Figure 5A) and impaired binding to JunB and FosL1 (Figure 5, C and D, and Supplemental Figure 3, J and K). However, unlike 4FA, the F491A/F503A mutant retained DNA-induced cGAS activation (Figure 5, E and F). Although F491A/F503A showed reduced chromatin localization, it retained nucleosome binding (Figure 5, G and H, and Supplemental Figure 3, L–N) but failed to efficiently promote *LGALS3* transcription (Figure 5, I–K). In contrast, DNA-binding–deficient KKEA and nucleosome-binding–deficient R255E cGAS retained JunB/FosL1 binding (Supplemental Figure 3O). Thus, we used F491A/F503A-cGAS as a key model to dissect the transcriptional function of cGAS independently of its canonical immune activity (Supplemental Figure 3P).

We next performed structural modeling and molecular dynamics (MD) simulations of the chromatin-bound cGAS/AP-1 complex to define their interaction interface. The complex remained stable throughout equilibration, with 4 conserved polar interaction networks and extensive hydrophobic contacts preserved. cGAS engaged AP-1 adjacent to its KKH DNA-binding domain, positioning the functionally critical F503 at the center of the interface (Supplemental Figure 4A). Specifically, cGAS-R499, K427, Y510/N514, and N513 formed persistent interactions with FosL1-E159, E152, E141, and JunB-K299, respectively (Supplemental Figure 4B). F503 also occupied a hydrophobic pocket within FosL1, where its aromatic ring packed against the aliphatic portion of FosL1-E152. These interactions provide a structural basis for the critical role of F503 in stabilizing cGAS/AP-1 binding, consistent with our biochemical findings.

### cGAS governs AP-1–dependent transcriptional programs.

Beyond *LGALS3*, we identified cGAS-bound genomic regions enriched for AP-1 motifs and occupied by endogenous, WT-, and R255E-cGAS (Supplemental Figure 5, A–H). RT-qPCR validated several targets and confirmed their dependence on cGAS and its TAD (Supplemental Figure 5, I and J). To define the broader cGAS/AP-1 transcriptional program, we performed RNA-seq in *cGAS^−/−^* MDA-MB-231 cells reconstituted with WT-, F491A/F503A-cGAS, or WT-cGAS with JunB depletion (WT-cGAS-shJunB) (Supplemental Figure 5K). We identified 725 genes downregulated by both F491A/F503A-cGAS and JunB depletion relative to WT-cGAS, with *LGALS3* among the top 2 affected genes (Supplemental Figure 5, L–N). Gene Ontology analysis linked these cGAS/AP-1 targets to cell-cell junction organization, proliferation, and other tumor-related processes (Supplemental Figure 5O).

### cGAS governs transcription by complexing with transcription modulators.

Nuclear cGAS TurboID identified, in addition to transcription factors (Figure 4B), multiple chromatin regulators, suggesting their involvement in cGAS/AP-1–dependent transcription. Chromatin fractionation and cGAS immunoprecipitation confirmed interactions with AP-1 factors as well as chromatin regulators SMARCA5 and SMARCB1 and epigenetic modifiers KMT2A and HDAC1 (Supplemental Figure 6A). Depletion of SMARCA5 or SMARCB1 reduced cGAS-dependent *LGALS3* transcription (Supplemental Figure 6, B–D), supporting their functional role in this pathway. Although the cGAS N-terminus mediates DNA binding (29, 30) and oligomerization (31), the isolated N-terminus failed to promote *LGALS3* transcription, whereas an N-terminal deletion mutant retained this activity (Supplemental Figure 6, E and F). Both constructs localized to the nucleus, but only the N-terminal deletion mutant efficiently associated with chromatin (Supplemental Figure 6, G and H).

Previous studies showed that nuclear cGAS tethering to nucleosomes is regulated by N-terminal phosphorylation by mitotic kinases (10) and mTORC2 (11). We therefore asked whether these pathways also regulate cGAS-dependent transcription. Nocodazole synchronization of HeLa cells followed by washout revealed increased cGAS and AP-1 recruitment to chromatin during late G1, coinciding with enhanced *LGALS3* transcription (Supplemental Figure 6, I–K). Conversely, mTORC2 inhibition with Torin 2 increased cytoplasmic cGAS and reduced *LGALS3* expression (Supplemental Figure 6, L and M), consistent with decreased nuclear cGAS activity.

DNA-damaging agents such as doxorubicin can promote cGAS nuclear translocation through B-lymphoid tyrosine kinase–dependent phosphorylation (21). Consistent with this, etoposide increased nuclear cGAS accumulation and *LGALS3* transcription in MDA-MB-231 cells (Supplemental Figure 6, N and O), likely through enhanced chromatin association. Because AP-1 activity is regulated by JNK-mediated phosphorylation (32), we tested whether JNK activity affects cGAS recruitment. JNK inhibition with SP600125 reduced cGAS–AP-1 association on chromatin (Supplemental Figure 6P), suggesting that JNK-dependent AP-1 activation promotes cGAS recruitment and transcriptional activity.

### Metabolomics reveals that the cGAS/LGALS3/ACAA2 axis regulates fatty acid oxidation, acetyl-CoA production, and cholesterol synthesis.

Our integrated cGAS *CUT&RUN* and RNA-seq analyses identified cholesterol homeostasis as the top cGAS-regulated pathway (Figure 3, A and B). Filippin III staining (33) showed that WT-cGAS re-expression reduced cellular cholesterol in MDA-MB-231 *cGAS^−/−^* cells, whereas LGALS3 depletion restored cholesterol accumulation (Figure 6A). Consistently, the transcriptionally inactive F491A/F503A-cGAS mutant failed to suppress cholesterol levels in MDA-MB-231, mouse embryonic fibroblast (MEF), BPH1, and THP1 cells (Figure 6, B and C, and Supplemental Figure 7, A–C), consistent with its impaired AP-1 binding and *LGALS3* transcription. In contrast, *STING* deficiency did not increase cholesterol in HeLa cells (Supplemental Figure 7, D and E), indicating that cGAS regulates cholesterol independently of canonical STING signaling. Consistent with these findings, *cGas^−/−^* C57BL/6 mouse livers showed increased cholesterol and reduced *Lgals3* expression (Figure 6, D and E), supporting a role for the cGAS/LGALS3 axis in suppressing hepatic cholesterol accumulation.

BioGRID analysis of cGAS-interacting proteins identified several metabolic enzymes involved in fatty acid and cholesterol metabolism (Figure 7A). Among these, ACAA2, which catalyzes the final step of mitochondrial fatty acid β-oxidation (34), partially colocalized with LGALS3 in MDA-MB-231 cells (Figure 7B) and directly interacted with LGALS3 in vitro (Figure 7, C and D). Importantly, LGALS3 inhibited ACAA2 enzymatic activity (Figure 7E and Supplemental Figure 7F). Consistent with this mechanism, WT-cGAS re-expression reduced acetyl-CoA levels in MDA-MB-231 *cGAS^−/−^* cells, an effect largely reversed by LGALS3 depletion (Figure 7F). Increasing ACAA2 activity elevated cholesterol, whereas ACAA2 inhibition with trimetazidine reduced cholesterol specifically in cGAS-deficient cells (Supplemental Figure 7, G–I). Together, these findings support a cGAS/LGALS3/ACAA2 axis that restrains cholesterol synthesis by limiting fatty acid β-oxidation and acetyl-CoA production.

Because ACAA2 generates acetyl-CoA, a precursor for cholesterol synthesis (34), we next examined whether cGAS regulates fatty acid oxidation. Metabolomics and ^13^C-palmitate tracing in parental, *cGAS^−/−^*, and *cGAS^−/−^* cells reconstituted with WT-cGAS or LGALS3 revealed that cGAS loss increased fatty acid oxidation, reflected by elevated ^13^C-labeled citrate and palmitoyl-carnitine (Figure 7G). *cGAS* deletion also increased glycolysis, energy metabolites, acyl-carnitines, and amino acids, changes largely reversed by WT-cGAS or LGALS3 re-expression (Figure 7H). Similar metabolic alterations occurred in HeLa but not *STING^−/−^* cells (Supplemental Figure 7, J and K), supporting a STING-independent role for cGAS in metabolic regulation. Additionally, acetyl-CoA serves as the starting substrate for cholesterol synthesis, with the rate-limiting step being the conversion of HMG-CoA to mevalonate by HMG-CoA reductase (HMGCR) (35). Inhibition of HMGCR with lovastatin reduced cholesterol in both WT and *cGAS^−/−^* cells (Figure 7I), implicating de novo cholesterol synthesis in the cholesterol accumulation caused by cGAS loss. Together, these findings define a cGAS/LGALS3/ACAA2 axis that restrains fatty acid β-oxidation, acetyl-CoA production, and downstream cholesterol synthesis.

### Cholesterol restrains phosphatase and tensin homolog on the plasma membrane to elevate phosphatidylinositol [3,4,5] triphosphate levels and subsequent mTORC2 activation.

Cholesterol has been reported to influence lipid raft composition (36), trafficking (37), PI3K activity (38), or other physiological function (39). Consistent with a role in mTOR regulation, WT-cGAS re-expression in *cGAS^−/−^* cells reduced plasma membrane phosphatidylinositol [3,4,5] triphosphate [PI(3,4,5)P_3_] levels, an effect largely reversed by LGALS3 depletion (Figure 8A and Supplemental Figure 8A). Conversely, LGALS3 re-expression reduced PI(3,4,5)P_3_ in *cGAS^−/−^* cells (Supplemental Figure 8B), linking cGAS/LGALS3 signaling to cholesterol-dependent PI345P_3_ regulation. *cGAS* loss also reduced membrane localization of phosphatase and tensin homolog (PTEN) (Figure 8B), a key PI(3,4,5)P_3_ phosphatase (40). Previously, we reported PI(3,4,5)P_3_ is critical for mTORC2 plasma membrane localization and activation (41). Elevated PI(3,4,5)P_3_ in *cGAS^−/−^* cells was accompanied by increased membrane localization of mTORC2 components Rictor and Sin1 (Figure 8C and Supplemental Figure 8C). Together, these findings suggest that cGAS suppresses mTORC2 activation through the cGAS/LGALS3/cholesterol/PI(3,4,5)P_3_ axis.

We next examined mTORC2 activity and found that *cGAS* deletion increased Akt-pS473, an mTORC2 substrate, in MDA-MB-231 cells, which was reversed by WT-cGAS reconstitution (Figure 8D and Supplemental Figure 8D). Re-expression of LGALS3 similarly suppressed Akt-pS473 induced by *cGAS* deletion (Figure 8E), whereas LGALS3 depletion or ACAA2 expression largely reversed the suppression mediated by WT-cGAS (Figure 8, F and G). Consistent with cGAS regulating mTORC2 independently of its innate immune function, KKEA- and R255E-cGAS, but not F491A/F503A-cGAS, suppressed Akt-pS473 (Figure 8, H and I, and Supplemental Figure 8, E and F). Cholesterol addition enhanced Akt-pS473, whereas lovastatin suppressed this effect (Figure 8, J and K, and Supplemental Figure 8, E–I). Collectively, these findings suggest that the cGAS/LGALS3/ACAA2 axis negatively regulates cholesterol levels to suppress mTORC2 activity (Figure 8L).

### Inhibition of Akt, but not STING, reverses cholesterol accumulation in cGAS-deficient mouse livers.

To further establish the role of mTORC2/Akt signaling in cGAS-mediated cholesterol homeostasis, we treated *cGas^–/–^* C57BL/6 mice with either Akt inhibitor capivasertib or STING inhibitor H-151. Capivasertib, but not H-151, reduced hepatic cholesterol levels in *cGas^–/–^* mice (Figure 9A), without affecting body weight (Supplemental Figure 9A), supporting a role for mTORC2/Akt signaling in regulating cholesterol independently of STING.

Because hepatic cholesterol accumulation and dysregulation are associated with pulmonary inflammation, fibrosis, and impaired surfactant homeostasis (42), and a 2024 preprint (43) reported that aged male *cGas^–/–^* mice develop spontaneous pro-inflammatory phenotypes in both the lung and liver, we asked whether cGAS deficiency causes pulmonary abnormalities in young adult mice. Histological analysis of 4-month-old *cGas^–/–^* mice and *Wt* littermates of both sexes revealed no overt differences in airway or alveolar architecture (Figure 9B). As in the liver, *Lgals3* expression was markedly reduced in *cGas^–/–^* lungs and was not restored by capivasertib or H-151 (Figure 9C), supporting mTORC2/Akt as a downstream pathway of the cGAS/LGALS3 axis. Bronchoalveolar lavage (BAL) analysis showed that macrophages predominated in both genotypes, with no significant differences in macrophage, neutrophil, or lymphocyte numbers (Figure 9, D–G). Lung macrophages from *cGas^–/–^* mice also lacked morphological features of activation or polarization (Supplemental Figure 9B). Body weight and lung wet/dry ratios were comparable between genotypes (Supplemental Figure 9, C–F). Thus, *cGas^–/–^* mice do not exhibit overt pulmonary inflammation or pathology under basal conditions at 4 months of age, despite reduced *Lgals3* expression.

However, under the stress of CO_2_ euthanasia, *cGas^–/–^* lungs appeared visibly enlarged, whereas the liver, heart, kidney, spleen, and overall body weight were unchanged (Supplemental Figure 10, A–K). Compared with *Wt* lungs, *cGas^–/–^* lungs exhibited reduced *Lgals3* expression, increased cholesterol levels, and elevated expression of inflammatory/fibrosis-associated genes, including *B2m*, *Cxcl9*, and *Ifitm3* (Supplemental Figure 10, L–P). These findings suggest that the cGAS/LGALS3/cholesterol axis may influence stress-induced pulmonary responses. However, CO_2_ euthanasia can itself induce hypoxia, hypercapnia, acidosis, and rapid changes in lung morphology, inflammation, gene expression, metabolism, and immune phenotypes (44–47). Thus, these findings should be interpreted cautiously, and further studies using euthanasia methods that minimize such artifacts are needed to determine whether cGAS deficiency promotes pulmonary pathology under stress or other challenge conditions.

### cGAS/LGALS3/mTORC2 signaling controls cell size and tumor growth.

The mTORC2/Akt pathway promotes cell proliferation and survival (48–50). We therefore examined whether the cGAS/LGALS3/ACAA2/mTORC2 axis regulates cell behavior. *cGAS* deletion increased mTORC2/Akt activation and cell size, which was phenocopied by LGALS3 depletion (Figure 10A and Figure 8F). This effect was independent of canonical cGAS/STING signaling, as STING depletion did not alter cell size (Figure 10, B and C, and Supplemental Figure 11, A–E). Re-expression of WT-, R255E-, or KKEA-cGAS suppressed anchorage-independent growth of *cGAS*-deficient MDA-MB-231 cells, accompanied by reduced cell size (Figure 10, D and E). In contrast, the AP-1–binding–deficient F491A/F503A-cGAS mutant failed to suppress soft agar growth (Figure 10, F and G). Consistent with LGALS3 being a major cGAS transcriptional target, LGALS3 re-expression similarly suppressed anchorage-independent growth (Figure 10, H and I).

To further define the role of the cGAS/LGALS3/ACAA2/acetyl-CoA/cholesterol/mTORC2 axis in tumor growth, we performed xenograft studies in nude mice. Whereas STING deletion had minimal effects, *cGAS* deletion markedly accelerated tumor growth in both HeLa and MDA-MB-231 xenografts (Figure 11, A–C, and Supplemental Figure 11, F–J), accompanied by increased mTORC2/Akt activation (Supplemental Figure 11, K–M). LGALS3 re-expression largely reversed the enhanced tumor growth and lipid accumulation caused by *cGAS* deletion (Figure 11, D–H, and Supplemental Figure 11, N and O), supporting LGALS3 as a key downstream effector of cGAS-mediated tumor suppression and lipid homeostasis. Consistently, unlike WT-cGAS, the transcriptionally defective F491A/F503A-cGAS mutant failed to suppress tumor growth in *cGAS*-deficient MDA-MB-231 xenografts (Figure 11, I–L, and Supplemental Figure 11, P and Q). Finally, reduced LGALS3 expression was associated with poorer survival in ovarian, lung, and gastric cancers (Supplemental Figure 11, R–T), further supporting a tumor-suppressive role for the cGAS/LGALS3 axis.

### cGAS/LGALS3/mTORC2 signaling regulates tumor therapeutic response.

mTORC2/Akt hyperactivation not only promotes tumor growth but also enhances apoptosis resistance (50), resists therapy (51), and drives angiogenic and metastatic processes (52). We therefore asked whether the cGAS/LGALS3/ACAA2/mTORC2 axis regulates therapeutic response. We started from chemotherapy because it is the first-line treatment for various cancer types (53, 54). In MDA-MB-231 and HeLa cells, *cGAS* deletion conferred resistance to doxorubicin and etoposide, which was reversed by WT-cGAS re-expression (Figure 12A and Supplemental Figure 12, A–E). LGALS3 re-expression similarly restored drug sensitivity, whereas the transcriptionally defective F491A/F503A-cGAS mutant did not (Figure 12, B–E, and Supplemental Figure 12, F and G). Consistent with elevated cholesterol and mTORC2/Akt activity in *cGAS*-deficient cells, *cGAS* deletion also conferred resistance to cholesterol-lowering agents, including lovastatin, atorvastatin, and triheptanoin, as well as the mTOR inhibitor Torin 2 and Akt inhibitor GSK2110183 (Figure 12, F–J). WT-cGAS, but not F491A/F503A-cGAS, largely restored drug sensitivity, which depended on LGALS3 expression (Figure 12, K–M). Long-term 3D soft agar assays further confirmed the requirement for cGAS transcriptional activity in responses to doxorubicin and lovastatin (Figure 13, A–D). Together, these findings identify the LGALS3/cholesterol/mTORC2 axis as a key downstream effector of cGAS that regulates responses to chemotherapy, cholesterol-lowering agents, and mTOR/Akt inhibition (Supplemental Figure 12H).

## Discussion

In this study, we uncover a potentially previously unrecognized role for nuclear cGAS in transcriptional regulation through AP-1 transcription factors. cGAS *CUT&RUN*, mutant analyses, and nuclear cGAS TurboID identified JunB and FosL1 as key recruiters of cGAS to chromatin, with transcription factor–recruited cGAS representing the main transcriptionally active pool. An evolutionarily conserved TAD, including F491 and F503, mediates cGAS binding to JunB/FosL1, while the F491A/F503A mutant retains DNA-induced innate immune and nucleosome-binding activities, demonstrating that cGAS transcriptional and canonical immune functions are separable. Integrated cGAS *CUT&RUN* and RNA-seq identified lipid metabolism, particularly cholesterol and fatty acid metabolism, as major cGAS-regulated programs, with LGALS3 emerging as a key target activated by AP-1–recruited cGAS. LGALS3 suppresses ACAA2-dependent fatty acid β-oxidation, limiting acetyl-CoA availability and cholesterol synthesis. Accordingly, cGAS loss increased cholesterol accumulation in cells and *cGas^−/−^* mouse livers, which was reversed by WT-cGAS or LGALS3 but not the transcriptionally defective F491A/F503A mutant. Elevated cholesterol in cGAS-deficient cells increased PI(3,4,5)P_3_ and activated mTORC2/Akt, thereby promoting tumor growth and resistance to DNA-damaging agents. These phenotypes were largely reversed by WT-cGAS or LGALS3 but not F491A/F503A-cGAS, establishing the cGAS/LGALS3 axis as a key link between nuclear cGAS, lipid metabolism, mTORC2/Akt signaling, tumor progression, and therapeutic response.

Our findings expand the growing body of evidence for noncanonical functions of cGAS beyond innate immune signaling. Previous studies have implicated cGAS in mitochondrial dynamics and ferroptosis (55), PI3K signaling (56), DNA repair through PARP1 (7), and replication fork stability (8). We now identify AP-1–dependent transcriptional regulation as an additional nuclear function of cGAS. The F491A/F503A mutant provides a useful tool for selectively disrupting this transcriptional activity while preserving DNA-induced immune signaling. Further studies are needed to define the physiological signals that regulate cGAS transcriptional activity.

cGAS has previously been linked to metabolism primarily through canonical STING-dependent signaling via interferon-stimulated genes and senescence-associated secretory phenotypes (57), but emerging evidence supports STING-independent metabolic functions. For example, cGAS was reported to regulate metabolic reprogramming in colon cancer by maintaining NDUFA4L2 expression (58) and to promote β cell proliferation and mass through C/EBP-β independently of STING (59). Our study establishes a direct transcriptional mechanism whereby nuclear cGAS regulates metabolism through LGALS3/ACAA2. Metabolomic analyses further revealed effects on glycolysis, though whether these changes are mediated through the cGAS/LGALS3 axis remains unclear. Notably, lipid accumulation occurred in *cGAS^−/−^* but not *STING^−/−^* xenografts, further supporting a STING-independent role for cGAS in lipid metabolism. How tumor cells handle and redistribute accumulated cholesterol and whether tumor-associated lipid storage influences tumor or immune cell function (60) remain important questions.

LGALS3 is a multifunctional protein involved in viral infectivity and tumor immune regulation (61–63). Our findings raise the possibility that nuclear cGAS-mediated *LGALS3* transcription may represent a common mechanism linking viral permissiveness and tumor immune evasion. This hypothesis will require validation in appropriate infection and immunocompetent tumor models.

Several questions remain. First, although AP-1 recruits cGAS to promoters, how chromatin-modifying enzymes and other transcriptional regulators cooperate with cGAS remains unknown. Second, cGAS also negatively regulates a subset of genes identified by *CUT&RUN*, and the mechanisms and physiological significance of this repression warrant investigation. Third, substantial cGAS occupancy occurs in intergenic regions, raising the possibility that cGAS regulates noncoding transcription. Finally, R255E-cGAS, originally designed as a nucleosome-binding–deficient mutant, displayed enhanced and altered transcription factor occupancy, suggesting potential gain-of-function activity and a distinct transcriptional program. Defining the molecular basis and biological consequences of this activity will further clarify the diverse functions of nuclear cGAS.

## Methods

### Sex as a biological variable

Only female mice were used in the xenograft studies to maintain consistency and reproducibility. Because the MDA-MB-231 breast cancer and HeLa cervical cancer cell lines were used to establish xenograft tumors, and both cancers predominantly occur in females, female mice were selected for these studies.

### Cell culture

MDA-MB-231, HEK293T, A498, and MEF cells were cultured in DMEM containing 10% FBS, whereas BPH1 and THP1 cells were cultured in RPMI-1640 with 10% FBS. Unless otherwise indicated, media were supplemented with 100 U/mL penicillin and 100 μg/mL streptomycin. MDA-MB-231 and HEK293T cells were purchased from ATCC; A498, MEF, BPH1, and THP1 cells were provided by the laboratories of William Kim (UNC-Chapel Hill), Nan Yan (UT Southwestern, Dallas, Texas, USA), Haojie Huang (Mayo Clinic, Rochester, Minnesota, USA), and Blossom Damania (UNC-Chapel Hill), respectively. Cell transfection and lentiviral packaging/infection were performed as previously described (64, 65). Infected cells were selected with blasticidin (5 μg/mL), puromycin (1 μg/mL), or hygromycin (200 μg/mL), as appropriate. Key reagents are listed in Supplemental Table 1.

### Plasmids

HA-cGAS-WT, F226A/F230A, and F491A/F503A were cloned into pCDNA3.0, while pLenti-HA-cGAS, KKEA, R255E, F226A/F230A, and F491A/F503A were cloned into pLenti-HA-blasticidin. pLenti-cGAS-Flag-miniTurbo was cloned into pLenti-GFP-blasticidin. HA-cGAS-ΔTAD1–TAD5, Flag-FosL1, Flag-STING, HA-LGALS3, and HA-ACAA2 were cloned into pCDNA3.0, and GST-ACAA2 was cloned into pGEX-6P-1. All constructs were generated by overlap PCR or site-directed mutagenesis. PCS2-Flag-JunB (Addgene plasmid 29687) and pSIRV-AP-1-mCherry (Addgene plasmid 118095) were purchased from Addgene. Cloning and mutagenesis primers are listed in Supplemental Table 2.

### Immunoblotting

Cells were lysed in EBC buffer (50 mM Tris-HCl, pH 7.5; 120 mM NaCl; 0.5% NP-40) supplemented with protease and phosphatase inhibitor cocktails. Protein concentrations were determined using the Bio-Rad protein assay reagent and a NanoDrop OneC (Thermo Fisher Scientific), as previously described (66, 67). Equal amounts of whole-cell lysates were separated by SDS-PAGE and immunoblotted with the indicated antibodies. For glutathione S-transferase pulldown and immunoprecipitation assays, 1 mg of lysate was incubated with the indicated beads for 3 to 4 hours at 4°C. Immunocomplexes were washed 3 times with NETN buffer (20 mM Tris-HCl, pH 8.0; 100 mM NaCl; 1 mM EDTA; 0.5% NP-40), followed by SDS-PAGE and immunoblotting. Antibodies are listed in Supplemental Table 1.

### Micro-C

Please see the Supplemental Methods for details.

### CUT&RUN

Please see the Supplemental Methods for details.

### Integrative CUT&RUN and RNA-seq data analysis

*CUT&RUN* peak calling (for EV-, WT-, and KKEA-cGAS–expressing MDA-MB-231 *cGAS^–/–^* cells) was performed using MACS2 (https://github.com/macs3-project/MACS; commit ID c544319). All downstream steps were performed using the R statistical language (version 4.3.2). Peaks from multiple replicates were combined into a consensus peak call using the rmspc package (c=2, alpha=0.05, weakThreshold = 1e-4, stringencyThreshold = 1e-8; version 1.8.0). Venn diagrams between WT and KKEA cGAS peaks were determined using the ChIPpeakAnno package (version 3.36) in R with the makeVennDiagram function (maxGap=500, connectedPeaks=merge). Metaplots were constructed using the computeMatrix function Deeptools and profileplyr in R to calculate the average signal value at each position.

Differential expression was performed using the DESeq2 (version 1.42) package with shrunken log_2_ fold-changes identified using lfcShrink (type=’apeglm’). Enriched pathways near cGAS peaks were identified by first defining shared peaks between WT and KKEA mutant cGAS *CUT&RUN*. These were then annotated to the nearest gene using plyranges in R, and the differential expression of these genes was obtained from the RNA-seq data comparing WT and EV. This input was used for gene set enrichment analysis and gene ontology analysis using the msigdbr (version 7.5.1) and clusterProfiler (version 4.10.1) R packages.

### RNA extraction and qRT-PCR

RNA was extracted using the RNA MiniPrep Super Kit (BioBasic, BS584) and eluted in 50 μL RNase-free water. RNA concentration was measured using a NanoDrop OneC, with at least 2 biological replicates per condition. cDNA was synthesized using the iScript cDNA Synthesis Kit (Bio-Rad, 1708891), and qRT-PCR was performed using iTaq Universal SYBR Green Supermix (Bio-Rad, 1725124) on a QuantStudio 6 Flex Real-Time PCR System (Thermo Fisher Scientific). Relative expression was calculated using the comparative Ct method and normalized to RNA18S or U6 small nuclear RNA. Statistical significance was assessed by 1-way ANOVA. Primers are listed in Supplemental Table 2.

### Soft agar assays

The anchorage-independent cell growth assays were performed as described (41, 66, 68). Soft agar colony formation assays were performed in 6-well plates using a 2-layer system consisting of 0.8% noble agar as the bottom layer and 0.4% agar containing 3 × 10^4^ or the indicated number of cells as the top layer. Cells were fed with 500 μL complete DMEM containing 10% FBS every 4 days. After 4–6 weeks, colonies were stained overnight with 1 mg/mL iodonitrotetrazolium chloride (Sigma, I10406) for visualization and quantification. Three independent experiments were performed.

### Mini-TurboID analysis

MDA-MB-231 *cGAS^–/–^* cells expressing miniTurbo-Flag-cGAS were cultured in 10 cm dishes and treated with 50 μM biotin or vehicle for 25 minutes before collection. Cells were washed twice with ice-cold PBS and lysed in buffer A (10 mM HEPES, pH 8.0; 10 mM KCl; 0.5% NP-40; 1 mM DTT; 1× protease inhibitor [PI]; and 1× phosphatase inhibitor [PPI]) for 10 minutes at room temperature. Following centrifugation at 21,000*g* for 3 minutes at 4°C, pellets were washed with Buffer A lacking DTT and lysed in RIPA buffer containing 2 M urea, 1× PI, and 1× PPI for 15 minutes on ice, followed by sonication (5 pulses, 30% output). Lysates were centrifuged at 15,000*g* for 10 minutes at 4°C, and 5% of each supernatant was saved as input. Lysates were incubated overnight at 4°C with 100 μL streptavidin T1 Dynabeads (Thermo Fisher Scientific) pre-equilibrated in RIPA/urea buffer. Beads were washed 4 times with lysis buffer and 3 times with 50 mM ammonium bicarbonate (ABC), pH 7.8, then resuspended in 50 μL ABC and frozen for proteomic analysis by the UNC Metabolomics and Proteomics (MAP) Core Facility.

### FACS

For cell size analysis, cells were washed with PBS, trypsinized, washed twice with PBS containing 1% FBS, and resuspended at 5 × 10^6^ cells/mL. Aliquots (100 μL) were stained with 10 μL propidium iodide solution (BioLegend, 42301) for 15 minutes at room temperature in the dark, followed by addition of 400 μL PBS. Data were acquired using an Attune NxT flow cytometer (Thermo Fisher Scientific) and analyzed with FlowJo.

### AP-1 TF-PROTAC synthesis

The synthesis of AP-1 TF-PROTACs is as described (24). Briefly, bicyclo[6.1.0]non-4-yne-modified VHL ligands (VHLL-X-BCN; 200 μM) were conjugated to the synthetic AP-1–binding oligonucleotide (TGACTCATTTTTGAGTCA/3AzideN/; 50 μM) in distilled water for 12 hours at room temperature. Following annealing, 500 ng oligonucleotide was resolved by 20% native PAGE in TBE at 130 V for 1.5 hours, stained with ethidium bromide, and imaged using an Azure 400 system (Azure Biosystems). Indicated AP-1 TF-PROTACs (5 μg/mL) were transfected into cells for 24 hours before collection.

### Immunofluorescence

Cells grown on glass coverslips were fixed with 4% paraformaldehyde in PBS for 10 minutes at room temperature and permeabilized with 0.2% Triton X-100 for 20 minutes. After blocking with 5% BSA and 0.1% Triton X-100 in PBS for 1 hour, cells were incubated with primary antibodies — Alexa Fluor 488 Goat anti-Mouse IgG (H+L) Invitrogen, Thermo Fisher Scientific, catalog A-11001; Alexa Fluor 488 Goat anti-Rabbit IgG (H+L) Invitrogen, Thermo Fisher Scientific, catalog A-11008; Alexa Fluor 594 Goat anti-Mouse IgG (H+L) Invitrogen, Thermo Fisher Scientific, catalog A-11005; Alexa Fluor 594 Goat anti-Rabbit IgG (H+L) Invitrogen, Thermo Fisher Scientific, catalog A-11012 — overnight at 4°C and secondary antibodies (Supplemental Table 1) for 1 hour at room temperature. Coverslips were mounted with ProLong Gold antifade reagent (Invitrogen) and imaged using a Keyence BZ-X700 microscope.

### Immunohistochemistry

Xenograft tumors were fixed in 10% formalin for 2 days, followed by 70% ethanol for 1 day. The UNC Translational Pathology Laboratory facility prepared 4 μm sections for immunohistochemistry, which was performed as previously described (67, 69). Briefly, sections were blocked for 1 hour, incubated with primary antibody — Biotinylated Horse Anti-Rabbit IgG (H+L) Vector Laboratories catalog BA-1100-1.5 — overnight at 4°C and secondary antibodies (Supplemental Table 1) for 1 hour at room temperature, followed by ABC reagent for 45 minutes and DAB development. Sections were counterstained with hematoxylin and imaged using a Keyence BZ-X700 microscope.

### Acetyl-CoA assay

Acetyl-CoA levels were measured using the Acetyl-CoA Assay Kit (MAK039, Sigma) according to the manufacturer’s instructions. Briefly, cells were harvested and homogenized on ice in assay buffer, followed by perchloric acid precipitation to deproteinize samples. The pH was adjusted to 6.5 to 8.0, and 10 μL of each sample was added in triplicate to a 96-well plate and brought to 50 μL with assay buffer. After addition of 10 μL acetyl-CoA quencher, reaction mix was added, and samples were mixed and incubated at 37°C for 10 minutes. Fluorescence was measured using a BioTek Cytation 5 Cell Imaging Reader (excitation/emission = 535/587 nm).

### Protein purification

Recombinant GST-ACAA2 was expressed in *E*. *coli* BL21 by isopropyl β-d-1-thiogalactopyranoside induction for 16 hours at 16°C. Bacterial pellets were resuspended in PBS containing protease inhibitors and lysed by sonication. Following centrifugation at 16,000*g* for 30 minutes, the supernatant was incubated with prewashed GST-Sepharose beads (GE Healthcare) for 4 hours at 4°C. Bound proteins were eluted with 20 mM reduced glutathione in 50 mM Tris-HCl (pH 8.0).

### [13C]-palmitate tracing

Indicated MDA-MB-231 cells were seeded in 6-well plates. Cell culture medium was replaced with 2 mL fresh medium supplemented with 100 μM [^13^C_16_]-palmitic acid the next day. Cells were incubated for 1 hour in a CO_2_ incubator. After incubation, medium was aspirated and briefly washed (twice) with 2 mL of ice-cold 0.9% NaCl solution (prepared by dissolving 9 g of NaCl in 1 L of HPLC-grade water). Plates were immediately placed on dry ice, and 1 mL of 80% methanol/water (both HPLC grade, precooled in a –20°C freezer for at least 1 hour) was added to each well. The plates were transferred to a –80°C freezer and left there for 15 minutes to ensure enzymatic activity was fully inactivated. Cells were scraped into the 80% methanol/water solution on dry ice and transferred into 1.5 mL tubes placed on regular ice. Tubes were vortexed thoroughly for at least 1 minute to ensure proper extraction, and samples were centrifuged at 20,000*g* for 10 minutes at 4°C to precipitate cellular proteins. Supernatant was transferred to liquid chromatography (LC) vials for LC-MS analysis.

### LC-MS analysis of polar metabolites

The analysis of metabolites was performed using Vanquish UHPLC coupled to Orbitrap Exploris 480 mass spectrometer (Thermo Fisher Scientific). A total of 3 μL metabolite extract was loaded onto Xbridge amide column (100 × 2.1 mm, 3.5 μm; Waters Corporation) at 25°C. Mobile phase A was water with 5 mM ammonium acetate (pH 6.8) with 3% methanol, and mobile phase B was 100% acetonitrile with 3% methanol. Linear gradient was 0 minute, 85% B; 1.5 minutes, 85% B; 5.5 minutes, 35% B; 6.9 minutes, 35% B; 10.5 minutes, 35% B; 10.6 minutes, 10% B; 12.5 minutes, 10% B; 13.5 minutes, 85% B; 17.9 minutes, 85% B; 18 minutes, 85% B; 20 minutes, 85% B. The flow rate is 0–5.5 minutes, 0.11 mL/min; 6.9–10.5 minutes, 0.13 mL/min; 10.6–17.9 minutes, 0.25 mL/min; 18–20 minutes, 0.11 mL/min. The parameters of Orbitrap Exploris 480 were as listed: vaporizer temperature, 200°C; ion transfer tube temperature, 300°C; sheath gas, 35; auxiliary gas, 7; sweep gas, 1; spray voltage, 3.5 kV for positive mode and 3 kV for negative mode; RF-lens (%), 30; resolution was set at 60,000 (at *m/z* 200); scan range, 70 to 900 (*m/z*); positive/negative switching mode. Unlabeled or ^13^C-labeled metabolite analysis was performed using Sieve (Thermo Fisher Scientific) based on theoretical *m/z* and retention time determined based on standard compounds. Integrated peak areas of unlabeled or ^13^C-labeled metabolites were used to calculate the fold-change of metabolites in different samples. To assess relative changes in total metabolite levels, the sum of peak areas of unlabeled and detected ¹³C-labeled metabolites was used.

### AlphaFold3-based prediction of human cGAS/Fos-L1/JunB interactions

Protein-protein interactions between cGAS (UniProt: Q8N884), FosL1 (UniProt: P15407), and JunB (UniProt: P17275) were predicted using AlphaFold3 3.0.1 (70), run on a local UNC-Chapel Hill computing cluster. Full-length, terminal intrinsically disordered region–truncated, and domain-truncated sequences of human proteins were input as heterodimeric complexes, with default parameters for multiple sequence alignment generation using MMseqs2 and 6 recycled iterations for structural refinement. A total of 20 models were generated per run, and predictions were repeated across 5 random seeds to enhance sampling diversity. The resulting structures were ranked using an in-house Python script that evaluated the interface predicted local distance difference test (ipLDDT) score (average pLDDT of residues within 8 Å of the binding interface) and minimized root-mean-square deviation (RMSD) alignments between structures (performed with PyMOL’s align function) before and after binding for consistency assessment. The top 8 ranked models were selected for further manual processing, wherein the best model was selected based on a high pLDDT score (>0.85) for noninterface regions with known structural motifs, and the number of polar and nonpolar contacts in the interface, assessed via PLIP 2.2.2 with cutoffs of 3.5 Å for hydrogen bonds and 4.5 Å for salt bridges. Each model would then be manually inspected, and those that most closely matched experimental results were selected for further refinement.

### Interface evaluation and refinement

Top-ranking cGAS/FosL1/JunB complexes from AlphaFold3 were refined using ChimeraX ISOLDE with CHARMM36m, employing restrained interactive MD with side-chain rotamer sampling and limited backbone flexibility. Harmonic restraints (0.5–1.0 kcal/mol/Å^2^) were applied to Cα atoms to accommodate local interface rearrangements while preserving the predicted geometry. Three independent refinement trajectories were performed for each complex. Backbone integrity was assessed by Cα RMSD relative to the starting AlphaFold3 model, excluding models with deviations >1.2 Å. Convergence was evaluated by restraint potential energy and pairwise Cα RMSD across trajectories. Buried surface area was calculated for analysis but not used for filtering. The lowest-energy converged model was selected for further analysis.

### cGAS/AP-1 complex equilibration

The refined cGAS/FosL1/JunB heterotrimer was prepared for MD simulation using VMD version 1.9.4 to generate protein structure files and solvate the system in a cubic TIP3P water box with a minimum 10 Å padding from the protein surface. The system was neutralized and ionized to 0.15 M NaCl. Parameters were assigned using the CHARMM36m force field, and simulation input files were generated using CHARMM-GUI. Simulations were performed using NAMD version 3.0 with graphics processing unit acceleration.

The system underwent 10,000 steps of conjugate gradient energy minimization followed by restrained equilibration consisting of 1 ns in the constant number, volume, temperature ensemble at 310 K and 2 ns in the constant number, pressure, temperature (NPT) ensemble at 1 atm using the Langevin piston method with gradual release of positional restraints. This was followed by additional unrestrained NPT equilibration for 2 ns, for a total simulation time of 5 ns. Backbone stability was assessed by monitoring Cα RMSD relative to the starting model throughout equilibration. The equilibration phase was designed to further relieve steric clashes, optimize side-chain rotamer conformations, and allow solvent reorganization without performing extended production simulations. No long-timescale dynamic analyses were conducted. The final equilibrated structure was extracted as a CIF file for subsequent structural and interface analysis.

### MTT cell viability assay

A total of 2,000 cells were seeded per well in 96-well plates. At designated time points, 10 μL MTT solution was added, and cells were incubated at 37°C with 5% CO_2_ for 4 hours. The medium was then removed, and 100 μL DMSO was added to dissolve formazan crystals, followed by a 10-minute incubation at 37°C. Absorbance at 540 nm was measured using a BioTek Cytation 5 Cell Imaging Reader after thorough mixing.

### Animals

#### Mouse xenograft assays.

Mouse xenograft assays were performed as previously described (65, 67). For xenograft growth experiments, the indicated number of MDA-MB-231 or HeLa cells in PBS were injected into the flanks of female nude mice (The Jackson Laboratory, 4 weeks old). Tumor size was measured every 3 days using a digital caliper, and tumor volume was calculated using the formula L × W² × 0.5, where L represents the longest diameter and W the shortest. After the designated experimental period, mice were sacrificed, and tumors were dissected and weighed. Where indicated, tumors were fixed in formalin and processed with tumor slices for indicated H&E staining.

#### Mouse lung assays.

Six-month-old, age-matched *Wt* and *cGas*-KO C57BL/6 mice (The Jackson Laboratory; maintained by the P. Liu lab) were used for lung studies. Capivasertib (50 mg/kg; CAS 1143532-39-1) was administered by oral gavage once daily for 4 days, and H-151 (7 mg/kg; CAS 941987-60-6) was administered intraperitoneally every 2 days for 2 doses. Vehicle controls received the corresponding vehicle on the same schedule and route. At the end of treatment, mice were euthanized, and lungs were collected for analysis.

#### BAL and lung histology.

Four-month-old, age-matched *Wt* and *cGas*-KO mice underwent BAL of the right lung lobes, excluding the auxiliary lobe for wet/dry weight analysis (see below), to assess cellular infiltration by total and differential cell counts. The left lung lobe was collected for histopathological analysis using validated protocols (71, 72) that permit both assessments in the same mouse. Lungs were sectioned every 2 mm from the hilum to systematically sample proximal, intermediate, and distal levels along the main stem bronchus. Histopathological evaluation was performed by an experienced investigator without knowledge of genotype.

#### Lung wet/dry ratio measurements.

The extrapulmonary bronchus to the auxiliary lobe was ligated to prevent BAL fluid leakage. The lobe was then dissected, blotted dry, placed in preweighed aluminum boats, and weighed at 30, 40, 50, and 60 seconds to extrapolate evaporative water loss and determine the initial wet weight (73). Lungs were desiccated for 16 hours at 90°C and then reweighed to obtain dry weights and determine wet/dry ratios.

### Statistics

Statistical analyses were performed using SPSS 11.5. Data are presented as mean ± SD from at least 2 or 3 independent experiments, as indicated in the figure legends. One-way ANOVA was used to assess differences between control and experimental groups, with *P* ≤ 0.05 considered statistically significant.

### Study approval

All mouse experiments were reviewed and approved by the UNC Institutional Animal Care and Use Committee under protocols #19-031 (continued as #25-017) and #25-085.

### Data availability

Reagents generated in this study are available upon request to the corresponding author. *CUT&RUN* data have been deposited to GEO (GSE289114). MDA-MB-231-cGAS-KO+cGAS-WT, cGAS-F491A/F503A, and shJunB RNA-seq data have been deposited to GEO (GSE337117). Micro-C data have been deposited to GEO (GSE342461). This paper does not report original code. Additional information required to reanalyze data reported in this paper is available in Supporting Data Values.

## Manuscript information

### Author contributions.

PL conceptualized the study. YD, MJN, AJH, HK, JMS, JL, Yan Zhang, JJ, LEH, DHP, XL, and JR developed the methodology. YD, MJN, ZZ, AJH, LY, JH, TR, PAM, HK, Yanqiong Zhang, QH, MJZ, JGCK, JFD, XQ, and HUK conducted the investigation. YD, MJN, AJH, TR, HK, XL, and JR performed visualization. BDS, TR, DHP, JH, XL, and PL acquired funding. PL administered the project. JMS, ALB, CDJ, DHP, BDS, and PL supervised the work. YD and PL wrote the original draft. YD, MJN, ZZ, AJH, LY, QH, JH, TR, PAM, HK, JMS, Yanqiong Zhang, ALB, MJZ, JGCK, JFD, JL, Yan Zhang, XQ, HUK, JJ, CDJ, LEH, DHP, XL, JR, BDS, and PL reviewed and edited the manuscript.

### AI disclosure.

PromptLab at UNC-Chapel Hill was used for proofreading and removing grammar mistakes.

### Conflict of interest.

BDS is a cofounder and board member of EpiCypher, Inc. JJ is an equity shareholder in Cullgen, Inc. and a consultant for Cullgen, Inc. JJ was a cofounder of Cullgen, Inc.; a scientific cofounder and scientific advisory board member of Onsero Therapeutics, Inc.; and a consultant for EpiCypher, Inc. and Accent Therapeutics, Inc. JJ’s laboratory received research funds from Celgene Corporation; Levo Therapeutics, Inc.; Cullgen, Inc.; and Cullinan Therapeutics, Inc.

### Funding.

This study was supported by the following:

- NIH National Cancer Institute Center Core Support Grant (CA16086), supporting the UNC Lineberger Animal Study Core within the UNC Lineberger Comprehensive Cancer Center.
- Cystic Fibrosis Foundation Research and Development Program (ESTHER24R0), supporting the Pulmonary Inhalation Research Core.
- NIH National Institute of Diabetes and Digestive and Kidney Diseases Cystic Fibrosis Research and Translation Core Center grant (P30DK065988-21), supporting the Pulmonary Inhalation Research Core.
- NIH grant R01CA244825 (PL).
- NIH grant R01AG095618 (PL).
- NIH grant R35GM126900 (BDS).
- NIH grant R35GM128645 (DHP).
- NIH grant R01CA271603 (DHP).
- NIH grant 1R35GM150985 (XL).
- Mary Kay Ash Foundation Cancer Research Grant (PL).
- NIGMS grant T32GM067553 (TR).
- North Carolina State University IBMP Fellowship (JH).

This work is the result of NIH funding, in whole or in part, and is subject to the NIH Public Access Policy. Through acceptance of this federal funding, the NIH has been given a right to make the work publicly available in PubMed Central.

## Supplementary Material

Supplemental data

Unedited blot and gel images

Supporting data values

## Figures and Tables

**Figure 1:**
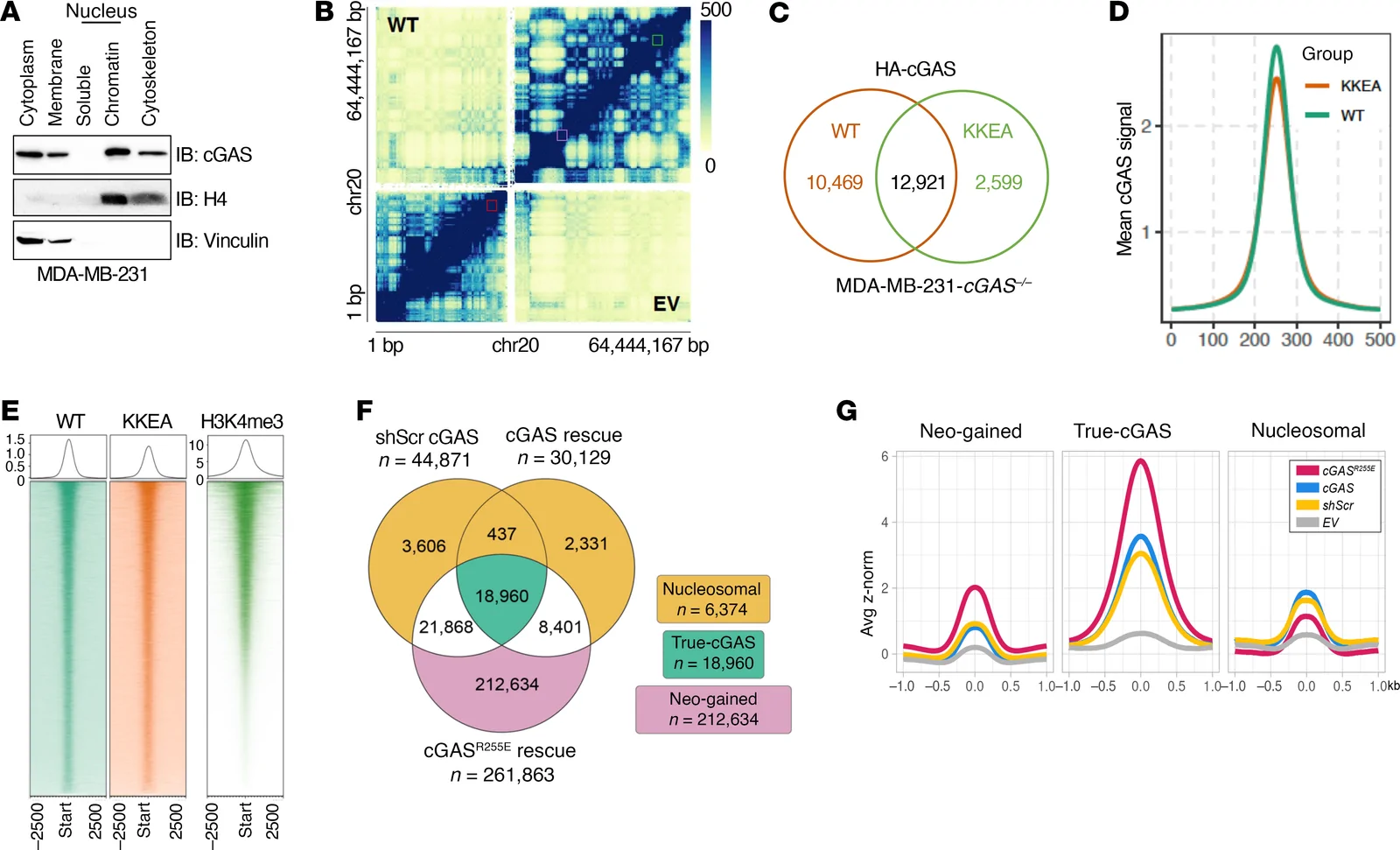
cGAS *CUT&RUN* analysis reveals genome-wide cGAS distributions that are independent of its DNA-binding and nucleosome-binding abilities. (**A**) Immunoblot (IB) analysis of indicated cellular fractions from MDA-MB-231 cells. (**B**) Chromatin interaction heatmaps of Micro-C data in the chr20 region. (**C**) A Venn diagram showing identified cGAS *CUT&RUN* peaks in indicated MDA-MB-231 cells. (**D**) cGAS *CUT&RUN* signals are enriched in a narrower window from both WT- and KKEA-cGAS–expressing MDA-MB-231 cells. (**E**) A heatmap for all indicated *CUT&RUN* peaks spanning –2.5 to 2.5 kb regions. H3K4me3, histone H3 trimethylated at lysine 4. (**F**) A Venn diagram showing identified cGAS *CUT&RUN* peaks in indicated MDA-MB-231 cells. sh, shRNA. (**G**) Average signal plots of *z*-normalized cGAS *CUT&RUN* signal from indicated MDA-MB-231 cells ± 1 kb around peak centers for each cGAS-binding category.

**Figure 2:**
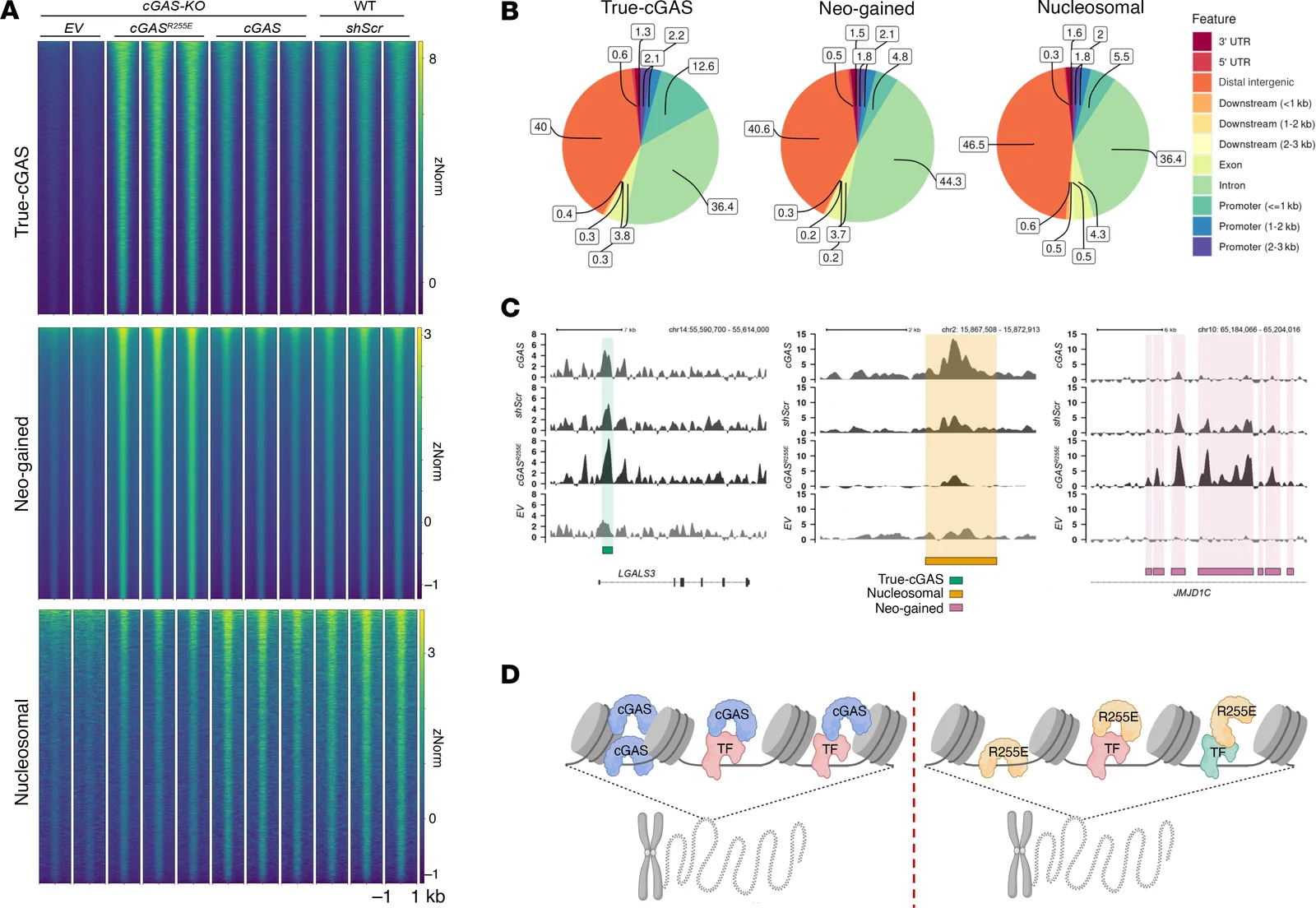
Nuclear cGAS localizes to genomic regions beyond nucleosomes. (**A**) Heatmaps of *z*-normalized signal for all indicated *CUT&RUN* peaks spanning –1 to 1 kb regions. (**B**) Pie charts showing the distributions of cGAS *CUT&RUN* peaks in each indicated category. (**C**) Representative genome browser views for indicated cGAS *CUT&RUN* peaks at indicated loci. Tracks are replicate averages of the *z*-normalized signal. Bars and highlights mark labeled cGAS peak categories. (**D**) A schematic model illustrating that nuclear cGAS exists in 3 distinct states: (i) tethered to nucleosomes via interaction with H2A/H2B acidic patches, (ii) associated with transcription factors to regulate transcription, and (iii) engaging both nucleosomal acidic patches and transcription factors simultaneously.

**Figure 3:**
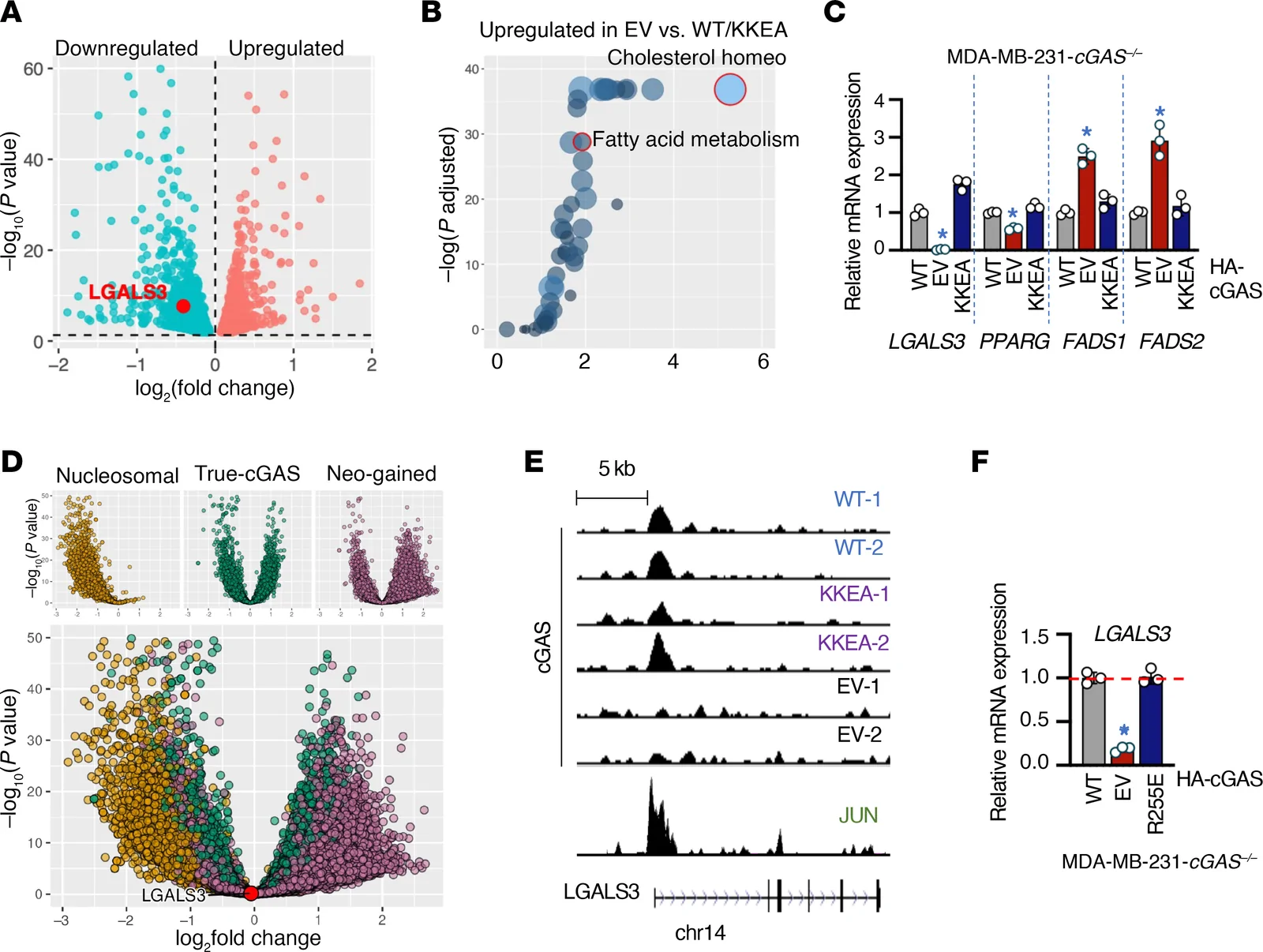
cGAS is recruited to chromatin by AP-1 transcription factors. (**A**) A volcano plot from RNA-seq analysis displaying overlapping hits between WT- and KKEA-cGAS–expressing MDA-MB-231-*cGAS^–/–^* cells compared with EV-expressing cells. (**B**) A volcano plot showing the integrative analysis results from both cGAS *CUT&RUN* and RNA-seq analysis from EV-, WT- and KKEA-cGAS–expressing MDA-MB-231-*cGAS^–/–^* cells. (**C** and **F**) RT-PCR analysis of indicated gene targets from indicated MDA-MB-231 cells. Error bars were calculated as mean ± SD. *n* = 3. *P < 0.05 (1-way ANOVA test). The red dashed line indicates the normalized expression level of the control group, set to 1. (**D**) A volcano plot from cGAS *CUT&RUN* analysis showing difference in counts between WT- versus R255E-cGAS rescue within all cGAS peaks. The closer to the center of the *x* axis, the more likely the target is a true cGAS transcriptional target. The *LGALS3* promoter peak is labeled in red. Peaks are colored by cGAS-binding category. *Y* axis is limited to 0–50 to improve visualization (102 points removed). (**E**) Representative genome browser views for indicated cGAS *CUT&RUN* peaks at the *LGALS3* locus.

**Figure 4:**
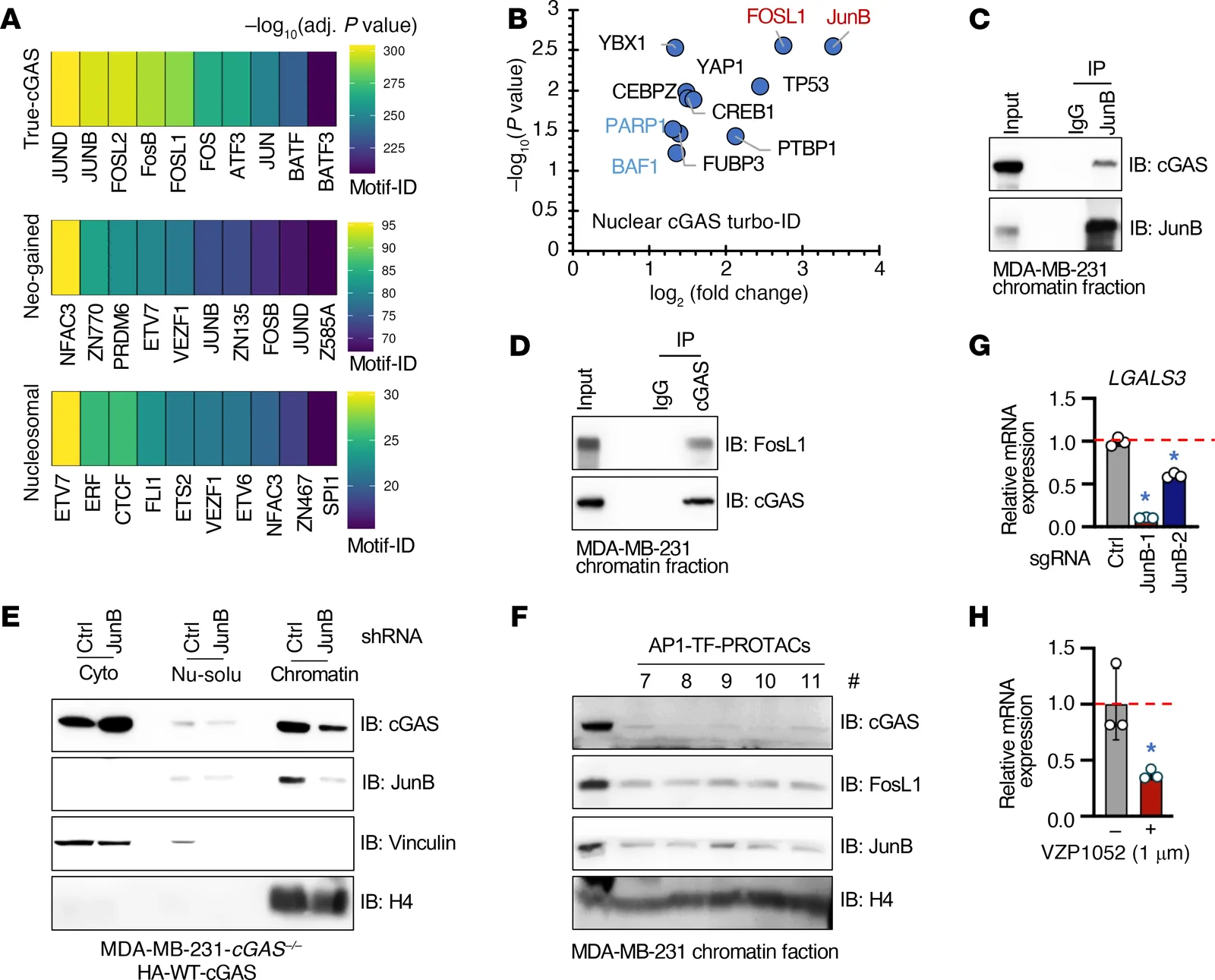
cGAS binds AP-1 transcription factors. (**A**) Heatmap of –log_10_(adj.pvalue) for top 10 enriched motifs identified by AME for sequences within cGAS *CUT&RUN* peaks in each group. (**B**) A volcano plot showing the top transcription factor hits from nuclear cGAS TurboID analysis from MDA-MB-231 cells. (**C** and **D**) IB analysis of indicated immunoprecipitations (IPs) and input from MDA-MB-231 cell chromatin fractions. (**E**) IB analysis of indicated cellular fractions from indicated MDA-MB-231 cells. (**F**) IB analysis of chromatin fractions from MDA-MB-231 cells transfected with indicated AP-1 TF-PROTACs. (**G** and **H**) RT-PCR analysis of indicated target genes from indicated MDA-MB-231 cells (**G**) or from MDA-MB-231 cells treated with 1 μM VZP1052 for 24 hours prior to cell collection (**H**).

**Figure 5:**
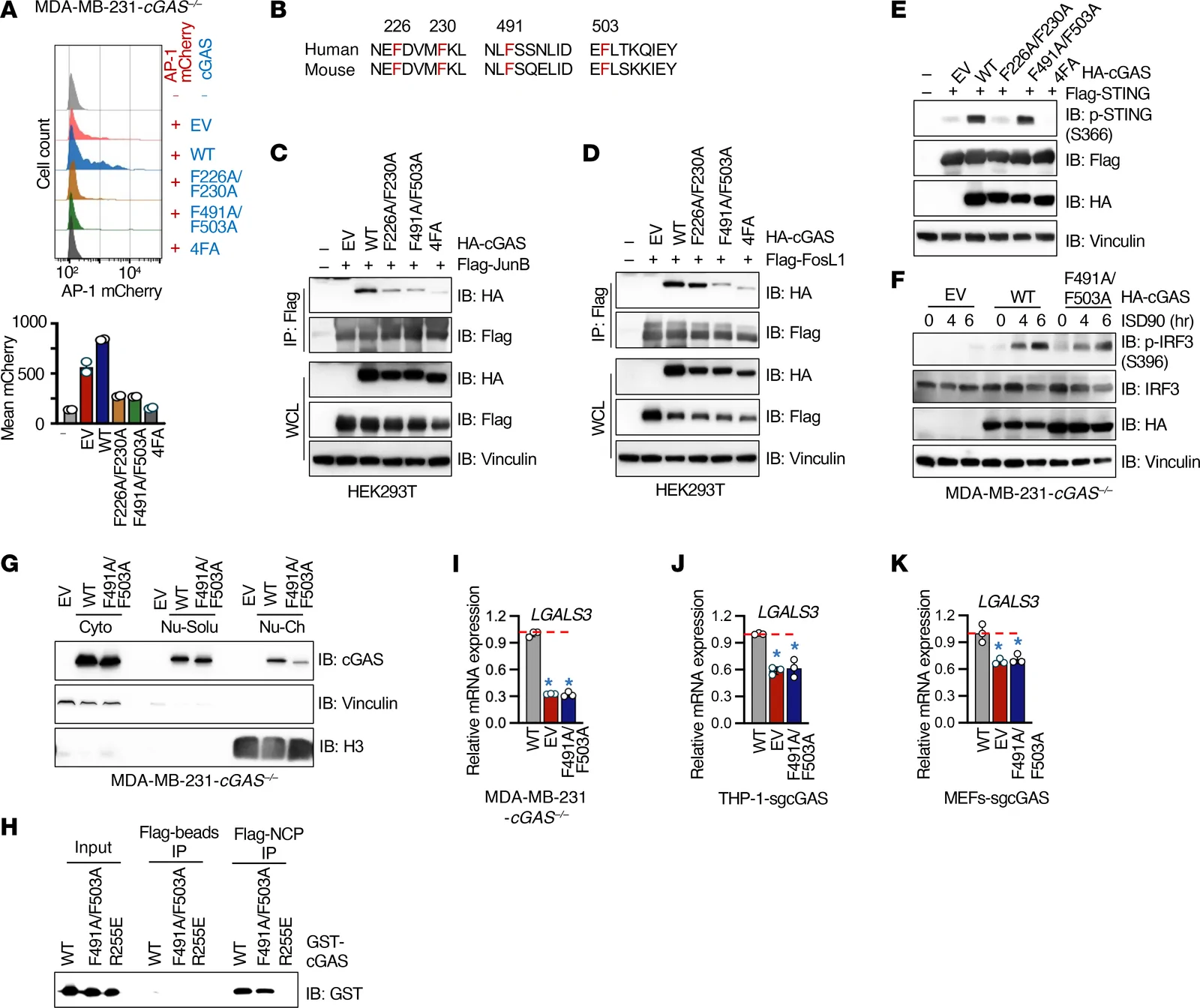
A TAD in cGAS is responsible for AP-1 recognition but dispensable for canonical cGAS activation in innate immunity. (**A**) Representative FACS analysis of AP-1 cherry reporter activity in indicated MDA-MB-231 cells and quantified. Error bars were calculated as mean ± SD. *n* = 3. (**B**) Protein sequence alignment of human and mouse cGAS with critical Phe residues in possible TADs in red. (**C** and **D**) IB analysis of Flag-IPs and whole-cell lysate (WCL) from HEK293T cells transfected with indicated DNA constructs. (**E**) IB analysis of WCL from HEK293T cells transfected with indicated DNA constructs. (**F**) IB analysis of indicated MDA-MB-231 cells transfected with 5 μg/mL ISD90 for indicated time periods. (**G**) IB analysis of indicated cellular fractions from indicated MDA-MB-231 cells. (**H**) IB analysis of indicated Flag-NCP-IPs with indicated bacterially purified GST-cGAS recombinant proteins. (**I**–**K**) RT-PCR analysis of mRNA expression of *LGALS3* from indicated cells. Error bars represent mean ± SD. *n* = 3. *P < 0.05 (1-way ANOVA test).

**Figure 6:**
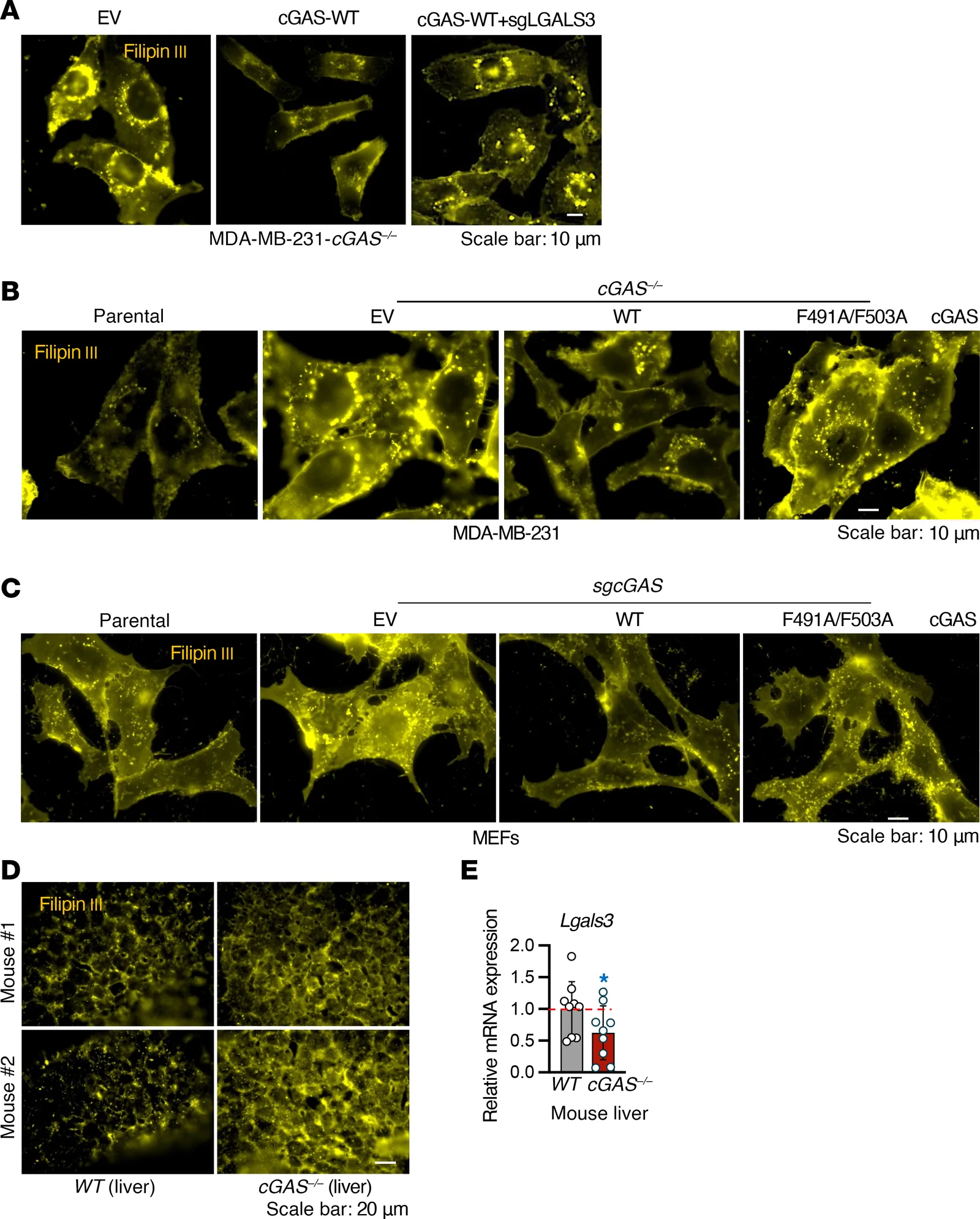
cGAS-driven transcription of LGALS3 inhibits cholesterol production. (**A**–**D**) Representative images for Filippin III staining for free cholesterol in indicated MDA-MB-231 cells (**A** and **B**), MEF cells (**C**), or liver slides from indicated C57BL/6 mice (**D**). (**E**) RT-PCR analysis of mRNA expression of *Lgals3* from indicated mouse livers. Error bars represent mean ± SD. *n* = 3. *P < 0.05 (1-way ANOVA test). The red dashed line indicates the normalized expression level of the control group, set to 1.

**Figure 7:**
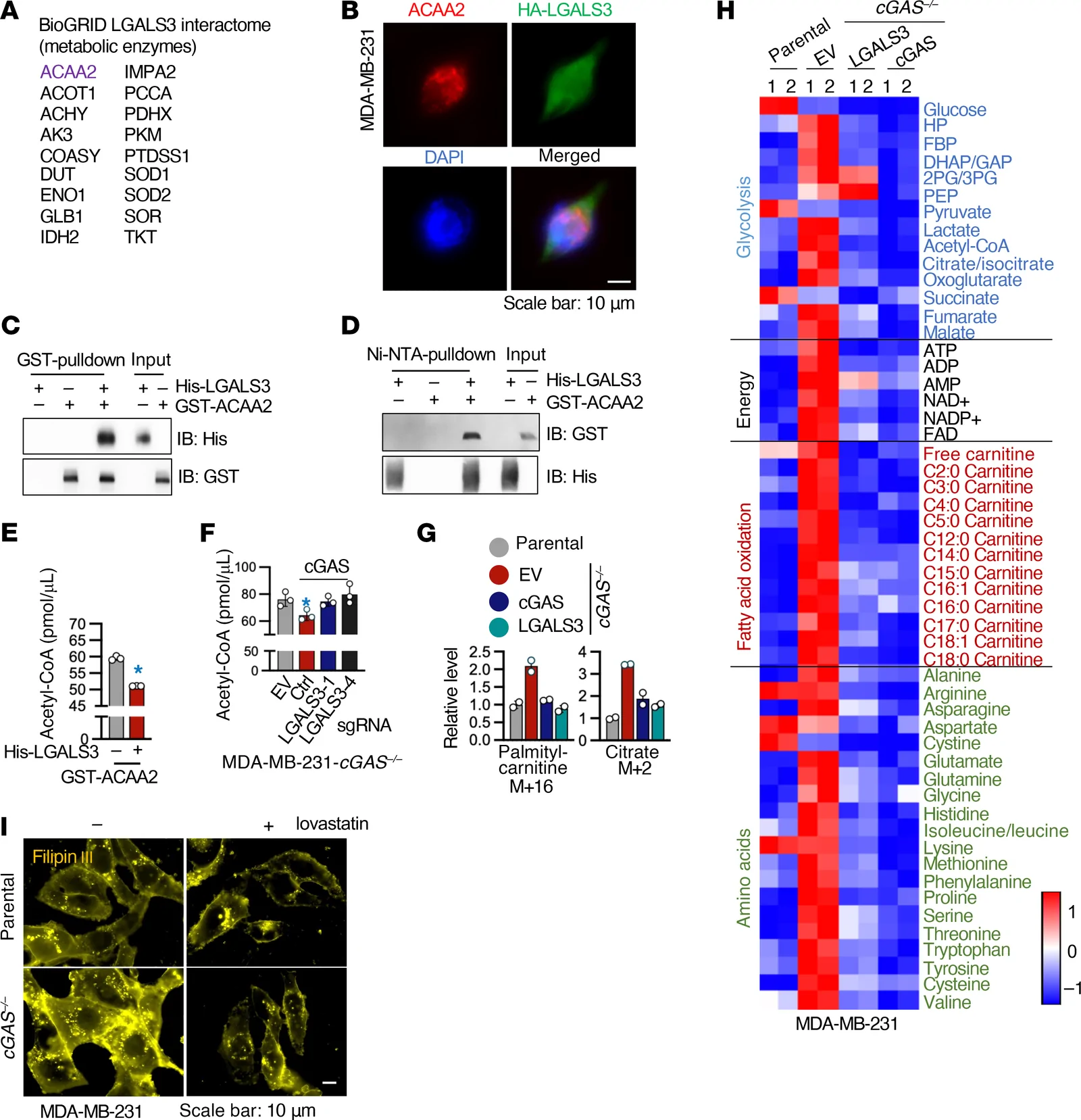
cGAS-driven transcription of LGALS3 inhibits ACAA2 activity, suppressing fatty acid oxidation. (**A**) A list of LGALS3-binding proteins from BioGRID. (**B**) Representative confocal immunofluorescence (IF) images for ACAA2 and LGALS3 in MDA-MB-231 cells. (**C** and **D**) IB analysis of pulldowns and inputs using indicated bacterially purified recombinant proteins. (**E** and **F**) Representative acetyl-CoA ELISAs measuring acetyl-CoA levels in either in vitro ACAA2 reactions with recombinant LGALS3 proteins (**E**) or indicated MDA-MB-231 cells (**F**). (**G**) Normalized levels of [^13^C_2_]-citrate (citrate M+2) and [^13^C_16_]-palmityl-carnitine in indicated MDA-MB-231 cells relative to parental cells. Data are shown as individual values with mean, *n* = 2 independent biological replicates. No statistical analysis was performed because *n* < 3. (**H**) A heatmap quantifying indicated metabolites in indicated MDA-MB-231 cells with ^13^C-palmitate tracing for 2 hours. HP, hexose phosphate; FBP, fructose bisphosphate; DHAP/GAP, dihydroxyacetone phosphate/glyceraldehyde-3-phosphate; 2PG/3PG, 2-phosphoglycerate/3-phosphoglycerate; PEP, phosphoenolpyruvate. (**I**) Representative Filippin III staining images for free cholesterol in indicated MDA-MB-231 cells. Where indicated, 5 μM lovastatin was used to treat cells for 6 hours prior to staining.

**Figure 8:**
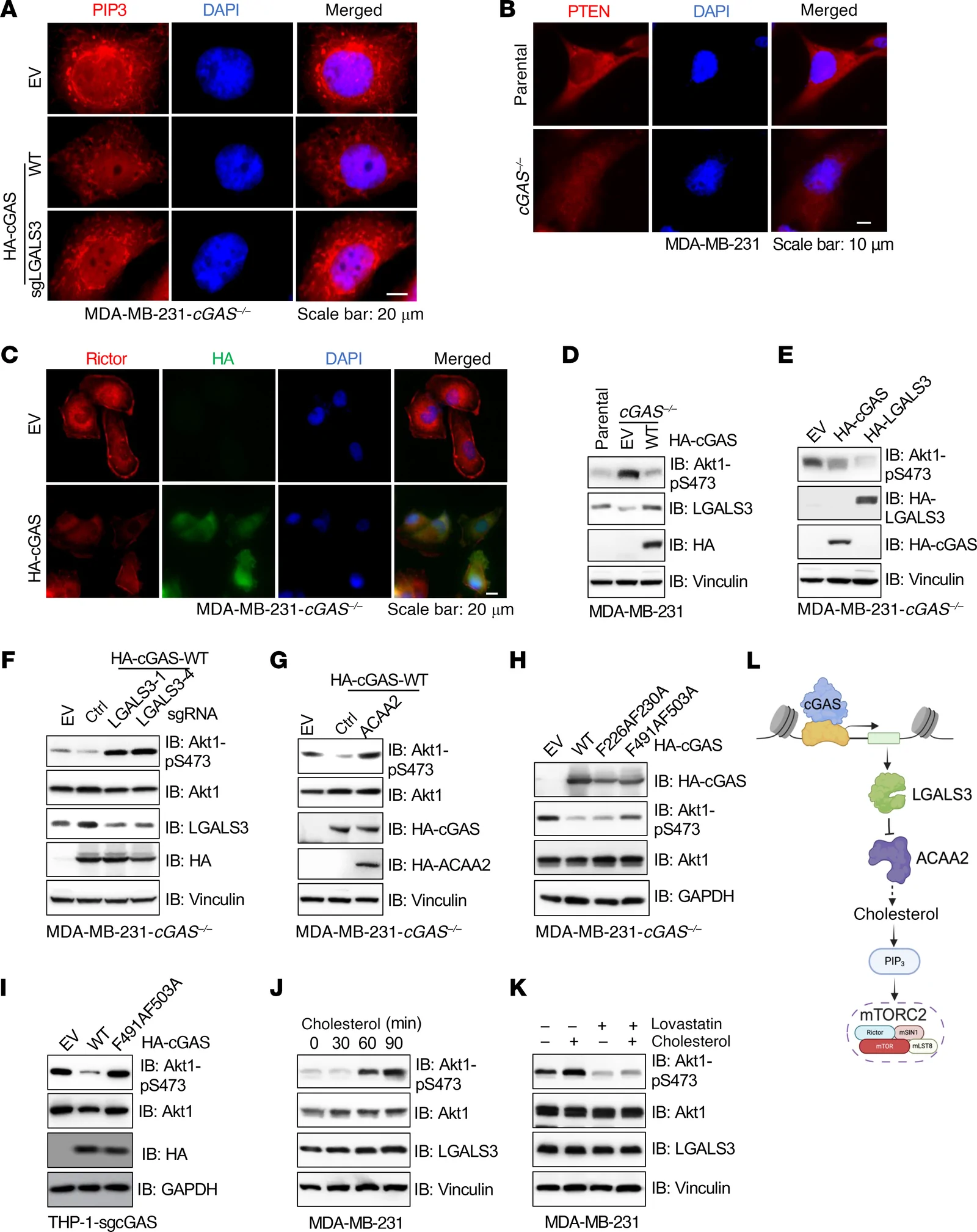
cGAS/LGALS3-regulated cholesterol changes modulate PI(3,4,5)P_3_ levels and mTORC2/Akt activation. (**A**) Representative confocal images of PI(3,4,5)P_3_ staining of indicated MDA-MB-231 cells. (**B** and **C**) Representative confocal images of PTEN staining (**B**) or Rictor staining (**C**) of indicated MDA-MB-231 cells. (**D**–**H**) IB analysis of WCL from indicated MDA-MB-231 cells. (**I**) IB analysis of WCL from indicated THP1 cells. (**J**) IB analysis of WCL from indicated MDA-MB-231 cells. Where indicated, 100 μM cholesterol was added to cell culture for indicated time periods prior to cell collection. (**K**) IB analysis of WCL from indicated MDA-MB-231 cells. Where indicated, cells were pretreated with 5 μM lovastatin for 2 hours followed by addition of 100 μM cholesterol for 1.5 hours prior to cell collection. (**L**) A schematic illustration of the proposed model: cGAS promotes LGALS3 transcription, which binds to and suppresses ACAA2 activity, leading to reduced cellular acetyl-CoA levels, decreased cholesterol production, and diminished mTORC2/Akt activation.

**Figure 9:**
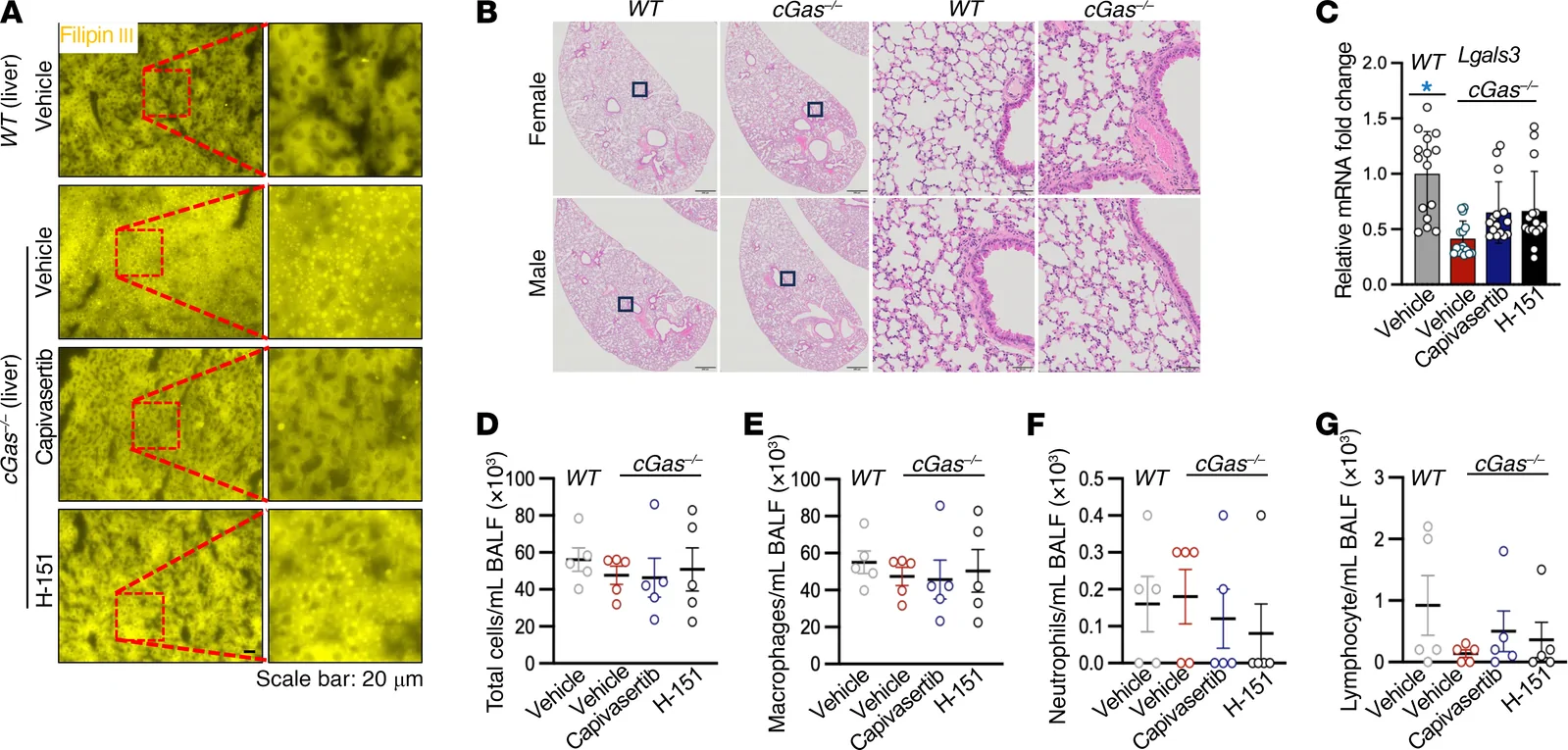
Inhibition of Akt, but not STING, reverses cholesterol accumulation in cGAS-deficient mouse livers. (**A**) Representative images for Filippin III staining for free cholesterol in indicated mouse livers. Where indicated, indicated C57BL/6 mice were treated with Akt inhibitor capivasertib or STING inhibitor H-151 prior to liver collection. Refer to Methods for details. (**B**) Lung histopathology in 4-month-old WT and *cGAS*-KO mice. Representative H&E-stained lung sections from both sexes show preserved airway and alveolar architecture. Left: low magnification, intermediate section of the left lung lobe, scale bar = 0.5 mm. Right: high magnification of insets, scale bar = 50 μm. (**C**) RT-PCR analysis of *Lgals3* mRNA expression in lungs from indicated mice with indicated treatments. Error bars represent mean ± SD; *n* = 3. *P < 0.05 by 1-way ANOVA. (**D**–**G**) Bronchoalveolar lavage (BAL) cell composition in *cGas*-KO and WT littermates under indicated treatment conditions. (**D**) Total BAL cell counts. (**E**–**G**) Differential cell counts showing macrophages, neutrophils, and lymphocytes.

**Figure 10:**
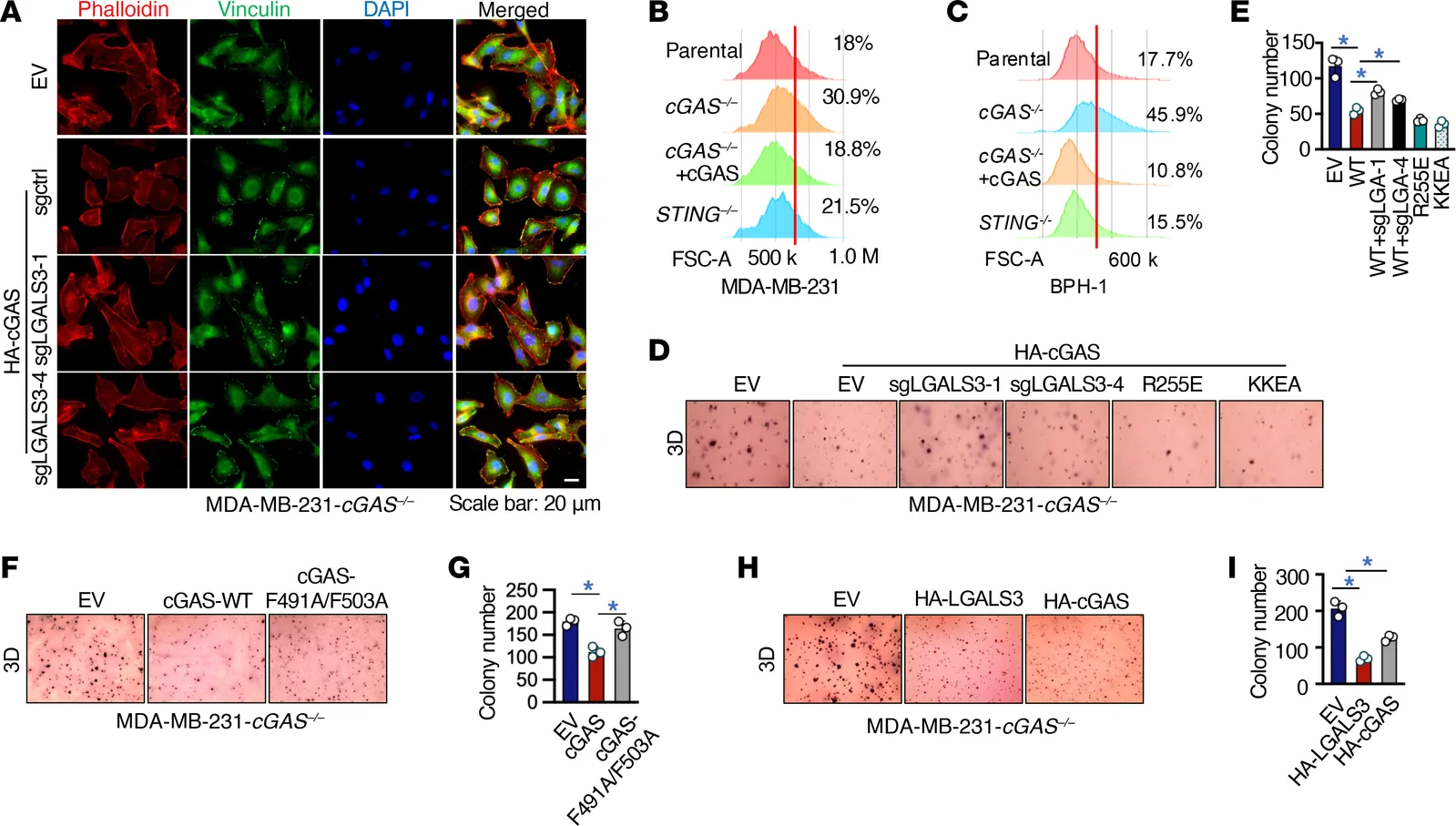
The transcriptional activity of cGAS influences cell size and 3D growth. (**A**) Representative IF images of phalloidin and vinculin in the indicated MDA-MB-231 cells. (**B** and **C**) Representative FACS analysis showing cell size distributions in the indicated MDA-MB-231 cells. (**D**–**I**) Representative images of cell colonies grown in soft agar using the indicated MDA-MB-231 cells, with quantification shown in **E**, **G**, and **I**. Error bars represent mean ± SD. *n* = 3. *P < 0.05 (1-way ANOVA test).

**Figure 11:**
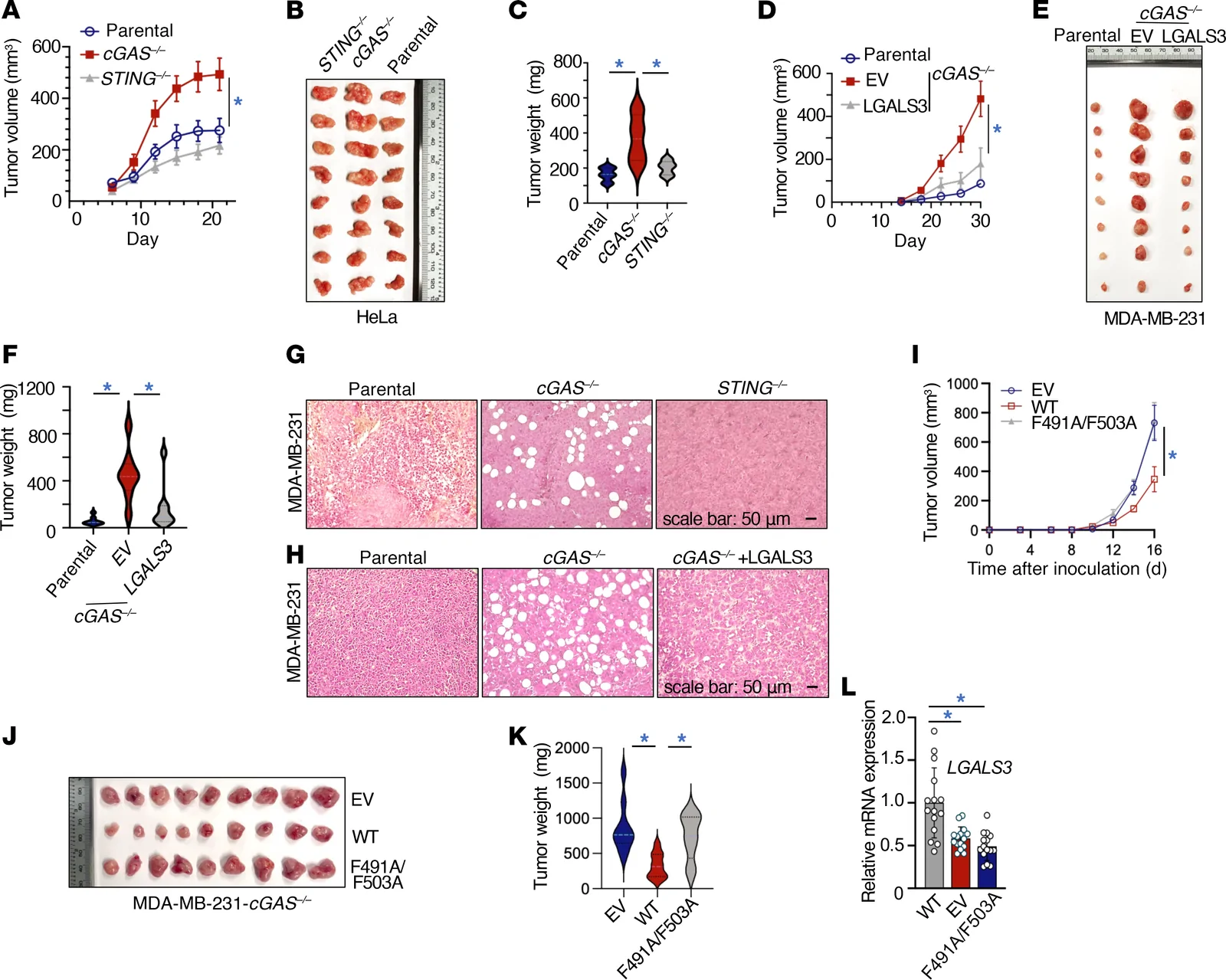
The transcriptional activity of cGAS regulates xenografted tumor growth. (**A** and **D**) Tumor volume measurements over time for xenografted cell lines. Error bars represent mean ± SD. *n* = 8. *P < 0.05 (1-way ANOVA test). (**B** and **E**) Images of isolated tumors from **A** and **D** with corresponding tumor weights shown in **C** and **F**. Error bars represent mean ± SD. *n* = 8. *P < 0.05 (1-way ANOVA test). (**G** and **H**) Representative H&E staining images of fixed tumor sections from the indicated xenografted tumors. (**I**) Tumor volume measurements over time for xenografted cell lines. Error bars represent mean ± SD. *n* = 9. *P < 0.05 (1-way ANOVA test). (**J**) Images of isolated tumors from **I** with corresponding tumor weights shown in **K**. Error bars represent mean ± SD. *n* = 9. *P < 0.05 (1-way ANOVA test). (**L**) RT-PCR analysis of *LGALS3* mRNA levels in indicated collected tumors from **J**. Error bars represent mean ± SD. *n* = 9. *P < 0.05 (1-way ANOVA test).

**Figure 12:**
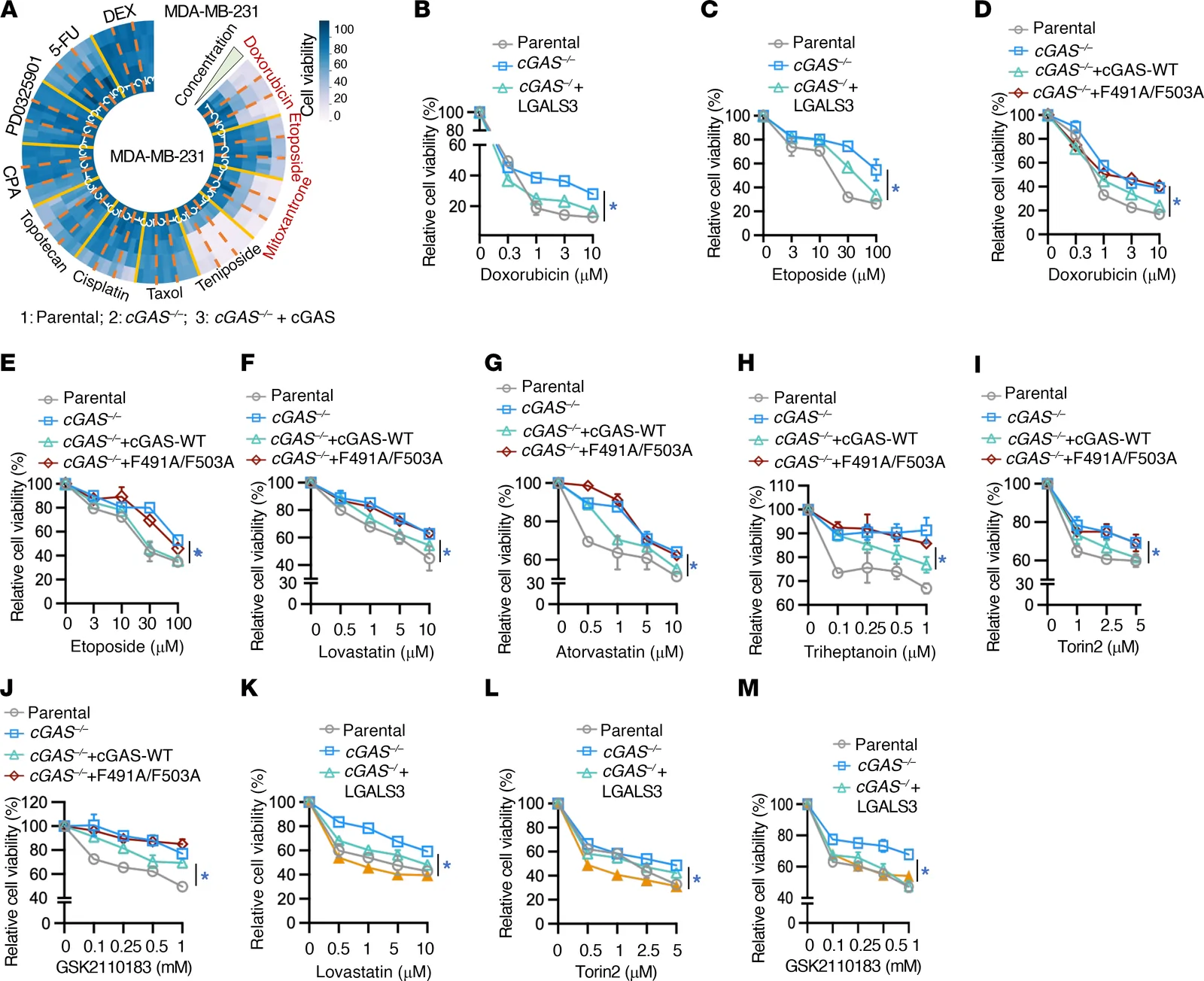
The transcriptional activity of cGAS influences cellular responses to therapies. (**A**) Heatmap displaying cell responses to the indicated treatments. (**B**–**M**) MTT assays using the indicated MDA-MB-231 cells treated for 3 days with indicated agents at the indicated doses. Error bars represent mean ± SD. *n* = 3. *P < 0.05 (1-way ANOVA test).

**Figure 13:**
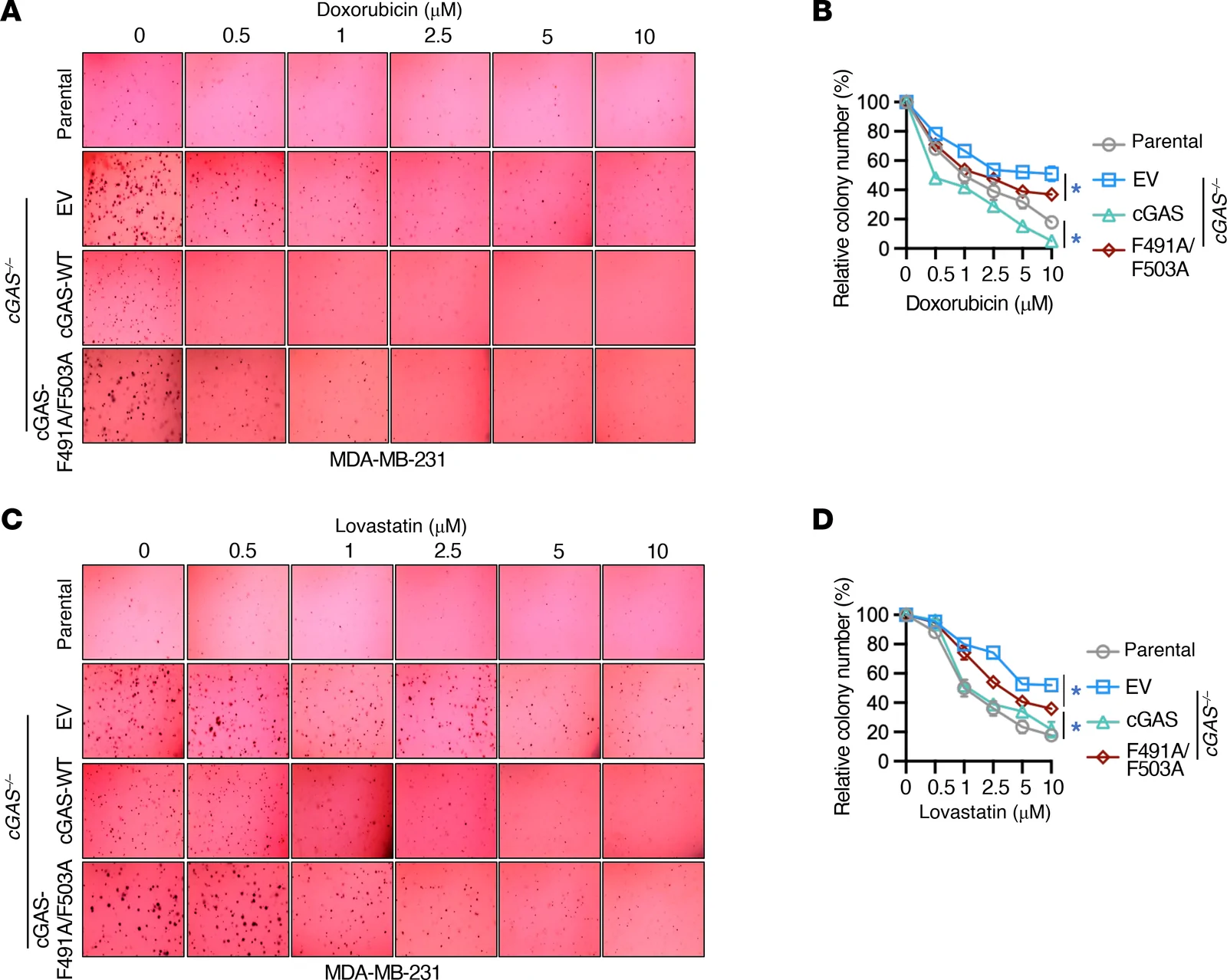
Transcriptional activity of cGAS influences cellular responses to therapy in 3D soft agar assays. (**A** and **C**) Representative 3D soft agar assay images of indicated MDA-MB-231 cells treated with indicated doses of doxorubicin (**A**) or lovastatin (**C**) for 3 weeks and quantified in **B** and **D**. Compounds were refreshed every 3 days. Error bars represent mean ± SD. *n* = 3. *P < 0.05 (1-way ANOVA test).

## Version 1 11/02/2026

Electronic publication

## References

[1] Zhang X (2013). Cyclic GMP-AMP containing mixed phosphodiester linkages is an endogenous high-affinity ligand for STING. Mol Cell.

[2] Sun L (2013). Cyclic GMP-AMP synthase is a cytosolic DNA sensor that activates the type I interferon pathway. Science.

[3] Corrales L (2016). The host STING pathway at the interface of cancer and immunity. J Clin Invest.

[4] Barber GN (2015). STING: infection, inflammation and cancer. Nat Rev Immunol.

[5] Vincent J (2017). Small molecule inhibition of cGAS reduces interferon expression in primary macrophages from autoimmune mice. Nat Commun.

[6] Wang H (2017). cGAS is essential for the antitumor effect of immune checkpoint blockade. Proc Natl Acad Sci U S A.

[7] Chen H (2020). cGAS suppresses genomic instability as a decelerator of replication forks. Sci Adv.

[8] Gentili M (2019). The N-terminal domain of cGAS determines preferential association with centromeric DNA and innate immune activation in the nucleus. Cell Rep.

[9] Volkman HE (2019). Tight nuclear tethering of cGAS is essential for preventing autoreactivity. Elife.

[10] Li T (2021). Phosphorylation and chromatin tethering prevent cGAS activation during mitosis. Science.

[11] Lv G (2024). mTORC2-driven chromatin cGAS mediates chemoresistance through epigenetic reprogramming in colorectal cancer. Nat Cell Biol.

[12] Chen W (2024). RSK2-mediated cGAS phosphorylation induces cGAS chromatin-incorporation-mediated cell transformation and cancer cell colony growth. Cell Death Discov.

[13] Guey B (2020). BAF restricts cGAS on nuclear DNA to prevent innate immune activation. Science.

[14] Xu P (2024). The CRL5-SPSB3 ubiquitin ligase targets nuclear cGAS for degradation. Nature.

[15] Zhao B (2020). The molecular basis of tight nuclear tethering and inactivation of cGAS. Nature.

[16] Pathare GR (2020). Structural mechanism of cGAS inhibition by the nucleosome. Nature.

[17] Michalski S (2020). Structural basis for sequestration and autoinhibition of cGAS by chromatin. Nature.

[18] Kujirai T (2020). Structural basis for the inhibition of cGAS by nucleosomes. Science.

[19] Cao D (2020). Structural basis for nucleosome-mediated inhibition of cGAS activity. Cell Res.

[20] Boyer JA (2020). Structural basis of nucleosome-dependent cGAS inhibition. Science.

[21] Liu H (2018). Nuclear cGAS suppresses DNA repair and promotes tumorigenesis. Nature.

[22] Buske FA (2010). Assigning roles to DNA regulatory motifs using comparative genomics. Bioinformatics.

[23] Zhang M (2021). FOSL1 promotes metastasis of head and neck squamous cell carcinoma through super-enhancer-driven transcription program. Mol Ther.

[24] Liu J (2021). TF-PROTACs enable targeted degradation of transcription factors. J Am Chem Soc.

[25] Wang Z (2023). Telomere targeting chimera enables targeted destruction of telomeric repeat-binding factor proteins. J Am Chem Soc.

[26] Sadowski I (1988). GAL4-VP16 is an unusually potent transcriptional activator. Nature.

[27] Piskacek M (2021). The 9aaTAD activation domains in the yamanaka transcription factors Oct4, Sox2, Myc, and Klf4. Stem Cell Rev Rep.

[28] Jutz S (2016). Assessment of costimulation and coinhibition in a triple parameter T cell reporter line: Simultaneous measurement of NF-κB, NFAT and AP-1. J Immunol Methods.

[29] Tao J (2017). Nonspecific DNA Binding of cGAS N Terminus Promotes cGAS Activation. J Immunol.

[30] Lee A (2017). The N terminus of cGAS de-oligomerizes the cGAS:DNA complex and lifts the DNA size restriction of core-cGAS activity. FEBS Lett.

[31] Du M, Chen ZJ (2018). DNA-induced liquid phase condensation of cGAS activates innate immune signaling. Science.

[32] Waudby CA (2022). An intrinsic temporal order of c-JUN N-terminal phosphorylation regulates its activity by orchestrating co-factor recruitment. Nat Commun.

[33] Maxfield FR, Wustner D (2012). Analysis of cholesterol trafficking with fluorescent probes. Methods Cell Biol.

[34] Wang Y (2022). Acetyl-CoA Carboxylases and Diseases. Front Oncol.

[35] Friesen JA, Rodwell VW (2004). The 3-hydroxy-3-methylglutaryl coenzyme-A (HMG-CoA) reductases. Genome Biol.

[36] Zhuang L (2005). Cholesterol targeting alters lipid raft composition and cell survival in prostate cancer cells and xenografts. J Clin Invest.

[37] Xu J (2010). Cholesterol trafficking is required for mTOR activation in endothelial cells. Proc Natl Acad Sci U S A.

[38] Sanvee GM (2021). mTORC2 is an important target for simvastatin-associated toxicity in C2C12 cells and mouse skeletal muscle - Roles of Rap1 geranylgeranylation and mitochondrial dysfunction. Biochem Pharmacol.

[39] Wang Y (2022). Cholesterol-Lowering Intervention Decreases mTOR Complex 2 Signaling and Enhances Antitumor Immunity. Clin Cancer Res.

[40] Maehama T, Dixon JE (1998). The tumor suppressor, PTEN/MMAC1, dephosphorylates the lipid second messenger, phosphatidylinositol 3,4,5-trisphosphate. J Biol Chem.

[41] Liu P (2015). PtdIns(3,4,5)P3-Dependent Activation of the mTORC2 Kinase Complex. Cancer Discov.

[42] Fessler MB (2017). A New Frontier in Immunometabolism. Cholesterol in Lung Health and Disease. Ann Am Thorac Soc.

[44] Overmyer KA (2015). Impact of anesthesia and euthanasia on metabolomics of mammalian tissues: studies in a C57BL/6J mouse model. PLoS One.

[45] Abolhassani M (2009). Carbon dioxide inhalation causes pulmonary inflammation. Am J Physiol Lung Cell Mol Physiol.

[46] Phelan DE (2023). Hypercapnia alters mitochondrial gene expression and acylcarnitine production in monocytes. Immunol Cell Biol.

[47] Nardelli LM (2015). Effects of acute hypercapnia with and without acidosis on lung inflammation and apoptosis in experimental acute lung injury. Respir Physiol Neurobiol.

[48] Xu K (2014). mTOR signaling in tumorigenesis. Biochim Biophys Acta.

[49] Battaglioni S (2022). mTOR substrate phosphorylation in growth control. Cell.

[50] Manning BD, Toker A (2017). AKT/PKB Signaling: Navigating the Network. Cell.

[51] Dong C (2021). Activation of PI3K/AKT/mTOR pathway causes drug resistance in breast cancer. Front Pharmacol.

[52] Yu L (2022). Attacking the PI3K/Akt/mTOR signaling pathway for targeted therapeutic treatment in human cancer. Semin Cancer Biol.

[53] Obidiro O (2023). Triple negative breast cancer treatment options and limitations: future outlook. Pharmaceutics.

[54] Regalado Porras GO (2018). Chemotherapy and molecular therapy in cervical cancer. Rep Pract Oncol Radiother.

[55] Qiu S (2023). Mitochondria-localized cGAS suppresses ferroptosis to promote cancer progression. Cell Res.

[56] Ma D (2024). cGAS suppresses hepatocellular carcinoma independent of its cGAMP synthase activity. Cell Death Differ.

[57] Chen C, Xu P (2023). Cellular functions of cGAS-STING signaling. Trends Cell Biol.

[58] Wang F (2024). cGAS regulates metabolic reprogramming independently of STING pathway in colorectal cancer. Exp Cell Res.

[59] Cai Z (2024). cGAS suppresses β-cell proliferation by a STING-independent but CEBPβ-dependent mechanism. Metabolism.

[60] Ito A (2016). Cholesterol accumulation in CD11c(+) immune cells is a causal and targetable factor in autoimmune disease. Immunity.

[61] Diaz-Alvarez L, Ortega E (2017). The many roles of galectin-3, a multifaceted molecule, in innate immune responses against pathogens. Mediators Inflamm.

[62] Farhad M (2018). The role of Galectin-3 in modulating tumor growth and immunosuppression within the tumor microenvironment. Oncoimmunology.

[63] Stojanovic BS (2023). The pivotal role of Galectin-3 in viral infection: a multifaceted player in host-pathogen interactions. Int J Mol Sci.

[64] Deng Y (2024). 2′3′-cGAMP interactome identifies 2’3’-cGAMP/Rab18/FosB signaling in cell migration control independent of innate immunity. Sci Adv.

[65] Zhu Z (2023). STING suppresses mitochondrial VDAC2 to govern RCC growth independent of innate immunity. Adv Sci (Weinh).

[66] Jiang Y (2019). MERTK mediated novel site Akt phosphorylation alleviates SAV1 suppression. Nat Commun.

[67] Su S (2021). SPOP and OTUD7A Control EWS-FLI1 Protein Stability to Govern Ewing Sarcoma Growth. Adv Sci (Weinh).

[68] Liu P (2014). Cell-cycle-regulated activation of Akt kinase by phosphorylation at its carboxyl terminus. Nature.

[69] Yu L (2024). Co-targeting JAK1/STAT6/GAS6/TAM signaling improves chemotherapy efficacy in Ewing sarcoma. Nat Commun.

[70] Abramson J (2024). Accurate structure prediction of biomolecular interactions with AlphaFold 3. Nature.

[71] Livraghi A (2009). Airway and lung pathology due to mucosal surface dehydration in β-epithelial Na^+^ channel-overexpressing mice: role of TNF-α and IL-4Rα signaling, influence of neonatal development, and limited efficacy of glucocorticoid treatment. J Immunol.

[72] Livraghi-Butrico A (2012). Genetically determined heterogeneity of lung disease in a mouse model of airway mucus obstruction. Physiol Genomics.

[73] Knowles MR (1997). Ion composition of airway surface liquid of patients with cystic fibrosis as compared with normal and disease-control subjects. J Clin Invest.

