# Supplementary material for: Nuclear cGAS governs transcription to control lipid metabolism, tumor growth, and therapy response
Source: J Clin Invest. 2026 Nov 2;136(21):e205579. doi: 10.1172/JCI205579 (PMC13722394; doi:10.1172/JCI205579)
Supplement: Supplementary file 1 [file jci-136-205579-s015.pdf]

Supplemental information

Nuclear cGAS governs transcription to control lipid metabolism, tumor growth and therapy response

Yu Deng, Matthew J. Niederhuber, Zhichuan Zhu, Austin J. Hepperla, Le Yu, Quentin Hahn, Joshua Hartsell, Tooba Rashid, Piotr A. Mieczkowski, Hemant Kelkar, Jeremy M. Simon, Yanqiong Zhang, Alessandra Livraghi-Butrico, Meredith J. Zeller, Joseph G.C. Kennedy, Jazmin F. DePriest, Jiong Li, Yan Zhang, Xing Qiu, H. Ümit Kaniskan, Jian Jin, Corbin D. Jones, Laura E. Herring, Douglas H. Phanstiel, Xiaojing Liu, Jesse R. Raab, Brian D. Strahl, and Pengda Liu

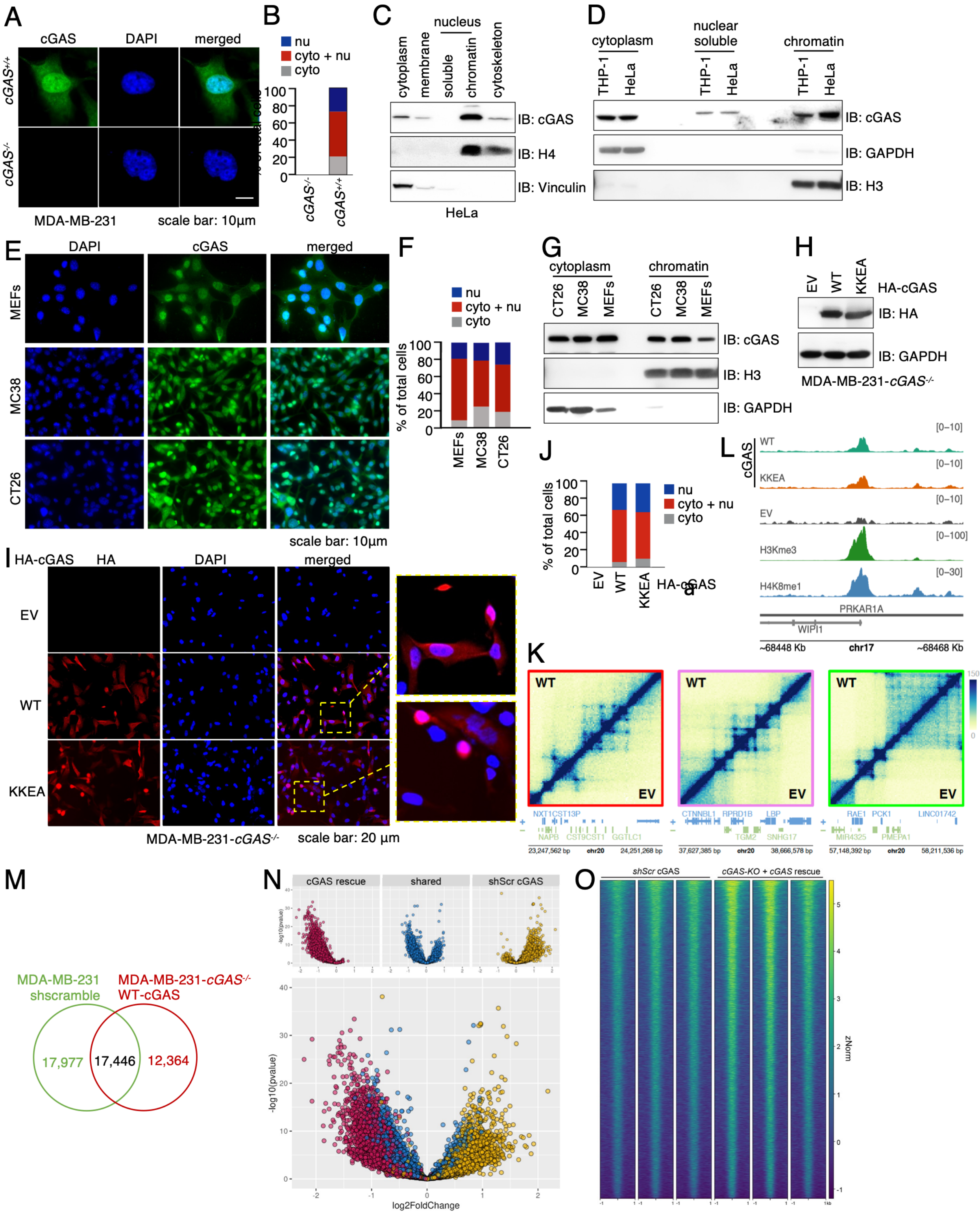
A
cGAS
DAPI
merged
cGAS+/+
cGAS-/-
MDA-MB-231
scale bar: 10µm
B
nu
cyto + nu
cyto
cGAS-/-
cGAS+/+
C
cytoplasm
membrane
nucleus
soluble
chromatin
cytoskeleton
IB: cGAS
IB: H4
IB: Vinculin
HeLa
D
cytoplasm
nuclear soluble
chromatin
THP-1
HeLa
IB: cGAS
IB: GAPDH
IB: H3
E
DAPI
cGAS
merged
MEFs
MC38
CT26
scale bar: 10µm
F
nu
cyto + nu
cyto
% of total cells
MEFs
MC38
CT26
G
cytoplasm
chromatin
CT26
MC38
MEFs
IB: cGAS
IB: H3
IB: GAPDH
H
EV
WT
KKEA
HA-cGAS
IB: HA
IB: GAPDH
MDA-MB-231-cGAS-/-
I
HA-cGAS
HA
DAPI
merged
EV
WT
KKEA
MDA-MB-231-cGAS-/-
scale bar: 20 µm
J
% of total cells
nu
cyto + nu
cyto
EV
WT
KKEA
HA-cGAS
K
WT
EV
L
cGAS
WT
KKEA
EV
H3Kme3
H4K8me1
[0–10]
[0–100]
[0–30]
PRKAR1A
WIPI1
~68448 Kb
chr17
~68468 Kb
M
MDA-MB-231 shscramble
MDA-MB-231-cGAS-/- WT-cGAS
17,977
17,446
12,364
N
cGAS rescue
shared
shScr cGAS
-log10(pvalue)
log2FoldChange
O
shScr cGAS
cGAS-KO + cGAS rescue
zNorm

Figure S1. Nuclear cGAS regulates transcription without altering chromatin architecture.
(A, B) Representative confocal IF images of indicated MDA-MB-231 cells. Cellular localization of cGAS is quantified in (B). nu: nuclear; cyto: cytoplasm.
(C, D, G) IB analysis of indicated cellular fractionations from HeLa (C), THP1 (D) and indicated mouse cells (G).
(E, F) Representative confocal IF images of indicated cells. Cellular localization of cGAS is quantified in (F). nu: nuclear; cyto: cytoplasm.
(H) IB analysis of WCL from indicated MDA-MB-231 cells.
(I, J) Representative confocal IF images of indicated MDA-MB-231 cells. Cellular localization of cGAS is quantified in (J). nu: nuclear; cyto: cytoplasm.
(K) Chromatin interaction heatmaps of Micro-C data in indicated genome regions.
(L) Representative genome browser views for indicated cGAS *CUT&RUN* peaks at the *PRKAR1A* locus.
(M) A venn diagram showing identified endogenous and reconstituted cGAS *CUT&RUN* peaks in MDA-MB-231 cells.
(N) A volcano plot from endogenous and exogenous cGAS *CUT&RUN* analysis showing overlapping and non-overlapping hits.
(O) A heatmap for all indicated *CUT&RUN* peaks spanning -1 to 1 kb regions in indicated MDA-MB-231 cells.

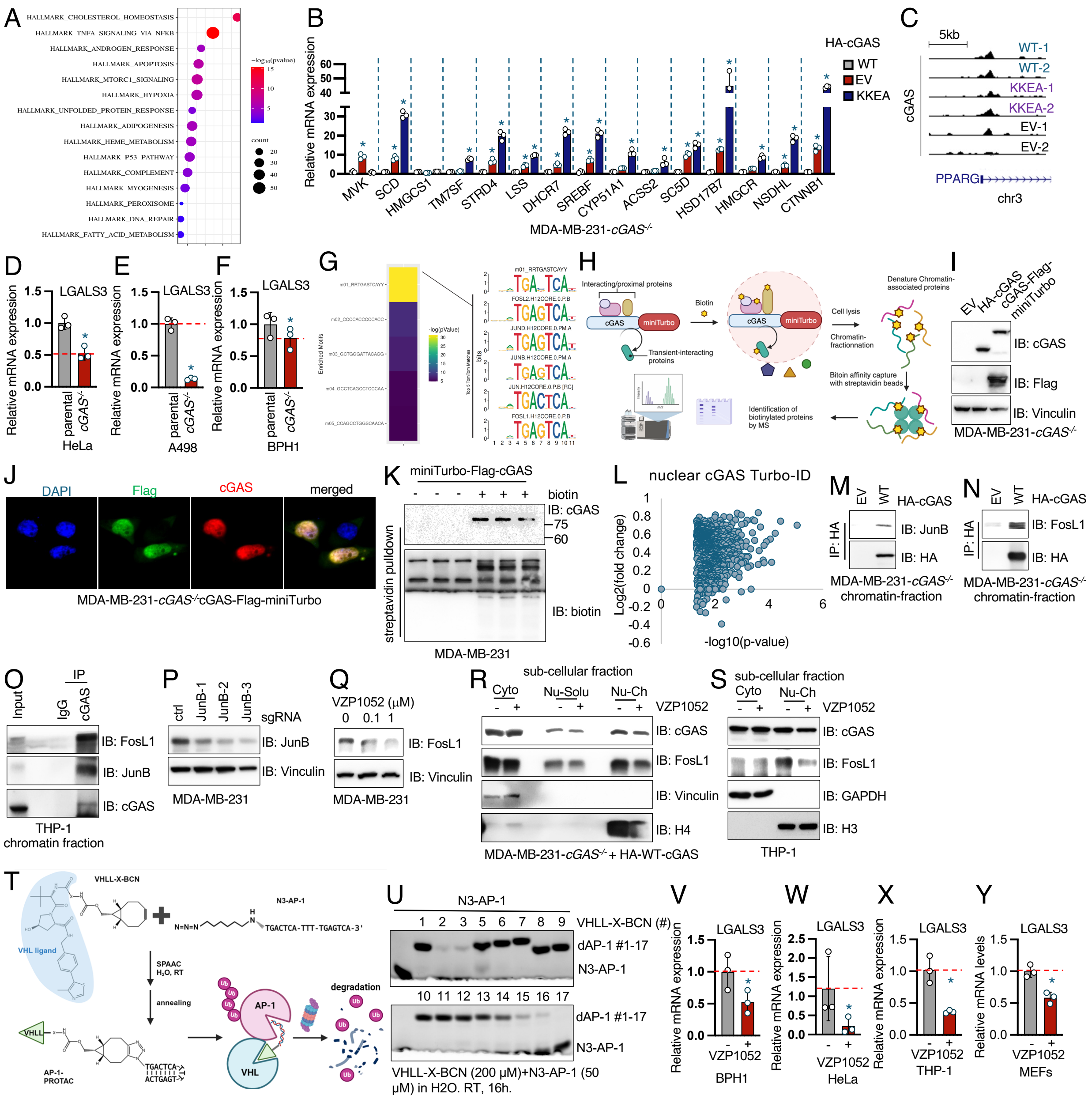

Figure S2. cGAS associates with AP-1 transcription factors to control LGALS3 transcription.

(A) Pathway analyses using GO and KEGG, focusing on cellular processes. Fold changes are represented by the size of the dots, and statistical significance is indicated by warm/cold colors as shown.

(B) RT-PCR analysis of mRNA expression of indicated genes in indicated MDA-MB-231 cells. Error bars were calculated as mean+/-SD, n=3. *p<0.05 (one-way ANOVA test).

(C) Representative genome browser views for indicated cGAS *CUT&RUN* peaks at the *PPARG* locus.

(D-F) RT-PCR analysis of LGALS3 mRNA expression in indicated cells. Error bars were calculated as mean+/-SD, n=3. *p < 0.05 (one-way ANOVA test).

(G) *De novo* motif analysis with STREME revealing a strong enrichment of the AP-1 motif

from cGAS *CUT&RUN* peaks in MDA-MB-231 cells.

(H) A schematic illustration of the nuclear cGAS Turbo-ID experimental procedure. cGAS is tagged with a mini-Turbo tag and stably expressed in MDA-MB-231-*cGAS*$^{-/-}$ cells via lentiviral infection. For detailed methods, please refer to the methods section.

(I) IB analysis of WCL from indicated MDA-MB-231 cells.

(J) Representative confocal IF images showing the cellular localization of cGAS-mini-Turbo. Scale bar=10 μm.

(K) IB analysis of streptavidin beads pulldowns from MDA-MB-231 cells with or without biotin addition for 30 min.

(L) A volcano plot indicating significant protein hits associated cGAS-Turbo in MDA-MB-231 cells.

(M) IB analysis of HA-IPs and WCL from chromatin fractions of indicated MDA-MB-231 cells.

(N) IB analysis of HA-IPs and WCL from chromatin fractions of indicated MDA-MB-231 cells.

(O) IB analysis of endogenous cGAS-IPs and WCL from chromatin fractions of THP1 cells.

(P) IB analysis of WCL from indicated MDA-MB-231 cells.

(Q) IB analysis of WCL from MDA-MB-231 cells treated with indicated doses of VZP1052 for 24 hrs prior to cell collection.

(R) IB analysis of indicated cellular fractions from MDA-MB-231 cells treated with or without VZP1052 for 24 hrs prior to cell collection.

(S) IB analysis of indicated cellular fractions from THP1 cells treated with or without VZP1052 for 24 hrs prior to cell collection.

(T) A schematic diagram illustrating the preparation of AP-1 TF-PROTACs via the SPAAC (strain-promoted azide-alkyne cycloaddition) reaction using a BCN-modified VHL ligand and N3-modified AP-1 recognizing DNA oligo (N3-AP-1), and the proposed mechanism of proteasomal degradation of AP-1 transcription factors upon AP-1 TF-PROTACs transfection.

(U) A representative 20% DNA-PAGE gel showing the click efficiency of the N3-AP-1 oligo to indicated number of VHL ligands. The details for each numbered VHL ligands could be found here (1).

(V) RT-PCR analysis of LGALS3 mRNA expression in BPH1 cells treated with or without VZP1052 for 24 hrs prior to cell collection. Error bars were calculated as mean+/-SD, n=3. *$p < 0.05$ (one-way ANOVA test).

(W) RT-PCR analysis of LGALS3 mRNA expression in HeLa cells treated with or without VZP1052 for 24 hrs prior to cell collection. Error bars were calculated as mean+/-SD, n=3. *$p < 0.05$ (one-way ANOVA test).

(X) RT-PCR analysis of LGALS3 mRNA expression in indicated THP1 cells. Error bars were calculated as mean+/-SD, n=3. *$p < 0.05$ (one-way ANOVA test).

(Y) RT-PCR analysis of LGALS3 mRNA expression in indicated MEF cells. Error bars were calculated as mean+/-SD, n=3. *$p < 0.05$ (one-way ANOVA test).

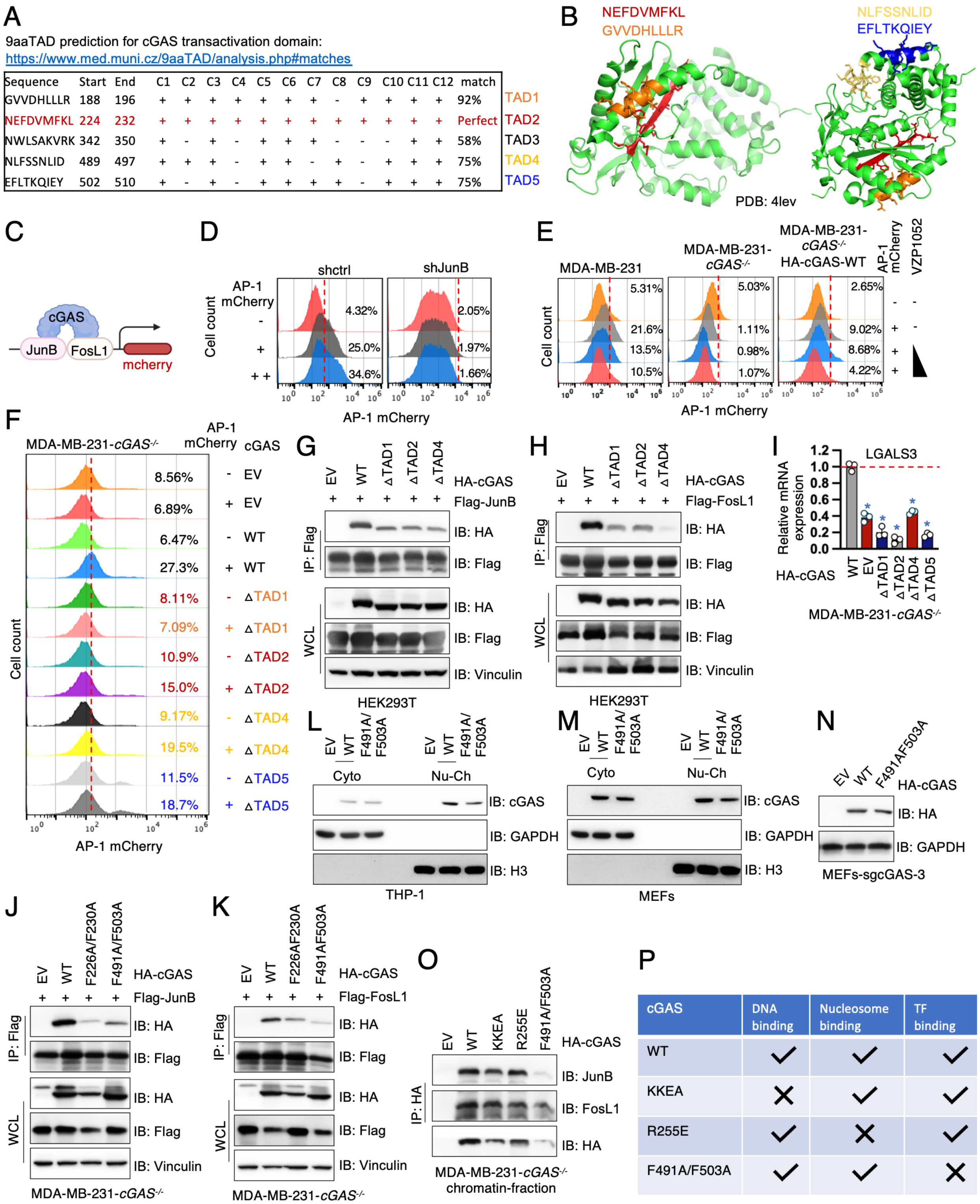

Figure S3. cGAS associates with JunB and FosL1 via TAD domain(s).
(A) An illustration of a prediction result using 9aaTAD for potential cGAS TAD domains.
(B) Illustration of localization of predicted TADs in available cGAS structure (PDB: 4lev).
(C) A schematic illustration of AP-1 mcherry reporter working mechanism. When cGAS

binds JunB and/or FosL1, it drives the transcription of mcherry reporter.
(D) A representative FACS assay showing depletion of endogenous JunB reduces AP-1 reporter activity in MDA-MB-231 cells.
(E) Representative FACS assays showing degradation of FosL1 by its PROTAC VZP1052 reduces AP-1 reporter activity in WT-cGAS but not cGAS-deleted MDA-MB-231 cells.
(F) Representative FACS assays showing effects of AP-1 reporter activity in MDA-MB-231 cells expressing indicated cGAS TAD-deletion mutants.
(G) IB analysis of Flag-IPs and WCL from HEK293T cells transfected with indicated DNA constructs.
(H) IB analysis of Flag-IPs and WCL from HEK293T cells transfected with indicated DNA constructs.
(I) RT-PCR analysis of LGALS3 mRNA expression in indicated MDA-MB-231 cells. Error bars were calculated as mean+/-SD, n=3. *$p < 0.05$ (one-way ANOVA test).
(J) IB analysis of Flag-IPs and WCL from indicated MDA-MB-231 cells.
(K) IB analysis of Flag-IPs and WCL from indicated MDA-MB-231 cells.
(L, M) IB analysis of indicated cellular fractions from THP1 cells (L) or MEFs (M) expressing indicated cGAS mutants.
(N) IB analysis of indicated MEF cells.
(O) IB analysis of HA-IPs from chromatin fractions using indicated MDA-MB-231 cells.
(P) A table summarizing cGAS point mutants in dissecting indicated cGAS function.

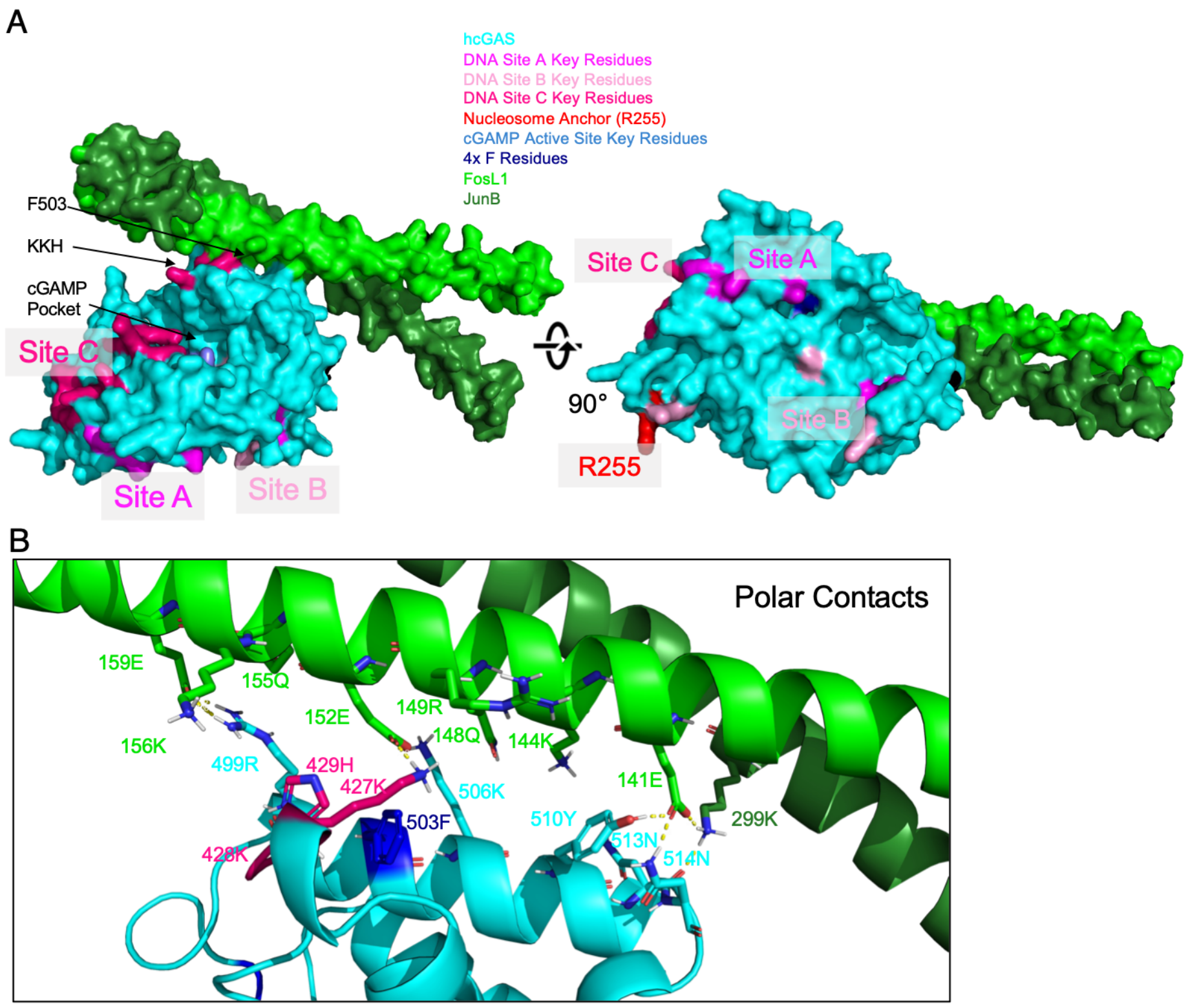

Figure S4. A structure simulation of cGAS binding to AP-1 factors.

(A) A refined structural model of the cGAS/FosL1/JunB heterotrimer was generated for molecular dynamics (MD) simulations using cGAS (UniProt: Q8N884), FosL1 (UniProt: P15407), and JunB (UniProt: P17275). Colors denote the indicated proteins and cGAS structural motifs.

(B) Refined structural simulation illustrating the molecular interface between cGAS and the JunB/FosL1 heterodimer.

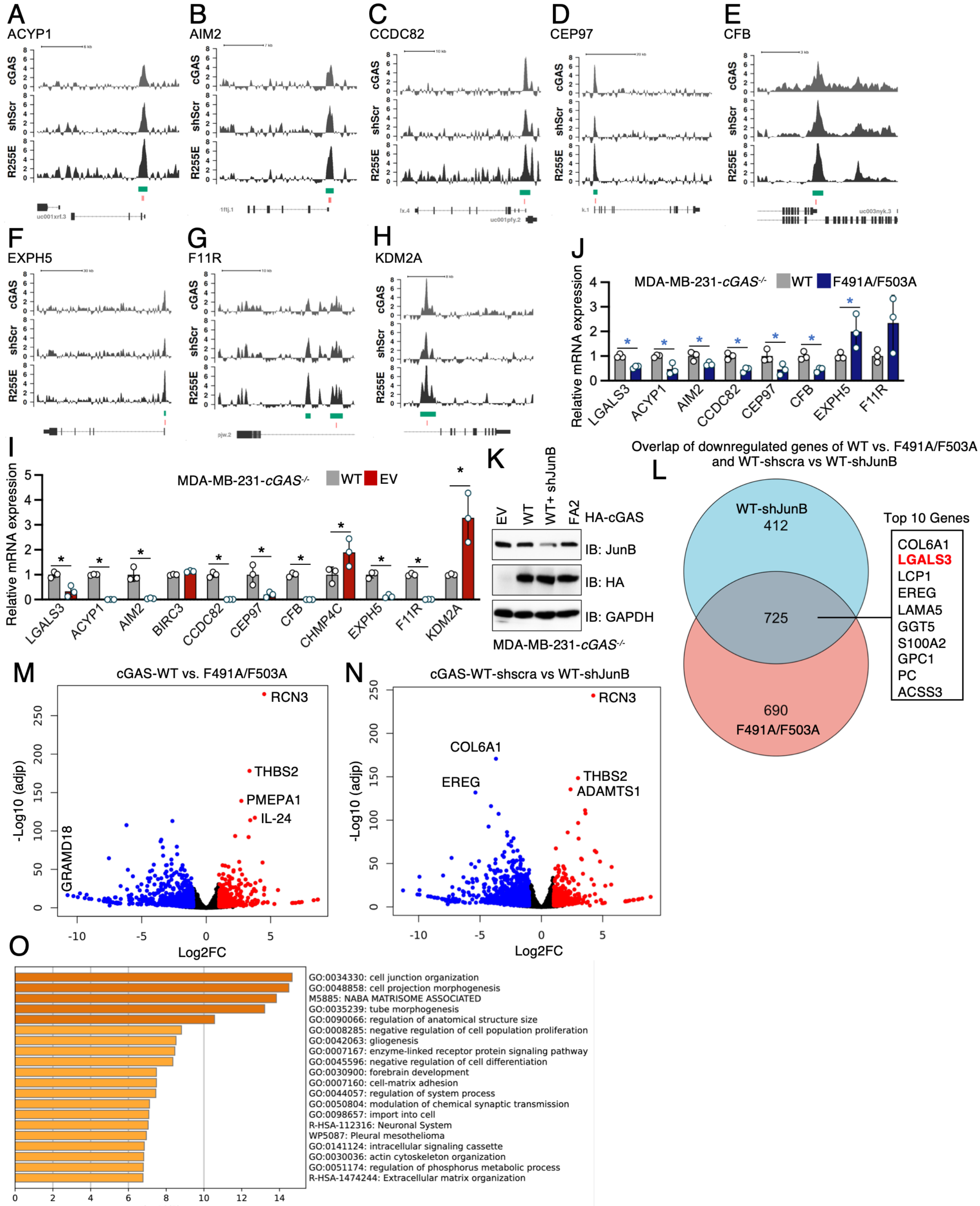

A
ACYP1
B
AIM2
C
CCDC82
D
CEP97
E
CFB
F
EXPH5
G
F11R
H
KDM2A
cGAS
shScr
R255E
J
MDA-MB-231-cGAS-/-
WT
F491A/F503A
Relative mRNA expression
LGALS3
ACYP1
AIM2
CCDC82
CEP97
CFB
EXPH5
F11R
I
MDA-MB-231-cGAS-/-
WT
EV
Relative mRNA expression
LGALS3
ACYP1
AIM2
BIRC3
CCDC82
CEP97
CFB
CHMP4C
EXPH5
F11R
KDM2A
K
EV
WT
WT+ shJunB
FA2
HA-cGAS
IB: JunB
IB: HA
IB: GAPDH
MDA-MB-231-cGAS-/-
Overlap of downregulated genes of WT vs. F491A/F503A and WT-shscra vs WT-shJunB
L
WT-shJunB
412
725
690
F491A/F503A
Top 10 Genes
COL6A1
LGALS3
LCP1
EREG
LAMA5
GGT5
S100A2
GPC1
PC
ACSS3
M
cGAS-WT vs. F491A/F503A
RCN3
THBS2
PMEPA1
IL-24
GRAMD18
-Log10 (adjp)
Log2FC
N
cGAS-WT-shscra vs WT-shJunB
RCN3
COL6A1
EREG
THBS2
ADAMTS1
-Log10 (adjp)
Log2FC
O
GO:0034330: cell junction organization
GO:0048858: cell projection morphogenesis
M5885: NABA MATRISOME ASSOCIATED
GO:0035239: tube morphogenesis
GO:0090066: regulation of anatomical structure size
GO:0008285: negative regulation of cell population proliferation
GO:0042063: gliogenesis
GO:0007167: enzyme-linked receptor protein signaling pathway
GO:0045596: negative regulation of cell differentiation
GO:0030900: forebrain development
GO:0007160: cell-matrix adhesion
GO:0044057: regulation of system process
GO:0050804: modulation of chemical synaptic transmission
GO:0098657: import into cell
R-HSA-112316: Neuronal System
WP5087: Pleural mesothelioma
GO:0141124: intracellular signaling cassette
GO:0030036: actin cytoskeleton organization
GO:0051174: regulation of phosphorus metabolic process
R-HSA-1474244: Extracellular matrix organization
-log10(P)

Figure S5. cGAS cooperates with AP-1 factors to modulate transcriptional programs.

(A-H) Representative genome browser views for cGAS *CUT&RUN* peaks at indicated promoters from indicated MDA-MB-231 cells.

(I, J) RT-PCR analysis of LGALS3 mRNA expression in indicated MDA-MB-231 cells. Error bars were calculated as mean+/-SD, n=3. *$p < 0.05$ (one-way ANOVA test).

(K) IB analysis of WCL from indicated MDA-MB-231 cells.

(L) A venn diagram showing identified transcriptomic changes in indicated MDA-MB-231 cells.

(M, N) Representative volcano plots showing changes of transcriptional targets in indicated MDA-MB-231 cells.

(O) A GO analysis of functional enrichment for overlapped transcriptional targets governed by both cGAS and JunB in MDA-MB-231 cells.

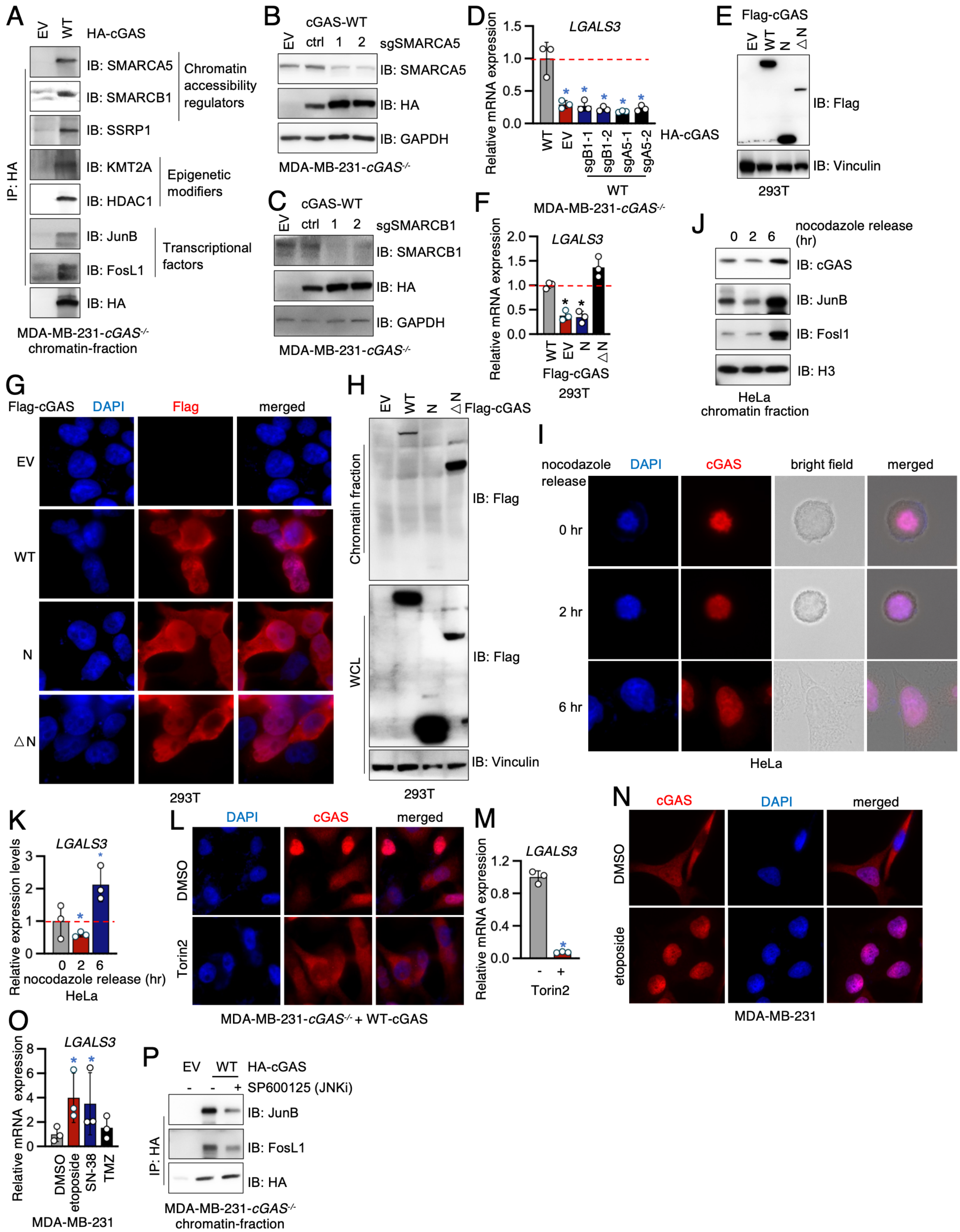
A
EV
WT
HA-cGAS
IB: SMARCA5
IB: SMARCB1
Chromatin accessibility regulators
IB: SSRP1
IB: KMT2A
IB: HDAC1
Epigenetic modifiers
IB: JunB
IB: FosL1
Transcriptional factors
IB: HA
IP: HA
MDA-MB-231-cGAS-/-
chromatin-fraction
B
cGAS-WT
EV
ctrl
1
2
sgSMARCA5
IB: SMARCA5
IB: HA
IB: GAPDH
MDA-MB-231-cGAS-/-
C
cGAS-WT
EV
ctrl
1
2
sgSMARCB1
IB: SMARCB1
IB: HA
IB: GAPDH
MDA-MB-231-cGAS-/-
D
LGALS3
Relative mRNA expression
1.5
1.0
0.5
0.0
WT
EV
sgB1-1
sgB1-2
sgA5-1
sgA5-2
HA-cGAS
WT
MDA-MB-231-cGAS-/-
E
Flag-cGAS
EV
WT
N
△N
IB: Flag
IB: Vinculin
293T
F
LGALS3
Relative mRNA expression
2.0
1.5
1.0
0.5
0.0
WT
EV
N
△N
Flag-cGAS
293T
J
0
2
6
nocodazole release (hr)
IB: cGAS
IB: JunB
IB: Fosl1
IB: H3
HeLa
chromatin fraction
G
Flag-cGAS
DAPI
Flag
merged
EV
WT
N
△N
293T
H
EV
WT
N
△N
Flag-cGAS
Chromatin fraction
IB: Flag
WCL
IB: Flag
IB: Vinculin
293T
I
nocodazole release
DAPI
cGAS
bright field
merged
0 hr
2 hr
6 hr
HeLa
K
LGALS3
Relative expression levels
3
2
1
0
0
2
6
nocodazole release (hr)
HeLa
L
DAPI
cGAS
merged
DMSO
Torin2
MDA-MB-231-cGAS-/- + WT-cGAS
M
LGALS3
Relative mRNA expression
1.2
0.8
0.4
0.0
-
+
Torin2
N
cGAS
DAPI
merged
DMSO
etoposide
MDA-MB-231
O
LGALS3
Relative mRNA expression
8
6
4
2
0
DMSO
etoposide
SN-38
TMZ
MDA-MB-231
P
EV
WT
HA-cGAS
-
-
+
SP600125 (JNKi)
IB: JunB
IB: FosL1
IB: HA
IP: HA
MDA-MB-231-cGAS-/-
chromatin-fraction

Figure S6. cGAS forms a transcriptional regulatory complex to control gene transcription.

(A) IB analysis of HA-cGAS IPs from chromatin fractions in indicated MDA-MB-231 cells.
(B, C) IB analysis of WCL from indicated MDA-MB-231 cells.
(D, F) RT-PCR analysis of LGALS3 mRNA expression in indicated MDA-MB-231 cells. Error bars were calculated as mean+/-SD, n=3. **p* < 0.05 (one-way ANOVA test).
(E) IB analysis of WCL from HEK293T cells transfected with indicated cGAS constructs.
(G) Representative IF images of HEK293T cells transfected with indicated cGAS constructs.
(H) IB analysis of chromatin fractions and WCL from HEK293T cells transfected with indicated cGAS constructs.
(I) Representative IF images of HeLa cells transfected with indicated cGAS constructs and released from nocodazole arrest for indicated time periods.
(J) IB analysis of WCL from HeLa cells transfected with indicated cGAS constructs and released from nocodazole arrest for indicated time periods.
(K) RT-PCR analysis of LGALS3 mRNA expression in HeLa cells transfected with indicated cGAS constructs and released from nocodazole arrest for indicated time periods. Error bars were calculated as mean+/-SD, n=3. **p* < 0.05 (one-way ANOVA test).
(L) Representative IF images of indicated MDA-MB-231 cells treated with Torin 2 (100 nM) overnight.
(M) RT-PCR analysis of LGALS3 mRNA expression in indicated MDA-MB-231 cells treated with Torin 2 (100 nM) overnight. Error bars were calculated as mean+/-SD, n=3. **p* < 0.05 (one-way ANOVA test).
(N) Representative IF images of MDA-MB-231 cells treated with etoposide (10 μM overnight).
(O) RT-PCR analysis of LGALS3 mRNA expression in MDA-MB-231 cells treated with etoposide (10 μM), SN-38 (100 nM) or TMZ (100 μM). Error bars were calculated as mean+/-SD, n=3. **p* < 0.05 (one-way ANOVA test).
(P) IB analysis of HA-IP from chromatin fractions in indicated MDA-MB-231 cells. Where indicated, cells were treated with JNKi (SP600125) for 24 hrs (1 μM) before cell collection.

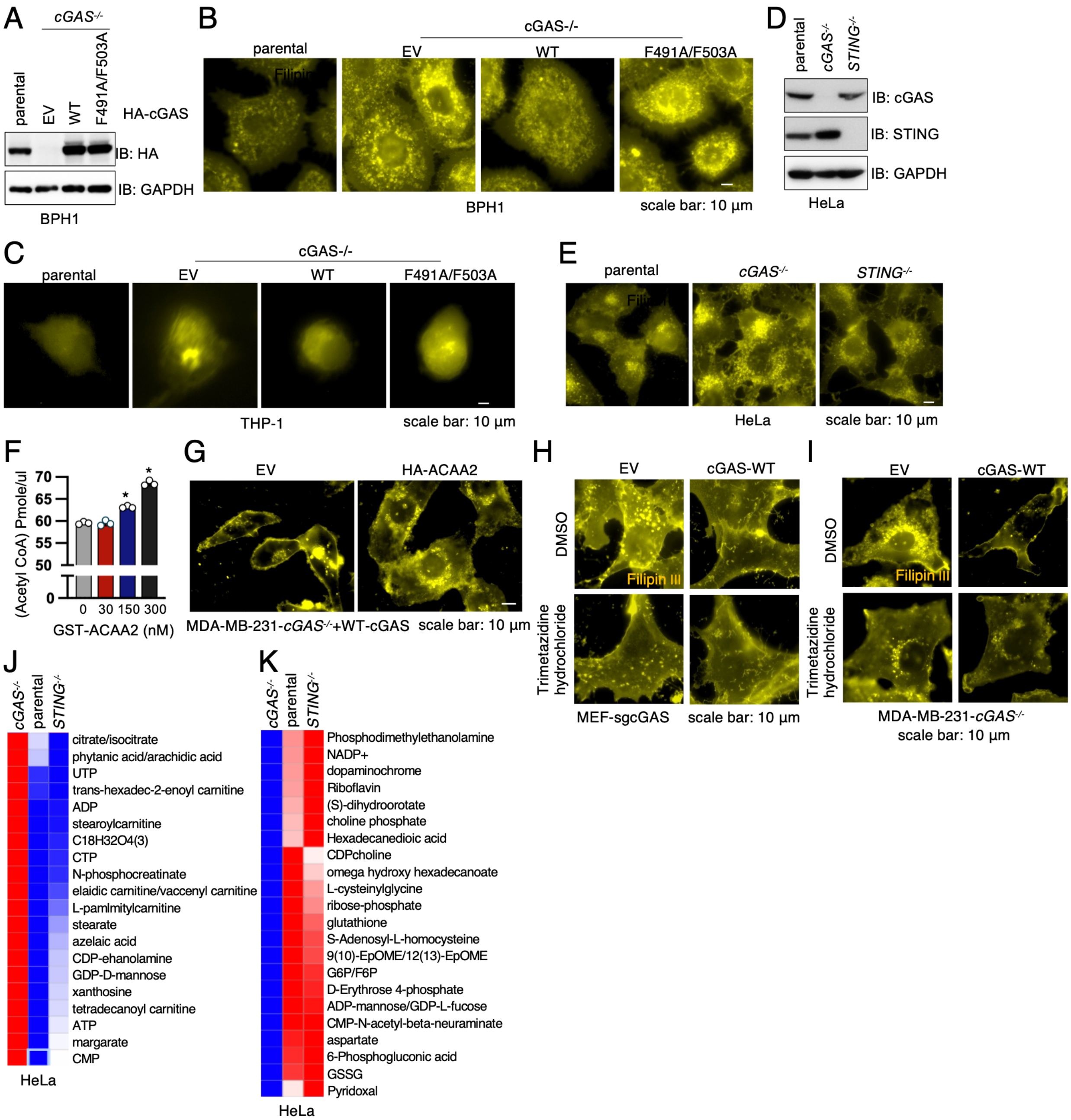

Figure S7. cGAS suppresses ACAA2 activity, leading to reduced cellular cholesterol levels.

(A, D) IB analysis of WCL from indicated cells.

(B, C, E, G, H, I) Representative IF images of Filippin III staining of free cholesterol in indicated cells. Where indicated in H and I, cells were treated with 100 μM trimetazidine hydrochloride for 12 hrs.

(F) An acetyl-CoA ELISA assay measuring activity of bacterially purified GST-ACAA2 activity in *in vitro* ACAA2 activity assays.

(J, K) A heatmap quantifying indicated metabolites in indicated HeLa cells.

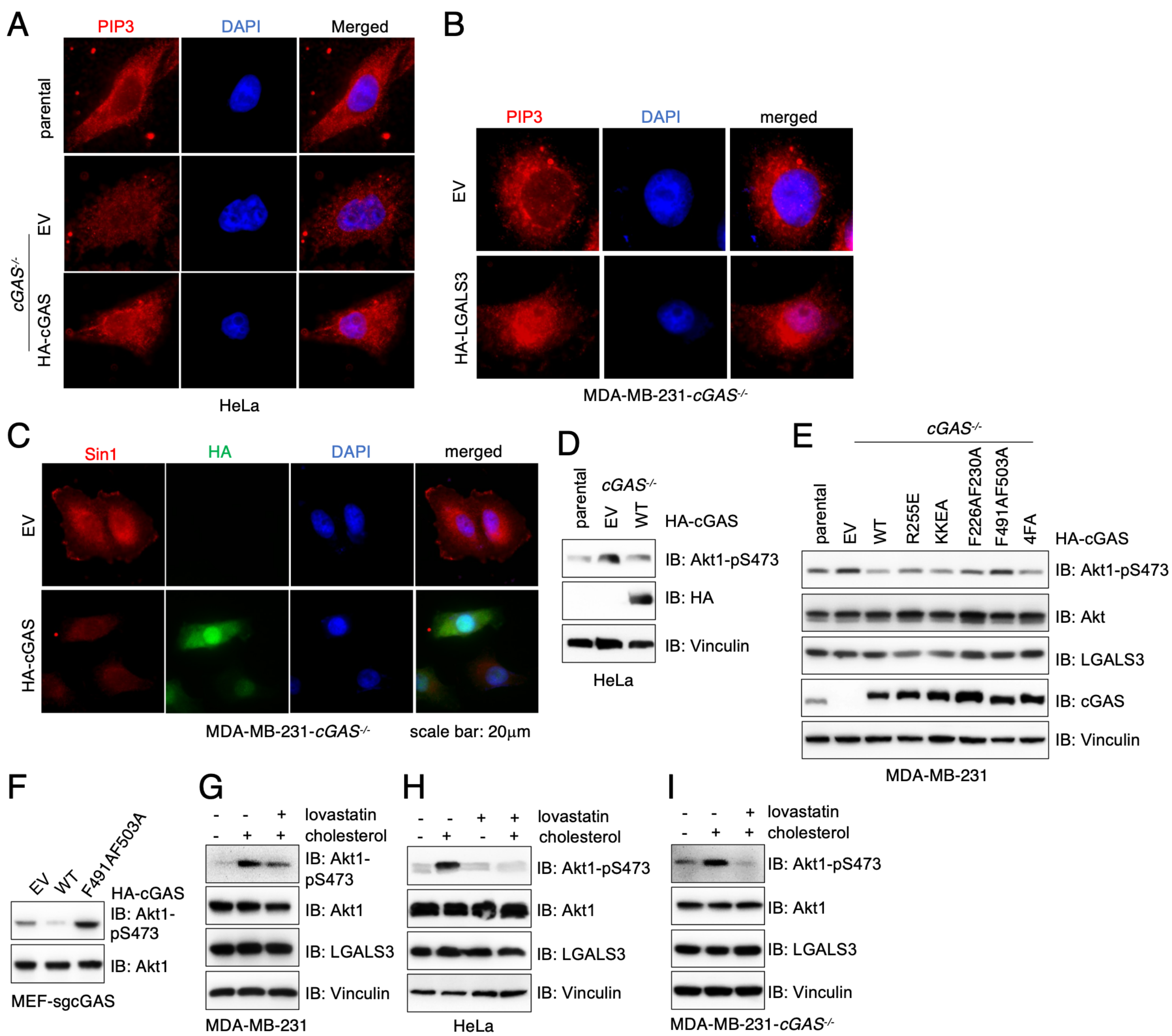

Figure S8. cGAS suppresses cellular cholesterol levels to reduce PI(3,4,5)P3 abundance and inhibit mTORC2/Akt signaling.

(A) Representative confocal images of PI(3,4,5)P3 staining of indicated HeLa cells.

(B) Representative confocal images of PI(3,4,5)P3 staining of indicated MDA-MB-231 cells.

(C) Representative confocal images of Sin1 staining of indicated MDA-MB-231 cells.

(D) IB analysis of WCL from indicated HeLa cells.

(E) IB analysis of WCL from indicated MDA-MB-231 cells.

(F) IB analysis of WCL from indicated MEFs.

(G) IB analysis of WCL from MDA-MB-231 cells pretreated with 5 μM lovastatin for 2 hrs followed by stimulation by 100 μM cholesterol for 1.5 hrs prior to cell collection.

(H) IB analysis of WCL from HeLa cells pretreated with 5 mM lovastatin for 2 hrs followed by stimulation by 100 mM cholesterol for 1.5 hrs prior to cell collection.

(I) IB analysis of WCL from MDA-MB-231-*cGAS*$^{-/-}$ cells pretreated with 5 mM lovastatin for 2 hrs followed by stimulation by 100 mM cholesterol for 1.5 hrs prior to cell collection.

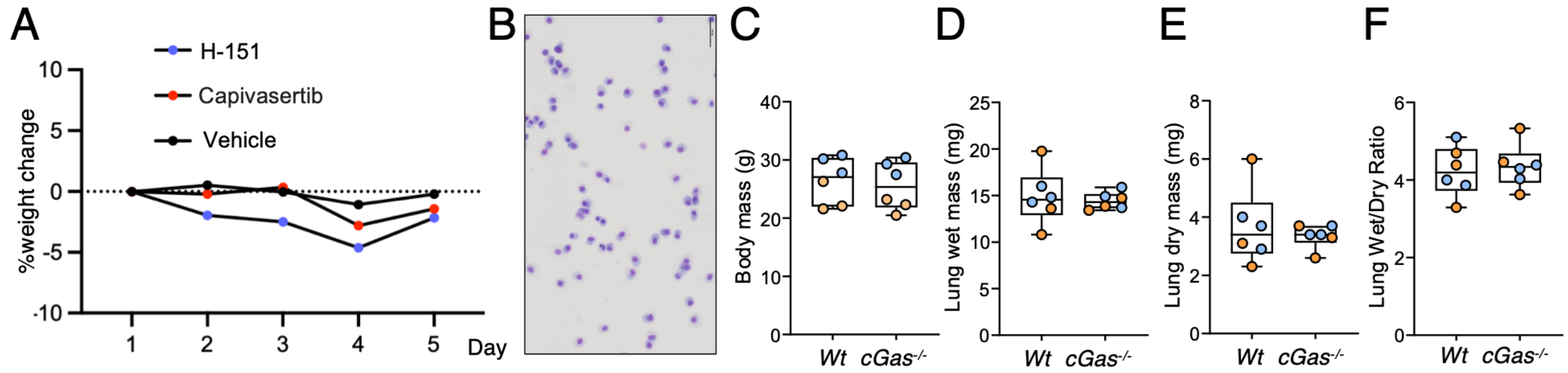

Figure S9. Characterization of lung phenotypes in adult *cGas$^{-/-}$* and *Wt* mice

(A) *cGas$^{-/-}$* C57BL/6 mouse body weights receiving indicated treatments.

(B) Representative cytospin image from a male *cGas*-KO mouse showing morphologically normal macrophages.

(C) Body weight measurements in *Wt* and *cGas*-KO mice.

(D-F) Wet/dry lung weight ratio analysis. (D) Wet weights measured after blotting. (E) Dry weights measured after 16 hours at 90°C. (F) Calculated wet/dry lung weight ratio. Only the accessory lobe was used. Blue dots = males; orange dots = females.

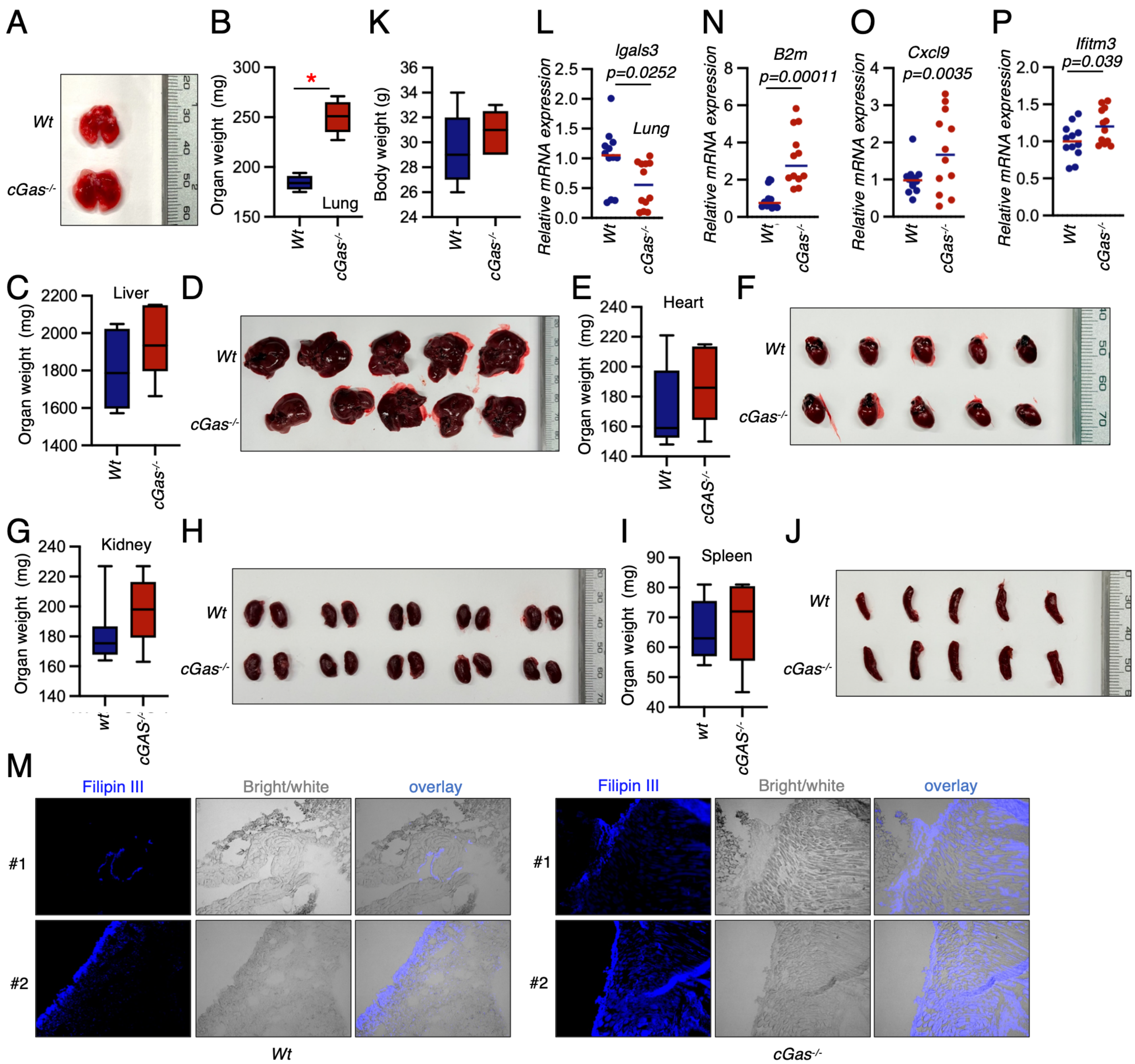

Figure S10. Characterization of lung phenotypes in adult *cGAS*$^{-/-}$ and *Wt* mice.
(A) Representative gross lung images from *Wt* and *cGd*-KO mice following CO_2 stress exposure.
(B) Wet lung weights from *Wt* and *cGas*-KO mice following CO_2 stress.
(C-J) Measurements of indicated organ weights (C, E, G, I) and representative images of collected organs (D, F, H, J).
(K) Body weights from the same cohort as in (A).
(L) RT-PCR analysis of *Lgals3* mRNA expression in lungs. Error bars represent mean ± SD; n = 3. $p < 0.05$ by one-way ANOVA.
(M) Representative Filipin III staining of snap-frozen lungs from *Wt* and *cGas*-KO mice indicating cholesterol accumulation.
(N-P) RT-PCR analysis of senescence and fibrosis-related gene expression (*B2m*, *Cxcl9*, *Ifitm3*) in lung tissue. Error bars represent mean ± SD; n = 3. $p < 0.05$ by one-way ANOVA.

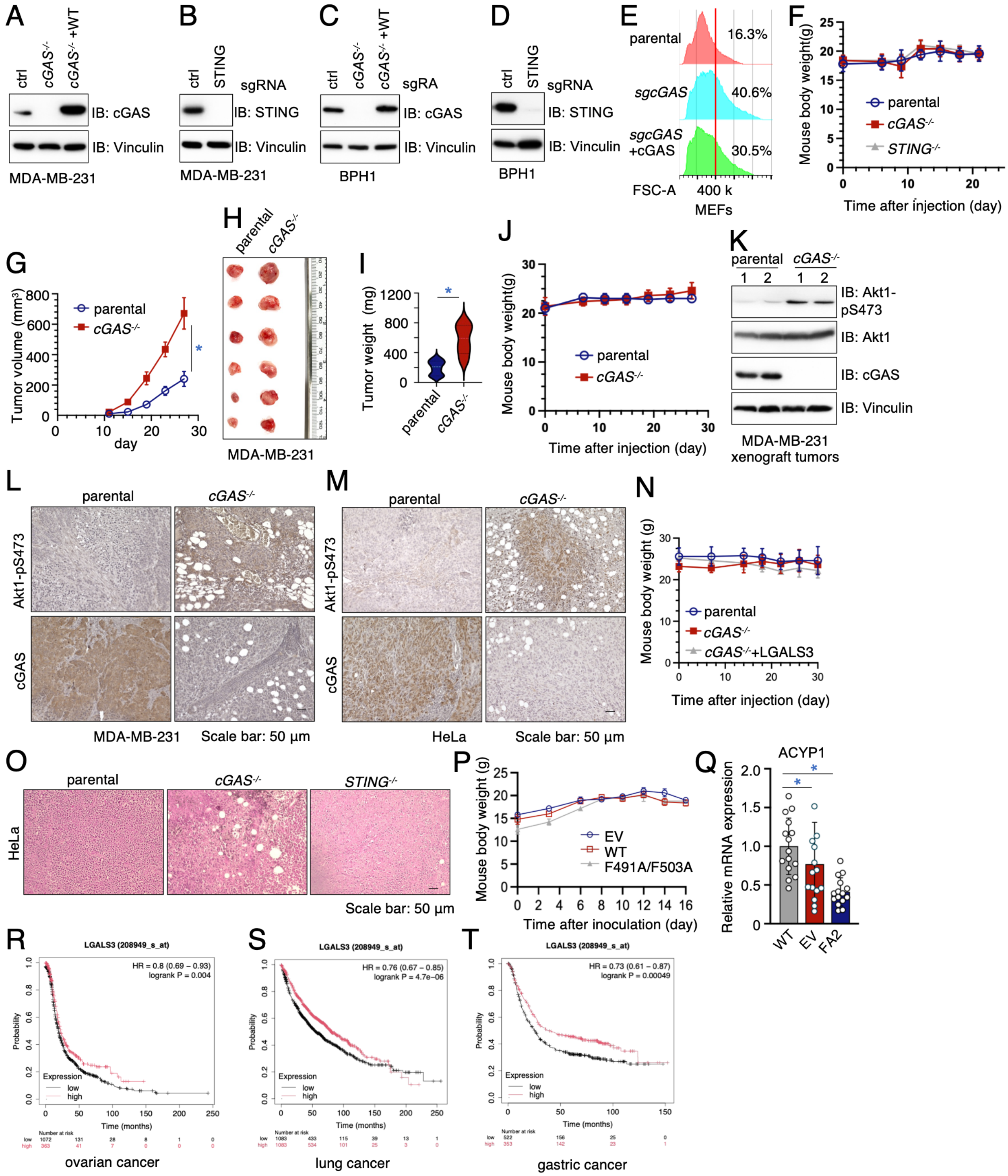

Figure S11. cGAS depletion facilitates xenografted tumor growth.
(A, B) IB analysis of WCL from indicated MDA-MB-231 cells.
(C, D) IB analysis of WCL from indicated BPH1 cells.
(E) Representative FACS analysis showing cell size distributions in the indicated THP1 cells.
(F, J, N, P) Measurements of mouse body weights at indicated time periods xenografted

with indicated tumor cells.
(G) Tumor volume measurements over time for xenografted cell lines. Error bars represent mean ± SD, n = 6. *p<0.05 (one-way ANOVA test).
(H) Images of isolated tumors from (G) with corresponding tumor weights shown in (I). Error bars represent mean ± SD, n = 6. *p<0.05 (one-way ANOVA test).
(K) IB analysis of WCL from indicated collected xenografted MDA-MB-231 tumors.
(L) Representative IHC analysis images using collected xenografted tumors from (H).
(M) Representative IHC analysis images using collected xenografted tumors from xenografted indicated HeLa tumors.
(O) Representative H&E staining images for collected xenografted indicated HeLa tumors.
(Q) RT-PCR analysis of ACYP1 mRNA expression in indicated tumors harvested in (P). Error bars were calculated as mean+/-SD, n=3. *p < 0.05 (one-way ANOVA test).
(R-T) Higher levels of LGALS3 expression correlates with improved patient outcome in indicated cancer types.

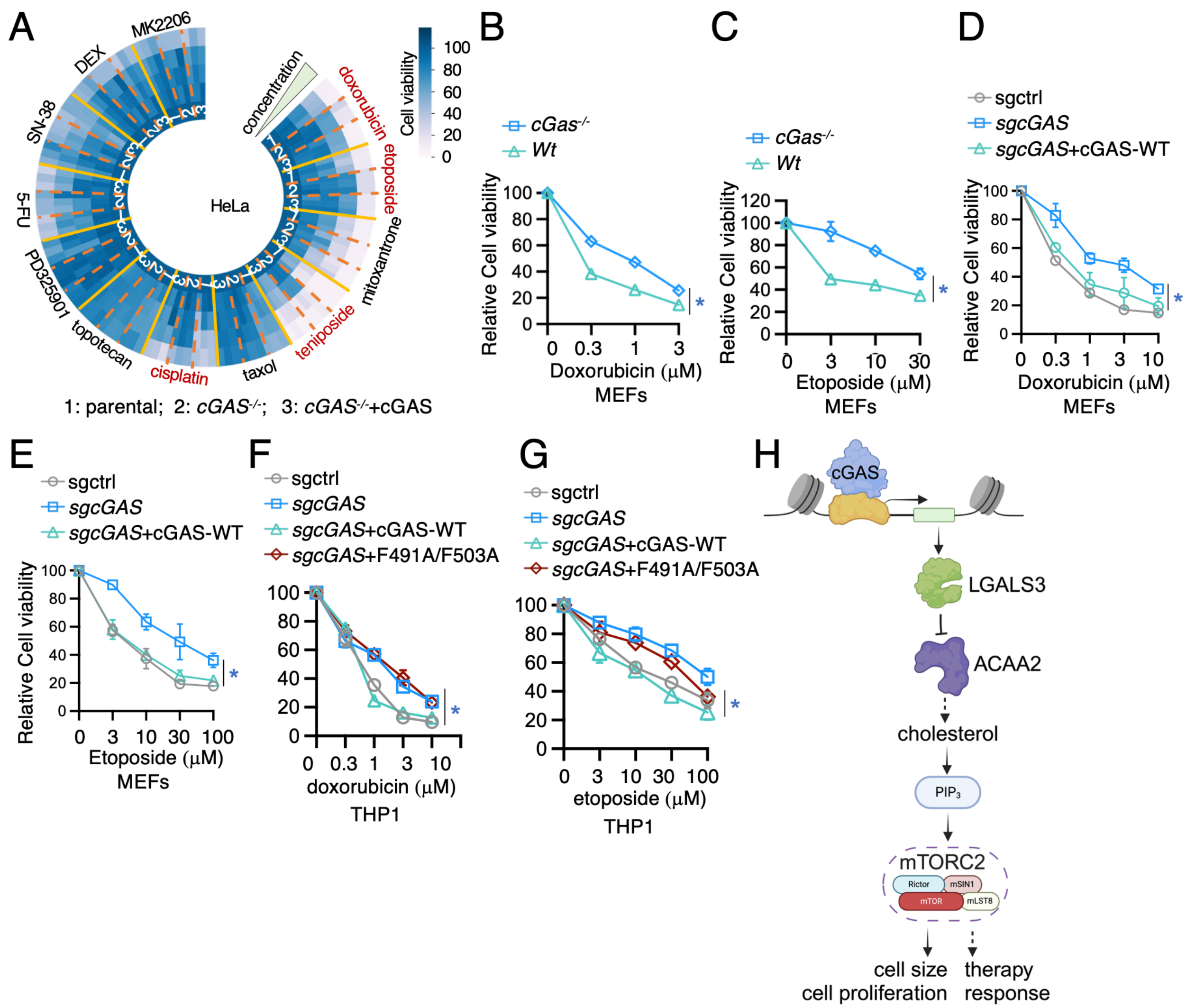

Figure S12. cGAS sensitizes cells to doxorubicin and etoposide treatments.

(A) Heatmap displaying cell responses to the indicated treatments.

(B-E) MTT assays using the indicated MEF cells treated for 3 days with indicated agents at the indicated doses. Error bars represent mean ± SD, n = 3. *p<0.05 (one-way ANOVA test).

(F-G) MTT assays using the indicated THP1 cells treated for 3 days with indicated agents at the indicated doses. Error bars represent mean ± SD, n = 3. *p<0.05 (one-way ANOVA test).

(H) A schematic illustration of our proposed model: cGAS associates with the AP-1 transcription factor on chromatin to transcribe LGALS3. LGALS3 then binds to and suppresses ACAA2 activity, leading to reduced cellular acetyl-CoA levels and subsequently decreased cholesterol levels, which suppresses mTORC2/Akt signaling. This novel cGAS/LGALS3/ACAA2/cholesterol/mTORC2/Akt signaling pathway regulates tumor growth and modulates responses to DNA-damaging agents such as doxorubicin and etoposide.

Table S1: KEY RESOURCES TABLE

REAGENT or RESOURCE	SOURCE	IDENTIFIER
Antibodies		
Anti-HA-Tag antibody	Cell Signaling Technology	cat#3724
Anti-phospho-TBK1/NAK (Ser172) antibody	Cell Signaling Technology	cat#5483
Anti-TBK1 antibody	Cell Signaling Technology	cat#51872
Anti-STING antibody (13647)	Cell Signaling Technology	cat#13647
Anti-phospho-STING (Ser366) antibody	Cell Signaling Technology	cat#50907
Anti-IRF3 antibody	Cell Signaling Technology	cat#4302
Anti-phospho-IRF-3 (Ser386) antibody	Cell Signaling Technology	cat#37829
Anti-phospho-IRF-3 (Ser396) antibody	Cell Signaling Technology	cat#29047
Anti-cGAS antibody	Cell Signaling Technology	cat#83623
Anti-Phospho-Histone H2A.X (Ser139)	Cell Signaling Technology	cat#9718
Anti-GST-tag antibody	Santa Cruz Biotechnology	cat# sc-459
Anti-c-Jun antibody	Santa Cruz Biotechnology	cat# sc-1694
Anti-rabbit IgG, HRP-linked antibody	Cell Signaling Technology	cat#7074
Anti-mouse IgG, HRP-linked antibody (7076)	Cell Signaling Technology	cat#7076
Polyclonal anti-Flag antibody	Sigma-Aldrich	cat#F7425
Monoclonal anti-Flag antibody	Sigma-Aldrich	cat#F-3165
Monoclonal anti-Tubulin antibody	Sigma-Aldrich	cat#T-5168
Anti-vinculin antibody	Santa Cruz Biotechnology	cat#sc-25336
Anti-GFP antibody	Abcam	cat# ab13970
Anti-JunB antibody	Cell Signaling Technology	cat#3753
Anti-FosL1 antibody	Abclonal	cat#A5372
Anti-Histone H3 antibody	Cell Signaling Technology	cat#4499
Anti-Histone-H4 antibody	Cell Signaling Technology	cat#13919
Anti-Galectin3 (LGALS3) antibody	Proteintech	cat#60207-1
Anti-phospho-Akt (Ser473) antibody	Cell Signaling Technology	cat#4060
Anti-Akt antibody	Cell Signaling Technology	cat#9272
Anti-ACAA2 antibody	Santa Cruz Biotechnology	cat#sc-100847
Anti-SMARCA5	Proteintech	cat#13066-1-AP
Anti-SMARCB1	Abcam	cat#EPR6966
Anti-SSRP1	Proteintech	cat#15696-1-AP
Anti-KMT2A	Proteintech	cat#29278-1-AP
Anti-HDAC1	Proteintech	cat#10197-1-AP
Nucleotide stimulants		
ISD90	Eurofins Genomics	5'-TACAGATCTACTAGTGATCTATGACTGATCTG

		TACATGATCTACATACA GATCTACTAGTGATCT ATGACTGATCTGTACA TGATCTACA-3'
Antibiotics		
Polyethylenimine (PEI)	Polysciences, Inc.	cat# 23866-1
Blasticidin	Sigma	cat# 15205
Puromycin	Fisher BioReagents	cat# 58-58-2
Hygromycin	Sigma	cat# H3274
Experimental models: Cell lines		
MDA-MB-231	ATCC	
A498	Dr. William Kim (UNC-Chapel Hill)	
HEK293T	ATCC	
BPH-1	Dr. Haojie Huang (Mayo Clinic)	
Software and algorithms		
Graphpad Prism 8	Prism	
Other		
Rneasy Mini Kit	QIAGEN	cat# 74106
iScript™ Reverse Transcription Supermix for RT-qPCR	Bio-Rad	cat# 170-8891
Taq universal SYBR green supermix	Bio-Rad	cat# 172-5124
Protease Inhibitor Cocktail	Bimake	cat# B14012
Phosphatase inhibitor cocktail	Bimake	cat# B15001
Lovastatin	TCI	cat# L0214
Atorvastatin	TCI	cat# A2476
Doxorubicin	Fisher Bioreagent	cat# BP2316-005
Etoposide	TCI	cat# E0675
Capivasertib	MedChemExpress	cat# HY-15431
H-151	MedChemExpress	cat# HY-112693
Trimetazidine hydrochloride	MedChemExpress	cat# HY-B0968
Neutravidin agarose beads	Thermo Scientific	cat# 29200
Glutathione agarose beads	GE Healthcare	cat# 17-0756-05
Anti-HA agarose beads	Sigma-Aldrich	cat# A-2095
Anti-Flag agarose beads	Sigma-Aldrich	cat# A2220
Alexa Fluor 594 phalloidin	Thermo Fisher Scientific	cat# A12381
Acetyl-Coenzyme A Assay Kit	Sigma	cat# MAK039

Table S2: DNA oligos/primer sequence

Cloning primers

Primer name	DNA sequence
cGAS-BglII-F	GCATAGATCTCAGCCTTGGCACGGAAAGGC
cGAS-XhoI-R	GCATCTCGAGTCAAAATTCATCAAAAACTGG
cGAS-XbaI-F	GCATTCTAGAATGAGCCTTGGCACGGAAAG
cGAS-AgeI-nostop-R	GCATACCGGTAAATTCATCAAAAACTGGAAACTC
miniTurbo-AgeI-linker-F	CATACCGGTGGAGGATCGGGAGGAACTACAAAGACCATGACGG
miniTurbo-SalI-R	GCATGTCGACTCACTTTTCGGCAGACCGCAG
FosL1-BamHI-F	GCATGGATCCTTCCGAGACTTCGGGG
FosL1-SalI-R	GCATGTCGACGGGAGGAGCGAAACAC
STING-BamHI-F	GACACCGACTCTAGAGGATCCATGCCCCACTCCAGCCTGCA
STING-SalI-Flag-R	ATCCAGAGGTTGATTGTCGACTCACTTGTCGTCATCGTCTTTGTAGTCAGAGAAATCCGTGCGGAGAG
LAGLS3-BamHI-F	GCATGGATCCGCAGACAATTTTTCGCTCCATG
LAGLS3-SalI-R	GCATGTCGACTTATATCATGGTATATGAAGCACTGGTG
ACAA2-BamHI-F	GCATGGATCCGCTCTGCTCCGAGG
ACAA2-SalI-R	GCAT GTCGACTCAGGCTGTGCTCTGAATG

Mutagenesis primers

Primer name	DNA sequence
cGAS-K407EK411A-F	GAGAAATGTTGCAGGGAGGATTGTTTAGCGCTAATGAAATACCTTTTAGAACAGC
cGAS-K407EK411A-R	GCTGTTCTAAAAGGTATTTCATTAGCGCTAAACAATCCTCCCTGCAACATTTCTC
cGAS-R255E-F	GCATATTACTTTGTGAAATTTAAAGAAAATCCGAAAGAAAATCCTC
cGAS-R255E-R	GAGGATTTTCTTTCGGATTTTCTTTAAATTTCACAAAGTAATATGC
cGAS-F226AF230A-F	CACCTAATGAAGCTGATGTCATGGCTAAACTGGAAGTCCC
cGAS-F226AF230A-R	GGGACTTCCAGTTTAGCCATGACATCAGCTTCATTAGGTG
cGAS-F491AF503A-F	CAATCTAGCCTCTAGCAACTTAATTGACAAAAGAAGTAAGGAAGCTCTGAC
cGAS-F491AF503A-R	GTCAGAGCTTCCTTACTTCTTTTGTCAATTAAGTTGCTAGAGGCTAGATTG
cGAS-ΔTAD1-F	GCAGGATCCGGGGTTGTGGACCACCTGCTGCTCAGA
cGAS-ΔTAD1-R	GCAGTCGACTCTGAGCAGCAGGTGGTCCACAACCCC
cGAS-ΔTAD2-F	GCAGGATCCAATGAATTTGATGTCATGTTTAAACTG
cGAS-ΔTAD2-R	GCAGTCGACCAGTTTAAACATGACATCAAATTCATT
cGAS-ΔTAD4-F	GCAGGATCCAATCTATTCTCTAGCAACTTAATTGAC
cGAS-ΔTAD4-R	GCAGTCGACGTCAATTAAGTTGCTAGAGAATAGATT
cGAS-ΔTAD5-F	GCAGGATCCGAATTTCTGACAAAGCAAATTGAATAT
cGAS-ΔTAD5-R	GCAGTCGACATATTCAATTTGCTTTGTCAGAAATTC

sgRNA sequence

sgRNA plasmids were constructed by inserting synthesized sgRNAs into lentiCRISPRv2-puro vector or lentiCRISPRv2-blast vector. The target sequence is listed below:

Primer name	DNA sequence
sgSTING-1	CTGGGACTGCTGTTAAACG
sgLGALS3-1	CTGTTGTTCTCATTGAAGCG
sgLGALS3-4	CAGACCCAGATAACGCATCA
sgcGAS	GCACGTGCTCATAGTAGCTCC
sgJunB-1	GGGTAAAAGTACTGTCCCGG
sgJunB-2	GGACAGTACTTTTACCCCCG
sgSMARCB1-1	TGAGAACGCATCTCAGCCCG
sgSMARCB1-2	AGCCCCGACACGCCCAACAT
sgSMARCA5-1	ACATCTTTGTCACACACCGG
sgSMARCA5-2	ACTGATTTCCTTAAGACCGG

RT-PCR primers

Primer name	DNA sequence
hCEP97-F	GCACCGGAGAAACTCCAGCAATC
hCEP97-R	CACCATTGTGAGGCAGTTGAC
hKDM2A-F	CAAGGAGAGTGTGGTGTTTGCC
hKDM2A-R	ACCTCTCCACAGAGGGAACATG
hACYP1-F	GTGTTTTTCCGTAAGCATACTCAG
hACYP1-R	CCATTCCTGCATATGACGCACC
hCHMP4C-F	AGACTGAGGAGATGCTGGGCAA
hCHMP4C-R	TAGTGCCTGTAATGCAGCTCGC
hCFB-RT-F	TGAGGCTTCCTCCAACTACCAC
hCFB-RT-R	TGTAGACCTCCTTCCGAGTCAG
hCCDC82-F	CCGCTTTGTTCAGCCTCGTCTA
hCCDC82-R	AGTAGCGATGCAGTCCACAAGC
hAIM2-F	GCTGCACCAAAAGTCTCTCCTC
hAIM2-R	CTGCTTGCCTTCTTGGGTCTCA
hF11R-F	GTGAAGTTGTCCTGTGCCTACTC
hF11R-R	ACCAGTTGGCAAGAAGGTCACC
hBIRC2-F	CAGACACATGCAGCTCGAATGAG
hBIRC2-R	CACCTCAAGCCACCATCACAAC
hEXPH5-F	TGTCCCAAAGCCAGCAGTCATG
hEXPH5-R	CTGAGCTTGCAGACTGTTCCTG
hLGALS3-F	CCATCTTCTGGACAGCCAAGTG
hLGALS3-R	TATCAGCATGCGAGGCACCACT
hFADS1-F	CTGTCGGTCTTCAGCACCTCAA
hFADS1-R	CTGGGTCTTTGCGGAAGCAGTT
hFADS2-F	TGCAACGTGGAGCAGTCCTTCT
hFADS2-R	GGCACATAGAGACTTCACCAGC
hPPARG-F	AGCCTGCGAAAGCCTTTTGGTG
hPPARG-R	GGCTTCACATTCAGCAAACCTGG
mcGAS-F	TTCCACGAGGAAATCCGCTGAG
mcGAS-R	CAGCAGGGCTTCCTGGTTTTTC
mLGALS3-F	AACACGAAGCAGGACAATAACTGG
mLGALS3-R	GCAGTAGGTGAGCATCGTTGAC
mB2m-F	CATGGCTCGCTCGGTGAC
mB2m-R	CAGTTCAGTATGTTCGGCTTCC

mCxcl9-F	CGAGGCCACGATCCACTACAA
mCxcl9-R	AGGCAGGTTTGATCTCCGTT
mIfitm3-F	TATGAGGTGGCTGAGATGGG
mIfitm3-R	TCACCCACCATCTTCCGATC
mGapdh-F	GTCATCCCAGAGCTGAACG
mGapdh-R	TCATACTTGGCAGGTTTCTCC

Supplemental methods

Micro-C

EV and WT-cGAS expressing MDA-MB-231 cells were subjected to micro-C analysis. The Dovetail Micro-C kit was used for sample preparation following the manufacturer's instructions (https://dovetailgenomics.com/wp-content/uploads/2021/09/Dovetail%E2%84%A2-Micro-C-Kit-User-Guide-Version-1.0.pdf). DNA sequencing was performed by UNC HTSF facility and fastq format data were obtained.

Conversion of fastq data to valid pairs bam file was performed following instructions provided by Dovetail (https://micro-c.readthedocs.io/en/latest/fastq_to_bam.html). Briefly, GRCh38 primary genome assembly was downloaded from GENCODE (https://www.gencodegenes.org/, release_32 at the time analysis was done). Various software program versions used were as follows: 1) gatk/4.1.9.0 2) bedtools/2.29 3) deeptools/3.2.0 4) bwa/0.7.17 5) python/3.6.6 6) pairtools/0.3.0 7) samtools/1.13 8) pairix/0.3.7 9) cooler/0.8.6 10) juicer_tools/1.22.01. Data was aligned and processed using the command described in: https://micro-c.readthedocs.io/en/latest/fastq_to_bam.html All operations are carried out in a single step line by using unix pipes. Briefly, paired-end fastq data was aligned using bwa mem using the parameters noted on the page above. bwa mem outpus a SAM file that is directly piped into paitrools parse program to find ligations junctions in libraries. Parsed read pairs are the sorted using paitrools sort program. pairtools dedupe program then detects molecules that could be formed by PCR duplication. pairtools split program is then used to split the aligned files into a pair of .sam and a .pairs file. The .sam file is then sorted using samtools and an index is generated for the sorted BAM file using samtools index. Contact maps in ".hic" format were then generated using Juicer tools program. Cooler tools cooler cload was used to load the sorted and indexed .pair files to generate the high resolution .cool contact matrices. Finally multi-resolution .mcool files were produced using cooler zoomify.

Micro-C loop detection and differential analysis

Loops were identified using SIP (2) (version 1.6.1) with the following settings "-g 2.0 -min 2.0 -max 2.0 -mat 2000 -d 6 -res 5000 -sat 0.01 -t 2000 -nbZero 6 -factor 1 -fdr 0.05 -del true -cpu 1 -isDroso false". To remove redundancy, loops from all conditions were merged in R with mariner (3) using the mergePairs function, with merging based on "APScoreAvg", creating a list of 28,780 loops. A count matrix was prepared using mariner, where unnormalized counts at each loop pixel from each biological replicate were extracted. DESeq2 was used to identify differential loops using the prepared count matrix. Counts from each biological replicate were used with the design "~replicate + condition". Apeglm was used to calculate $\log_2$ fold changes for each loop, comparing WT to EV. Differential loops were defined as those with an adjusted p-value < 0.1 and an absolute log2(fold-change) > 1.5.
CUT&RUN was performed with modifications to the manufacturer's protocol using the CUT&RUN Assay Kit (Cell Signaling Technology, #86652). Briefly, 5 × 10^5 cells were used per sample, including input controls. Concanavalin A (ConA) beads were activated according to the manufacturer's instructions and incubated with cells for 10 min at room temperature. Bead-bound cells were incubated with the indicated antibodies (1:100) overnight at 4°C with nutation. After washing with ice-cold digitonin buffer, pAG-MNase was added and incubated for 10 min at room temperature. Following two additional washes, MNase was activated with 100 mM $CaCl_2$ and incubated for 2 h at 4°C. DNA release was initiated with stop buffer and incubation at 37°C for 10 min. The supernatant was collected after magnetic separation, and DNA was purified using a PCR purification kit and eluted in 10 µL of 0.1× TE buffer.

CUT&RUN

CUT&RUN analysis was performed with modifications to the manufacturer's instructions using

the CUT&RUN Assay Kit (Cell Signaling Technology #86652). Briefly, 5 × 10^5 indicated cells were used per assay, including input samples.

Preparation of Concanavalin A (ConA) Beads: ConA beads (11 µL per sample) were transferred to a 1.5 mL tube on a magnetic separation rack until the slurry became clear. The supernatant was removed, and 100 µL of bead activation buffer per sample was added. The beads were gently pipetted to mix, and the slurry was cleared on the magnetic rack. This activation step was repeated twice. The beads were resuspended in 11 µL of bead activation buffer per sample, and 10 µL of the suspension was aliquoted into 8-strip PCR tubes on ice.

Cell Preparation: Cells were trypsinized and collected by centrifugation at 600 × g for 3 minutes. The pellet was resuspended in 100 µL of wash buffer (10 µL 10× wash buffer, 10 µL 100× spermidine, 5 µL 200× protease inhibitor cocktail, 75 µL water) and centrifuged again at 600 × g for 3 minutes. The cells were washed three times and resuspended in 100 µL of wash buffer. Each 100 µL aliquot of cells was transferred to the PCR tubes containing activated beads and mixed gently by pipetting. The cells and beads were incubated at room temperature for 10 minutes to allow binding.

Antibody Binding: The beads-bound cells were collected on a magnet, the supernatant was removed, and 50 µL of cold antibody binding buffer (0.5 µL 100× spermidine, 0.25 µL 200× protease inhibitor cocktail, 1.25 µL digitonin solution, and 48 µL antibody binding buffer) was added to each sample. After gentle mixing, 0.5 µL of antibody (1:100 dilution) was added to each tube, and the tubes were incubated on a nutator at 4°C overnight.

Washing and Enzyme Binding: The beads-bound cells were collected on a magnet, and the supernatant was removed. Ice-cold digitonin buffer (25 µL 10× wash buffer, 2.5 µL 100× spermidine, 1.25 µL 200× protease inhibitor cocktail, 6.25 µL digitonin, 215 µL Milli-Q water) was added (250 µL per sample) and gently mixed with the beads by pipetting. This wash step was repeated twice. Next, 2.5 µL of pAG-MNase enzyme was added to each tube, mixed gently, and incubated at room temperature for 10 minutes. The beads were washed twice with 250 µL of ice-cold digitonin buffer and resuspended in 50 µL of fresh ice-cold digitonin buffer.

MNase Activation: To activate MNase, 1 µL of 100 mM $CaCl_2$ was added to each sample and mixed thoroughly. The tubes were incubated on a nutator at 4°C for 2 hours.

DNA Release and Purification: Stop buffer (33 µL; 8.25 µL 4× stop buffer, 0.825 µL digitonin solution, 0.165 µL RNase A, 23.76 µL Milli-Q water) was added to each sample and mixed well by pipetting. The tubes were incubated in a thermocycler at 37°C for 10 minutes. The slurry was cleared on a magnet, and the supernatant was transferred to 1.5 mL tubes. DNA was purified using PCR product purification kits and eluted in 10 µL of elution buffer (0.1x TE buffer).

CUT&RUN DNA library construction

CUT&RUN libraries were prepared using the NEBNext Ultra II DNA Library Prep Kit for Illumina (E7645) according to the manufacturer's instructions. Briefly, fragmented DNA was diluted in 0.1× TE to 50 µL and subjected to end repair/dA-tailing (20°C for 30 min, 50°C for 30 min). Adapters were ligated at 20°C for 15 min, followed by USER enzyme treatment at 37°C for 15 min. Adaptor-ligated DNA was purified using SPRIselect beads without size selection and eluted in 15 µL of 0.1× TE. Libraries were amplified with NEBNext Ultra II Q5 Master Mix and indexed primers for 15 cycles (98°C for 30 s; 98°C for 10 s, 65°C for 75 s; final extension at 65°C for 5 min). PCR products were purified using SPRIselect beads, eluted in 0.1× TE, and subjected to Illumina sequencing.

CUT&RUN DNA-sequencing

Sequencing of constructed *CUT&RUN* DNA library was performed by NOVOGEN (NovaSeq X Plus PE150).

CUT&RUN data analysis

Sequencing adapters on reads were trimmed using cutadapt (4) (v2.9) using options -a GATCGGAAGAGC -A GATCGGAAGAGC and --minimum-length 36 in paired mode. After trimming, reads were filtered for quality using the fastq_quality_filter in FASTX-Toolkit (v0.0.14) (http://hannonlab.cshl.edu/fastx_toolkit/), with options -Q 33, -p 90, and -q 20. In-house scripts were used to limit potential PCR duplicates by limiting reads with the same sequence to a maximum of five copies and discarding the copies beyond that limit. Because the previous filtering steps may remove one end of a read pair but not the other, both read pair FASTQ files were synced using in-house scripts to ensure proper order for alignment. Read alignment was performed against the hg19 genome using STAR (5) (v2.5.2b) and options --outFilterMismatchNmax 2, --chimSegmentMin 15, --chimJunctionOverhangMin 15, --outSAMtype BAM Unsorted, --outFilterType BySJout, --outFilterScoreMin 1, and --outFilterMultimapNmax 1. Bam files were filtered for primary alignments with SAMtools (6) view and option -F 256 (v1.31). Peaks were called on bam files using Macs (7) (v2.2.9.1) with option —nomodel and two replicates of Empty Vector as a merged negative control. Peak narrowPeak files were subsequently loaded into R (8) (v4.4.2) and subset to mappable chromosomes (chr1:22,X,Y) with GenomicRanges (9) (v1.58.0). Bigwig files were generated from quality filtered bam files with deepTools (10) bamCoverage (v3.5.4) and RPKM normalization. RPKM normalized BigWigs were then z-normalized per chromosome with a custom R script.

cGAS binding peaks were filtered to exclude broad peaks (>10 kb) and peaks with q-values below the 10th percentile for each sample. Reproducible peaks were defined as those present in at least two of three replicates. For each condition, peaks were merged into a union set, retaining regions overlapping in the majority of replicates. Intersections of high-confidence peak sets were used to define three cGAS-binding categories. Genomic annotations were assigned using ChIPseeker (11) package (v1.43.0) using TxDb.Hsapiens.UCSC.hg19.knownGene (v3.2.2).

Differential peak analysis was performed using DESeq2 (12) (v1.46.0). A full union set of cGAS peaks across all conditions was generated using mappable chromosomes without additional filtering. Read counts were quantified from BAM files using Rsubread (v2.20.0), followed by differential analysis of two contrasts: WT rescue versus cGAS-R255E rescue and WT rescue versus endogenous cGAS (shScr).

Motif analysis was performed in R with the memes package (v1.14.0) and the Meme Suite (13) (v5.5.7). Analysis of Motif Enrichment (AME) (14) was run on 200bp windows around peak centers for each cGAS binding category. To optimize computation, peaks in each cGAS binding category were further refined by results from differential analysis. True-cGAS peaks were refined to peaks with the least difference between WT rescue and $cGAS^{R255E}$ rescue ($-0.5 < log_2FoldChange < 0.5$, padj > 0.1, n=9,002). Nucleosomal cGAS peaks were refined to those peaks with significantly lower counts in WT cGAS rescue versus $cGAS^{R255E}$ rescue ($log_2FoldChange <= -1$ and padj <= 0.1, n=2,849). Neo-gained cGAS peaks were refined to those peaks with significantly higher counts in $cGAS^{R255E}$ rescue versus WT cGAS rescue ($log_2FoldChange >= 1$ and padj <=0.1, n=48,980). Because of the very large number of Neo-gained cGASE peaks it was computationally prohibitive to run AME on all regions, so these were randomly subsampled to 10,000 regions. Denovo motif enrichment of True-cGAS regions was rung with STREME and motifs were matched to top hits with Tomtom (15) and the HOCOMOCO (16) transcription factor motif database (v12).

Differential volcano plots and average signal plots were generated with ggplot2 (17) (v3.5.1). Heatmaps of cGAS peak categories were generated with deepTools (v3.5.4). Browser shots were generated in R with the Gviz (18) packaged (v1.50.0). Analysis in R utilized Bioconductor (19) (v3.20).

References:

1. Liu J, Chen H, Kaniskan HU, Xie L, Chen X, Jin J, et al. TF-PROTACs Enable Targeted Degradation of Transcription Factors. *J Am Chem Soc.* 2021;143(23):8902-10.
2. Rowley MJ, Poulet A, Nichols MH, Bixler BJ, Sanborn AL, Brouhard EA, et al. Analysis of Hi-C data using SIP effectively identifies loops in organisms from C. elegans to mammals. *Genome Res.* 2020;30(3):447-58.
3. Davis ES, Parker SM, Kramer NE, Flores JP, Kiran M, and Phanstiel DH. Mariner: explore the Hi-Cs. *Bioinformatics.* 2024;40(6).
4. Kechin A, Boyarskikh U, Kel A, and Filipenko M. cutPrimers: A New Tool for Accurate Cutting of Primers from Reads of Targeted Next Generation Sequencing. *J Comput Biol.* 2017;24(11):1138-43.
5. Dobin A, Davis CA, Schlesinger F, Drenkow J, Zaleski C, Jha S, et al. STAR: ultrafast universal RNA-seq aligner. *Bioinformatics.* 2013;29(1):15-21.
6. Li H, Handsaker B, Wysoker A, Fennell T, Ruan J, Homer N, et al. The Sequence Alignment/Map format and SAMtools. *Bioinformatics.* 2009;25(16):2078-9.
7. Zhang Y, Liu T, Meyer CA, Eeckhoute J, Johnson DS, Bernstein BE, et al. Model-based analysis of ChIP-Seq (MACS). *Genome Biol.* 2008;9(9):R137.
8. Liao Y, Smyth GK, and Shi W. The R package Rsubread is easier, faster, cheaper and better for alignment and quantification of RNA sequencing reads. *Nucleic Acids Res.* 2019;47(8):e47.
9. Lawrence M, Huber W, Pages H, Aboyoun P, Carlson M, Gentleman R, et al. Software for computing and annotating genomic ranges. *PLoS Comput Biol.* 2013;9(8):e1003118.
10. Ramirez F, Ryan DP, Gruning B, Bhardwaj V, Kilpert F, Richter AS, et al. deepTools2: a next generation web server for deep-sequencing data analysis. *Nucleic Acids Res.* 2016;44(W1):W160-5.
11. Yu G, Wang LG, and He QY. ChIPseeker: an R/Bioconductor package for ChIP peak annotation, comparison and visualization. *Bioinformatics.* 2015;31(14):2382-3.
12. Love MI, Huber W, and Anders S. Moderated estimation of fold change and dispersion for RNA-seq data with DESeq2. *Genome Biol.* 2014;15(12):550.
13. Nystrom SL, and McKay DJ. Memes: A motif analysis environment in R using tools from the MEME Suite. *PLoS Comput Biol.* 2021;17(9):e1008991.
14. McLeay RC, and Bailey TL. Motif Enrichment Analysis: a unified framework and an evaluation on ChIP data. *BMC Bioinformatics.* 2010;11:165.
15. Gupta S, Stamatoyannopoulos JA, Bailey TL, and Noble WS. Quantifying similarity between motifs. *Genome Biol.* 2007;8(2):R24.
16. Vorontsov IE, Eliseeva IA, Zinkevich A, Nikonov M, Abramov S, Boytsov A, et al. HOCOMOCO in 2024: a rebuild of the curated collection of binding models for human and mouse transcription factors. *Nucleic Acids Res.* 2024;52(D1):D154-D63.
17. Wickham H. *ggplot2: Elegant Graphics for Data Analysis.* Springer; 2016.
18. Florian Hahne RI. *Statistical Genomics: Methods and Protocols.* Springer New York; 2016.
19. Gentleman RC, Carey VJ, Bates DM, Bolstad B, Dettling M, Dudoit S, et al. Bioconductor: open software development for computational biology and bioinformatics. *Genome Biol.* 2004;5(10):R80.
