# Supplementary material for: Nuclear cGAS governs transcription to control lipid metabolism, tumor growth, and therapy response
Source: J Clin Invest. 2026 Nov 2;136(21):e205579. doi: 10.1172/JCI205579 (PMC13722394; doi:10.1172/JCI205579)

Full unedited blot for Figure 1

A

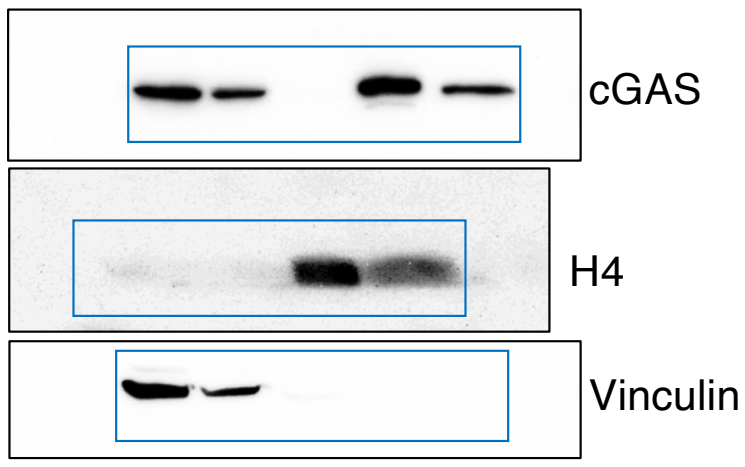

Full unedited blot for Figure 4

C

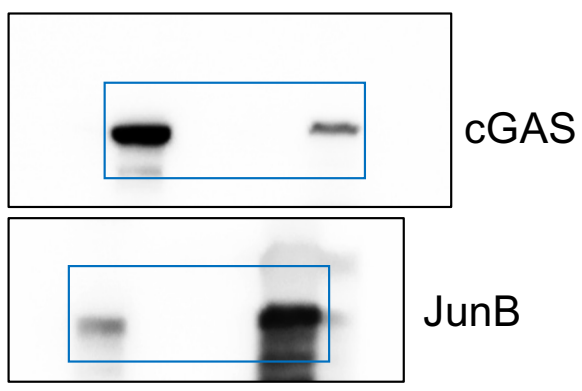

D

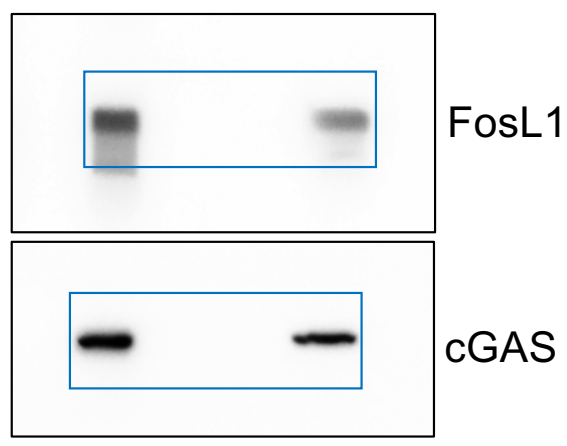

E

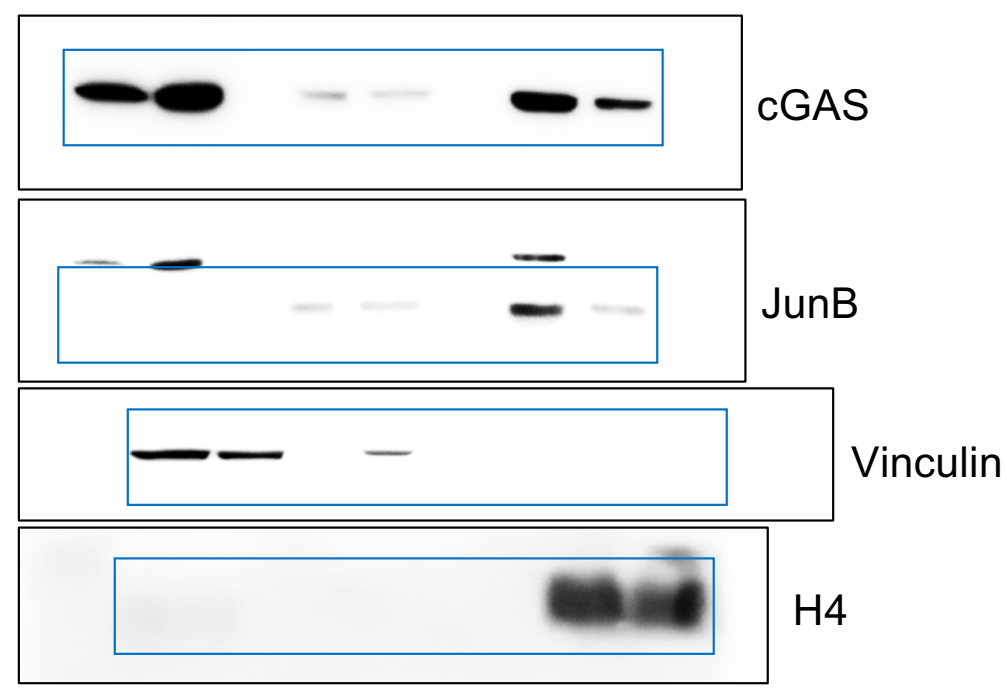

F

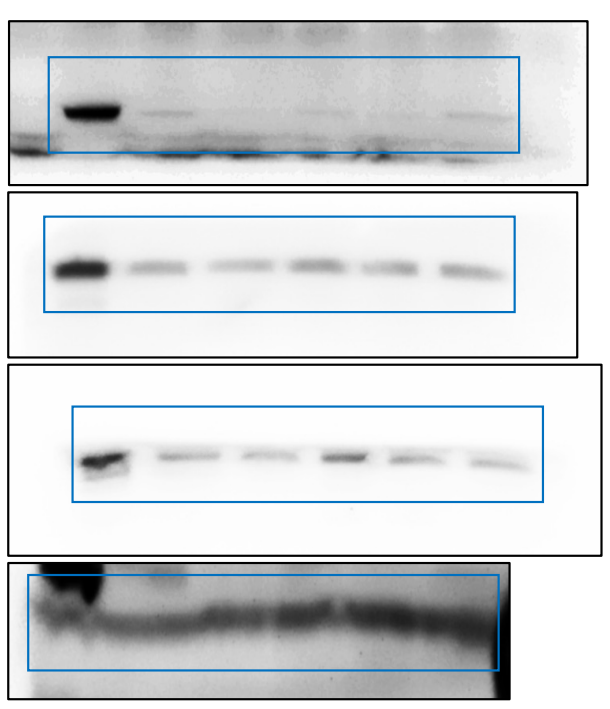

Full unedited blot for Figure 5

C

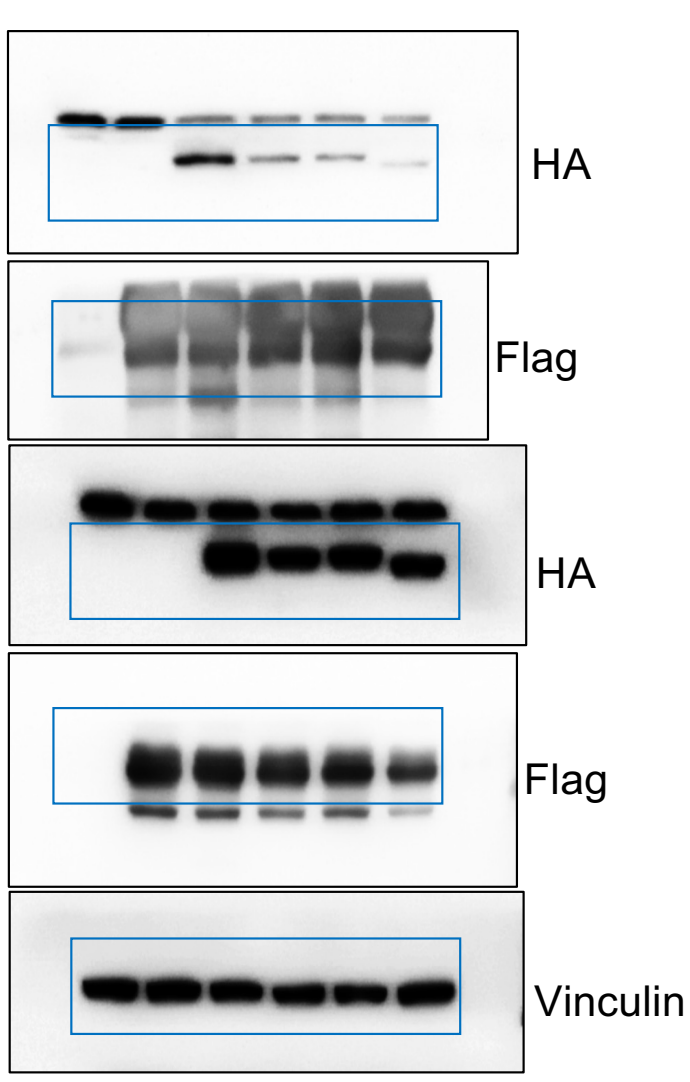

D

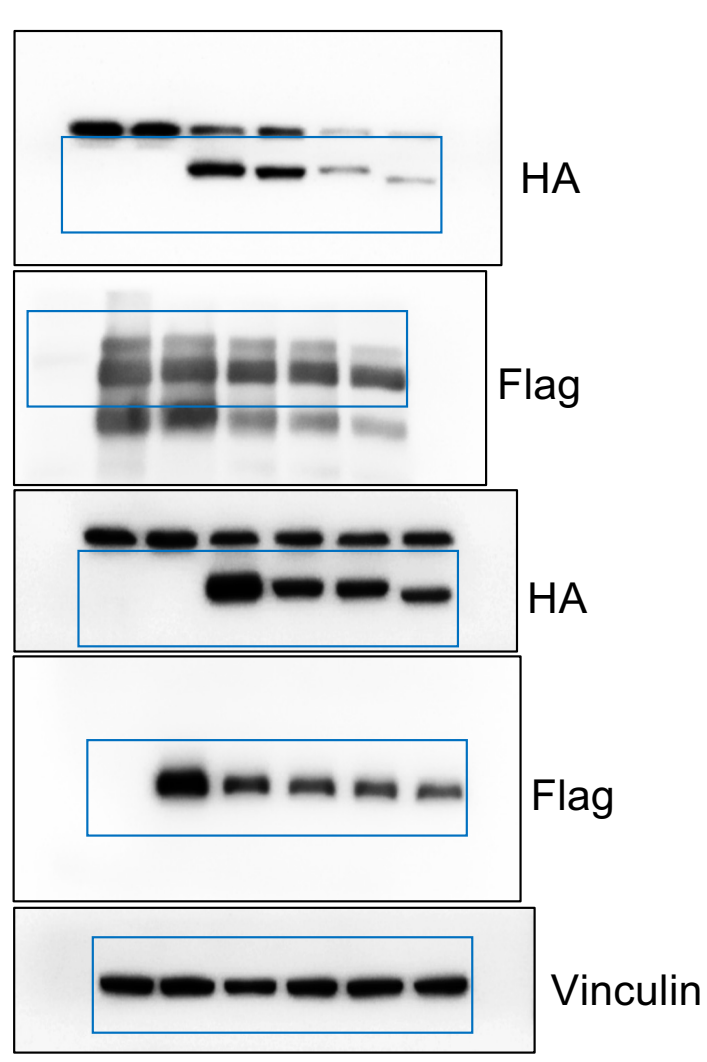

E

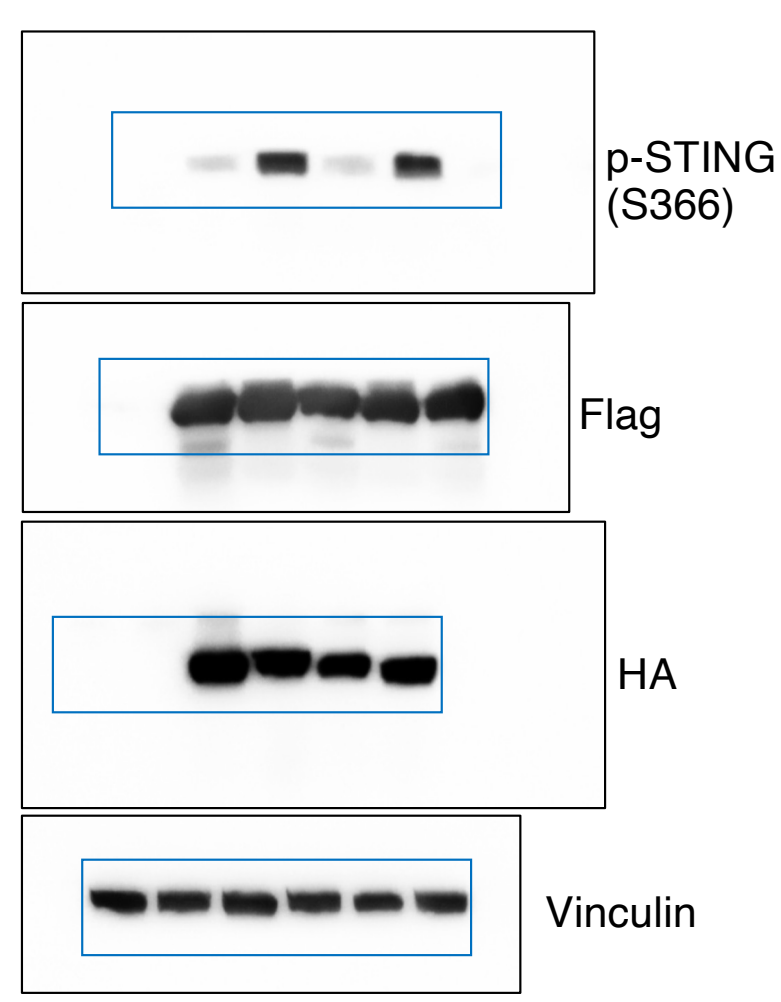

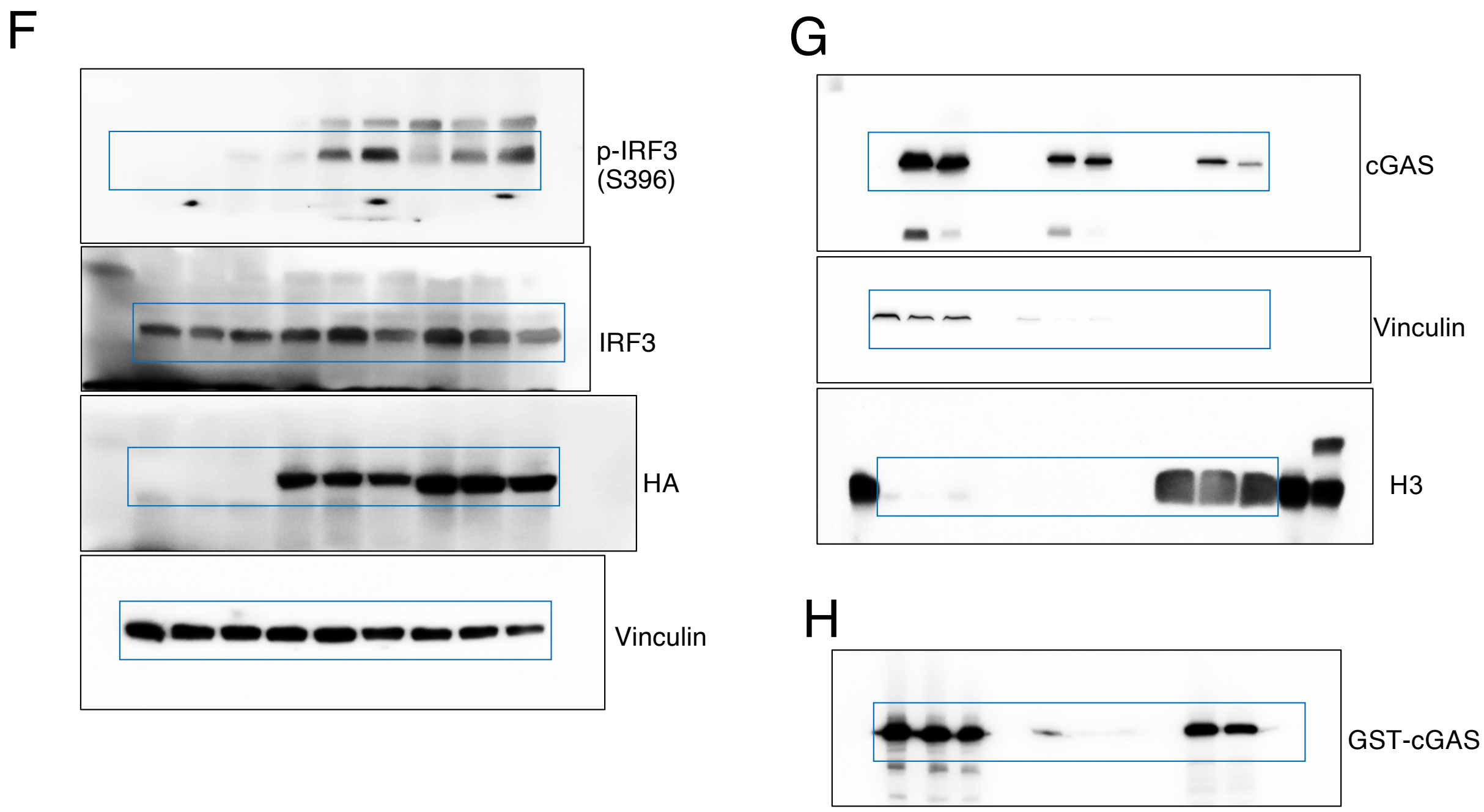

Full unedited blot for Figure 7

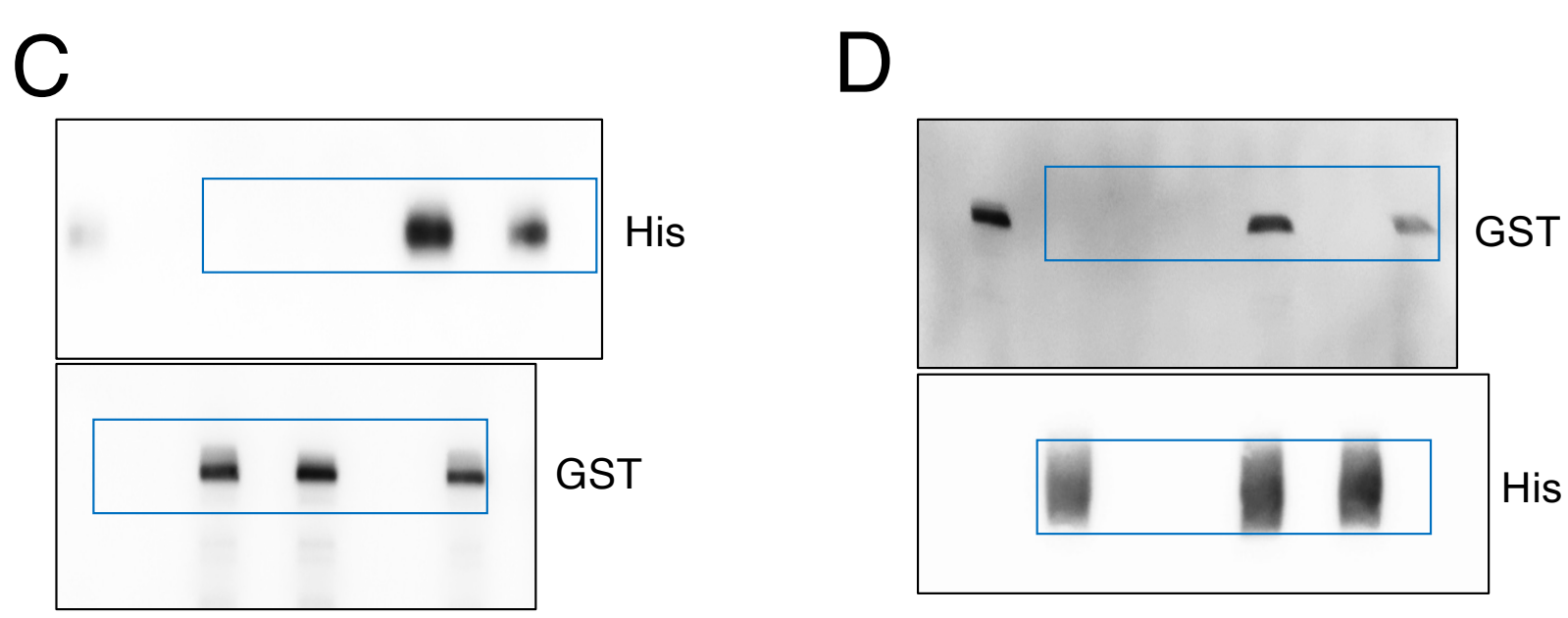

Full unedited blot for Figure 8

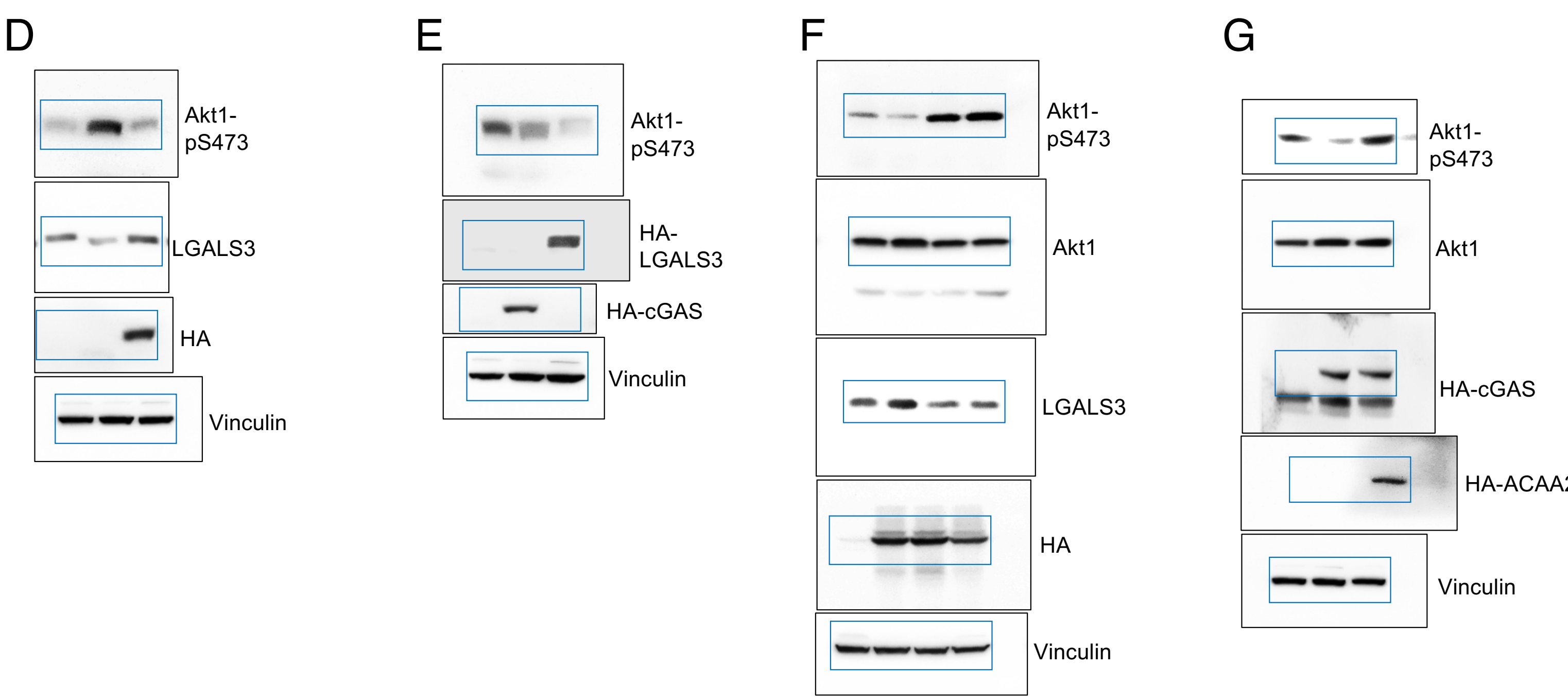

H

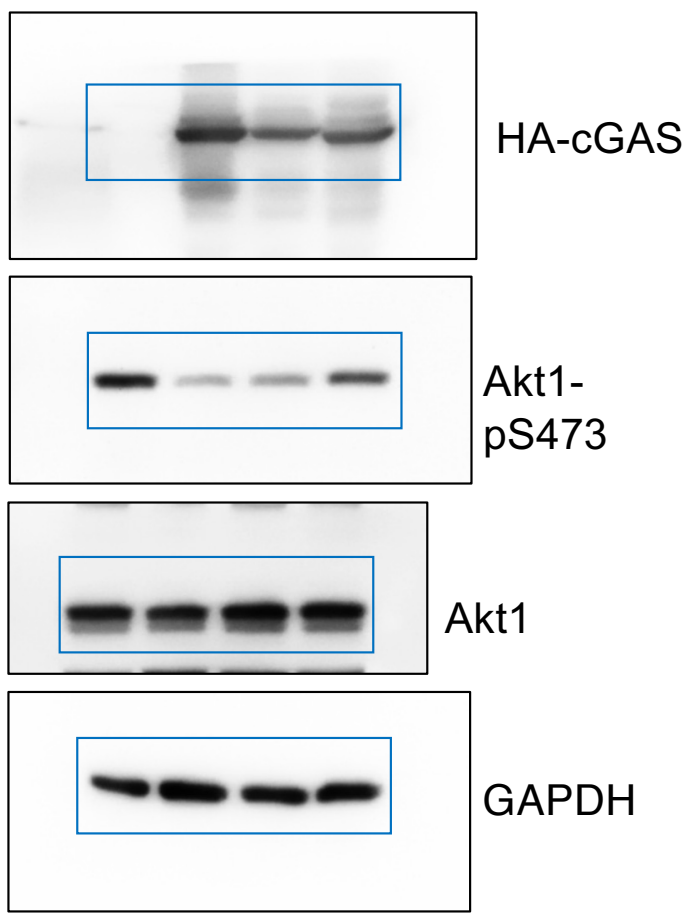

I

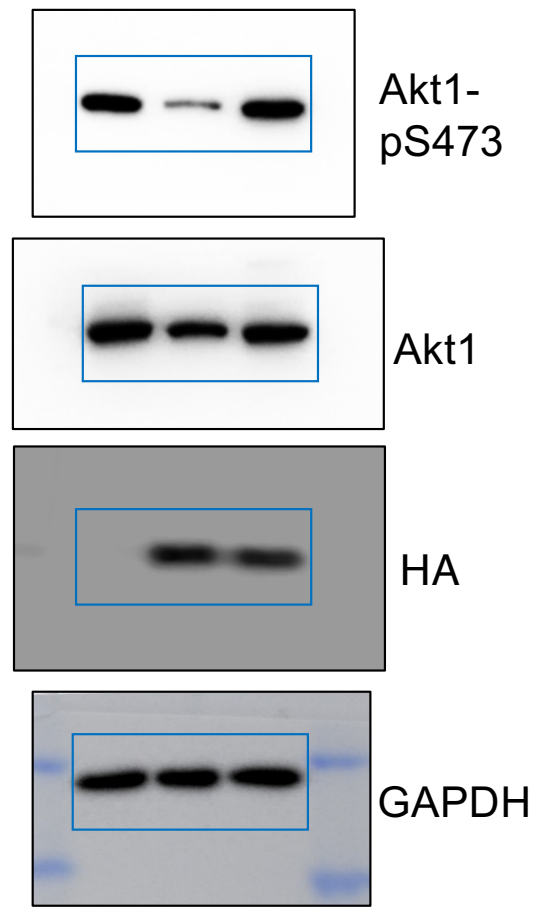

J

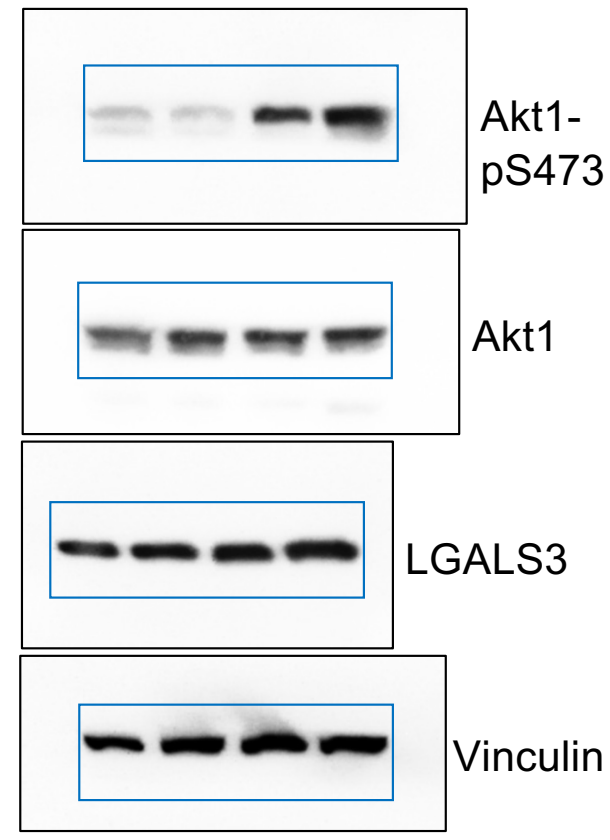

K

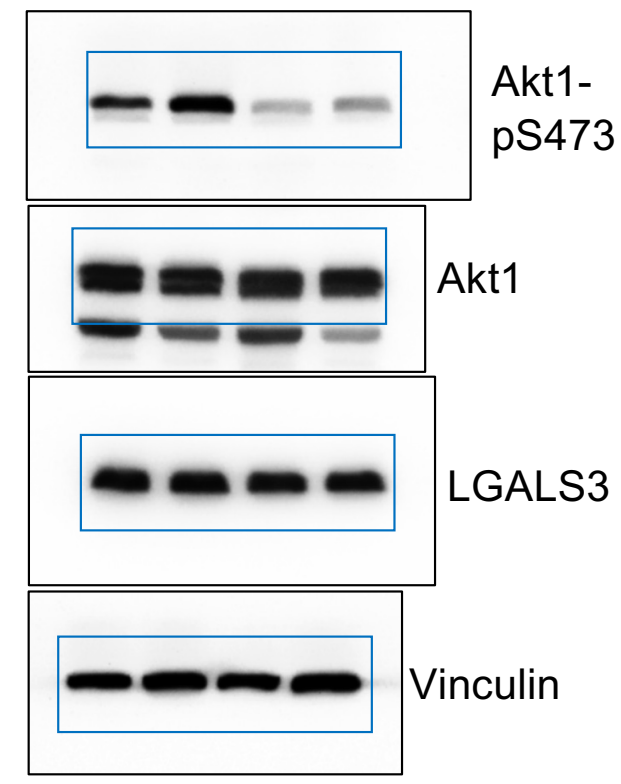

Full unedited blot for Supplementary Fig. 1

C

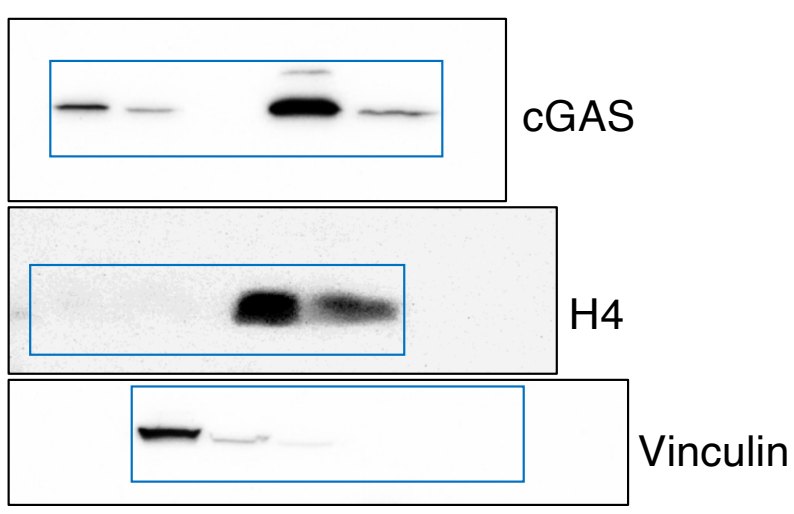

D

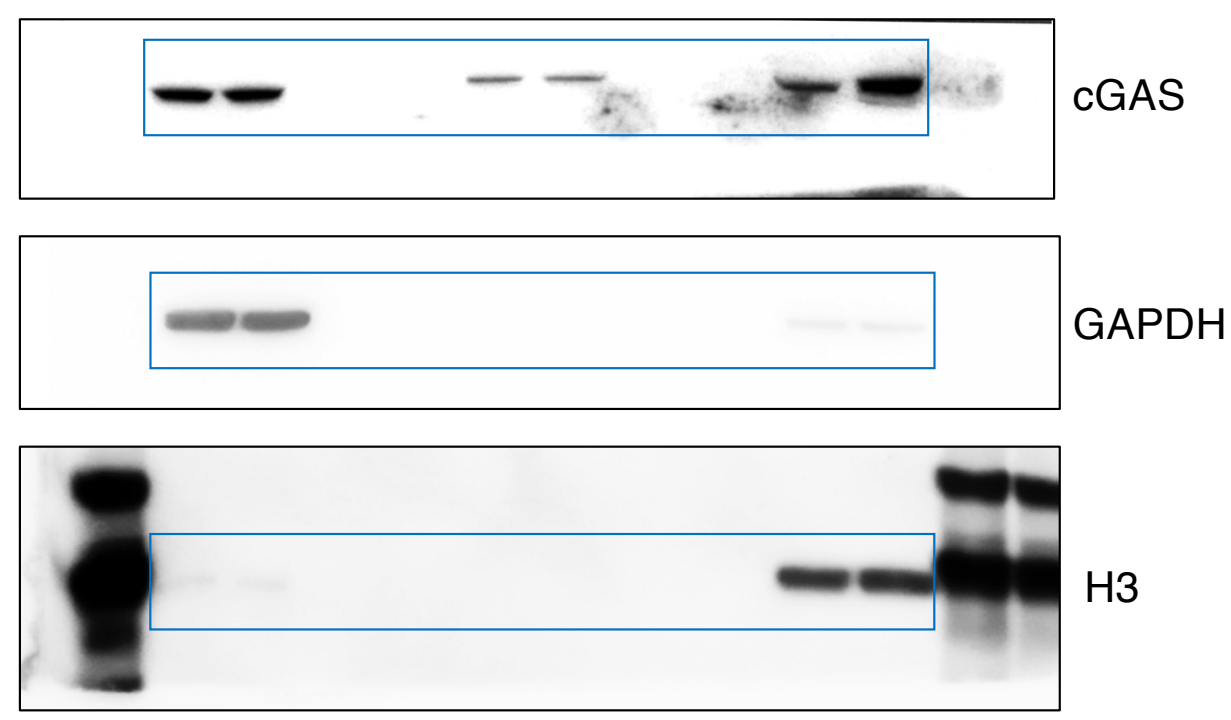

G

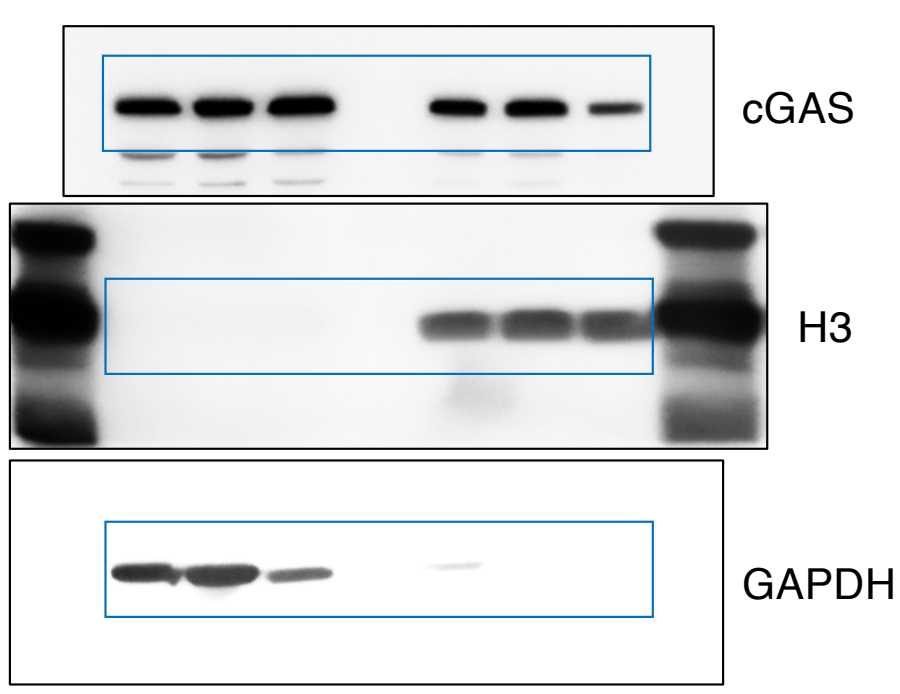

H

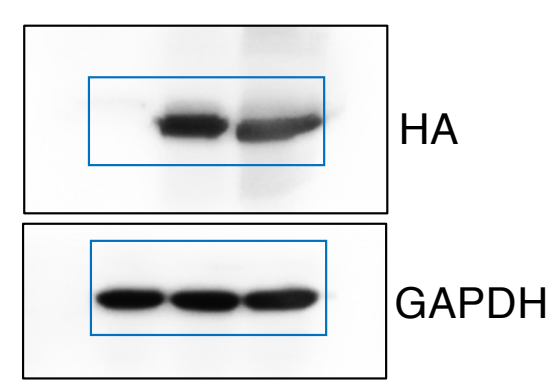

Full unedited blot for Supplementary Fig. 2

I

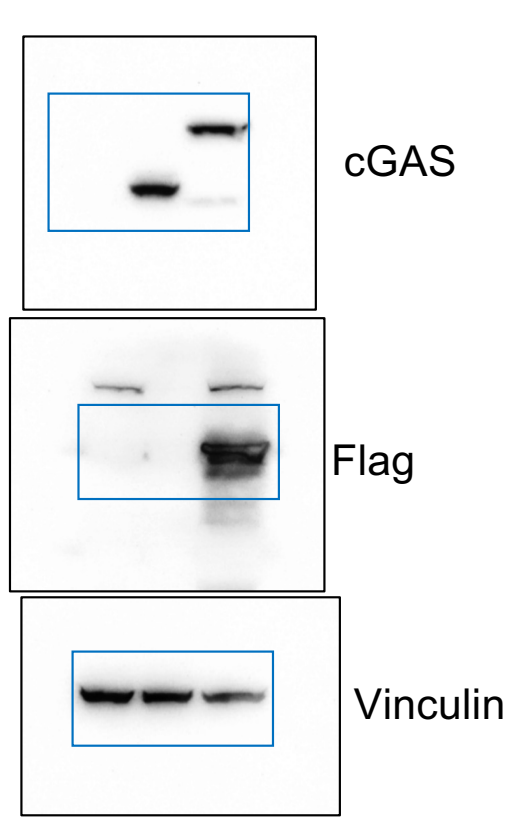

K

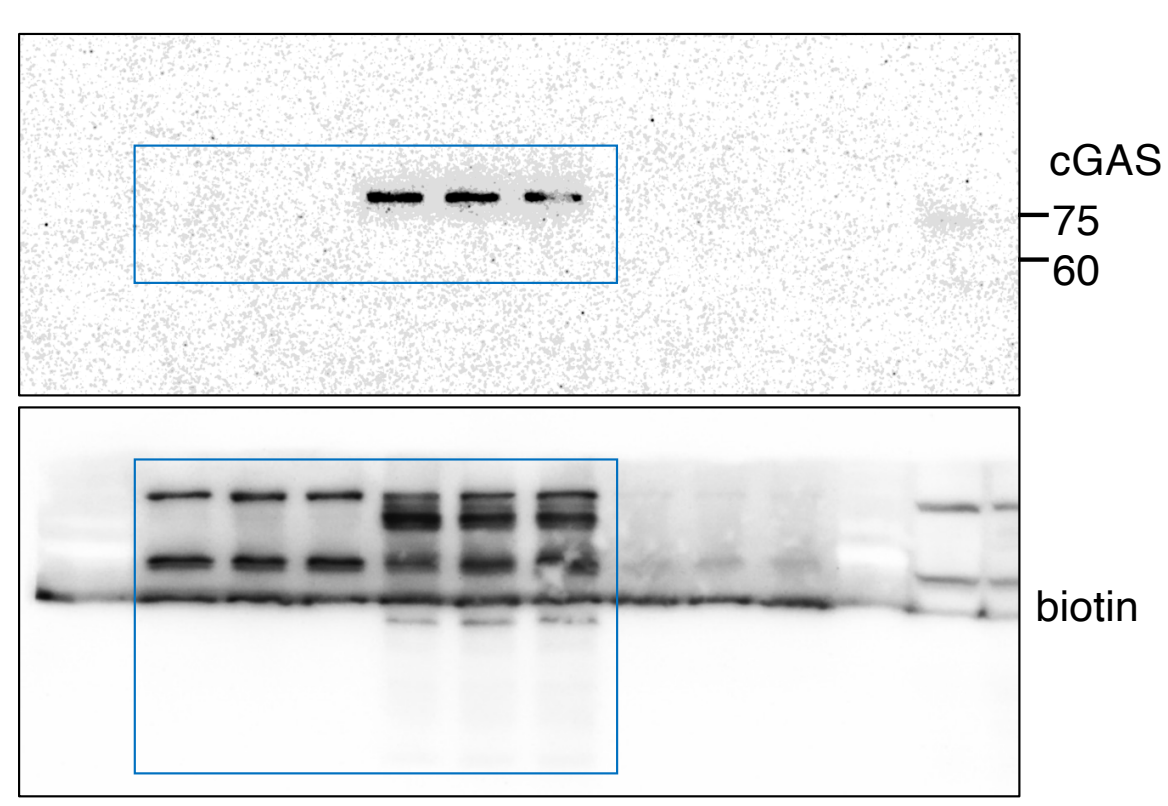

M

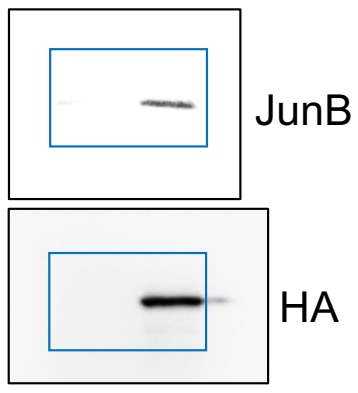

N

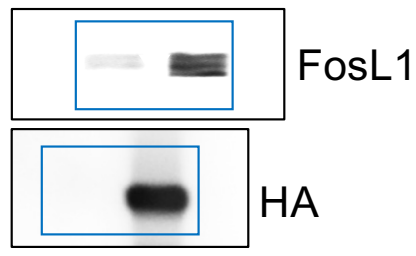

O

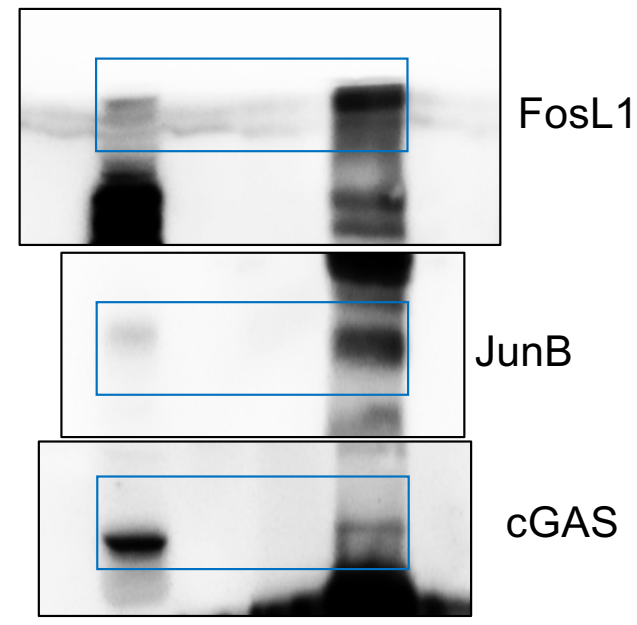

P

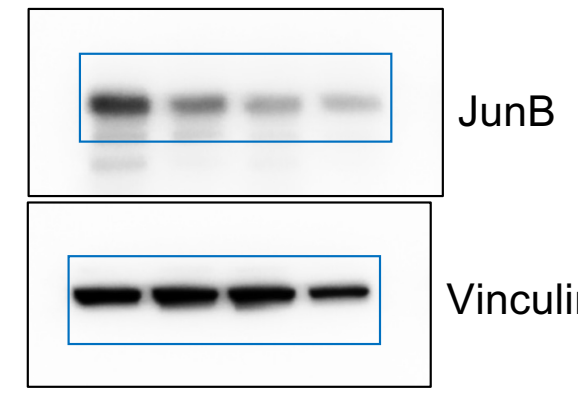

Q

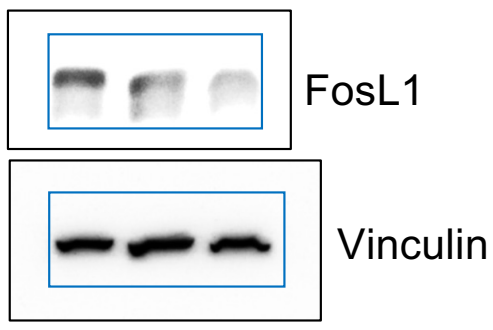

R

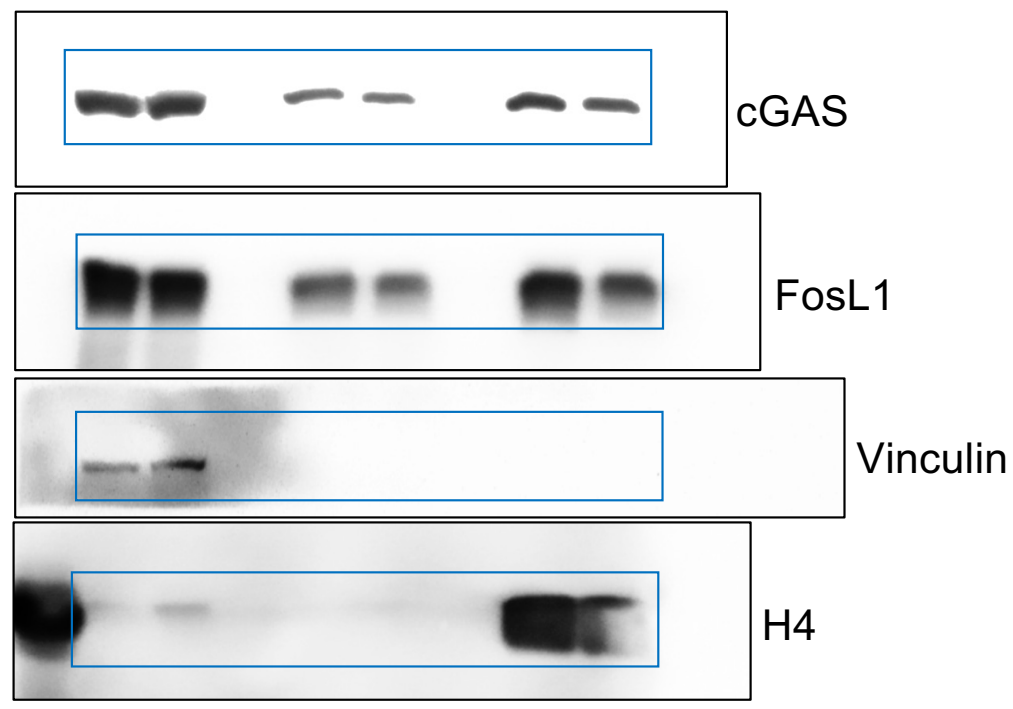

S

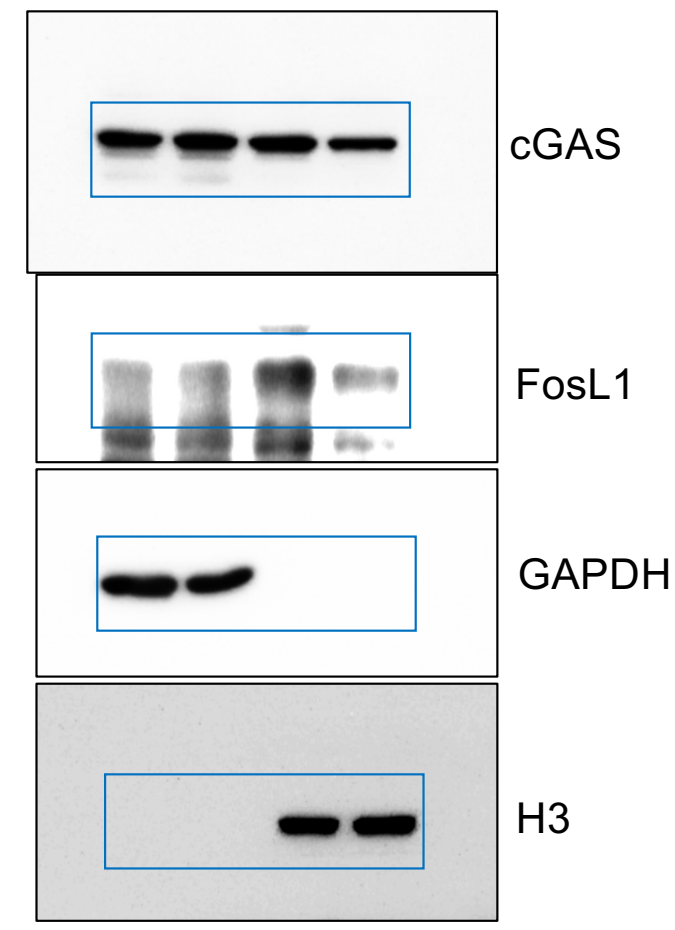

U

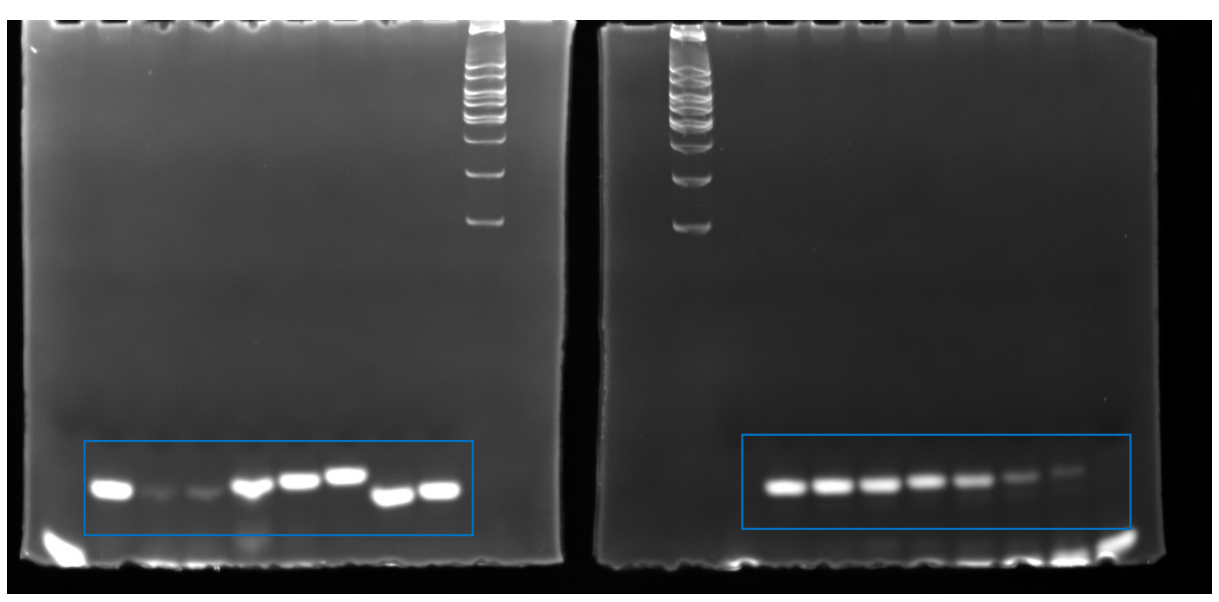

Full unedited blot for Supplementary Fig. 3

G

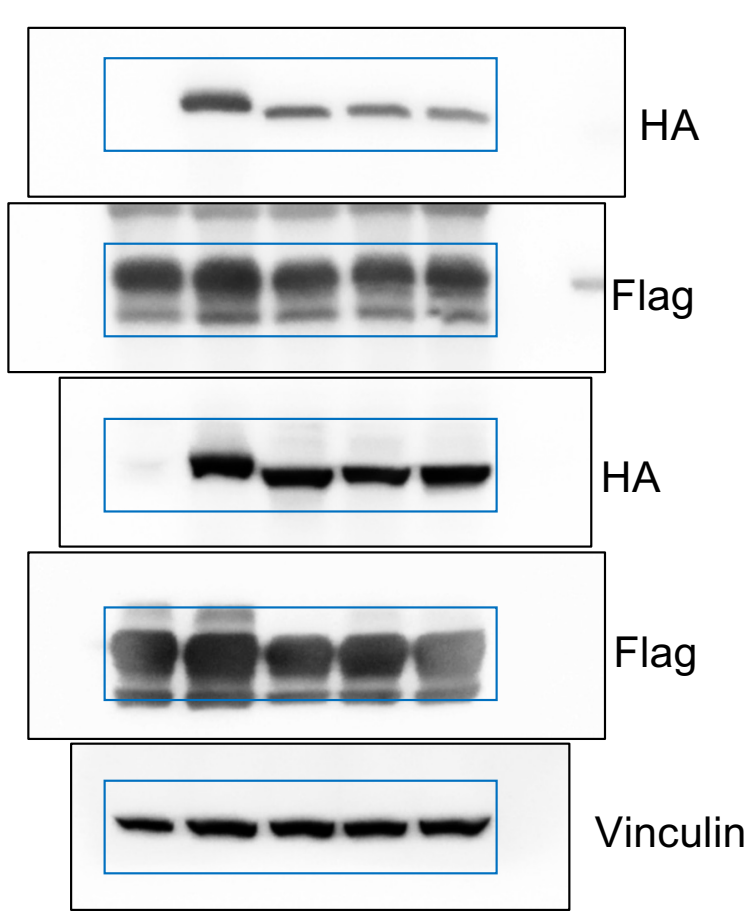

H

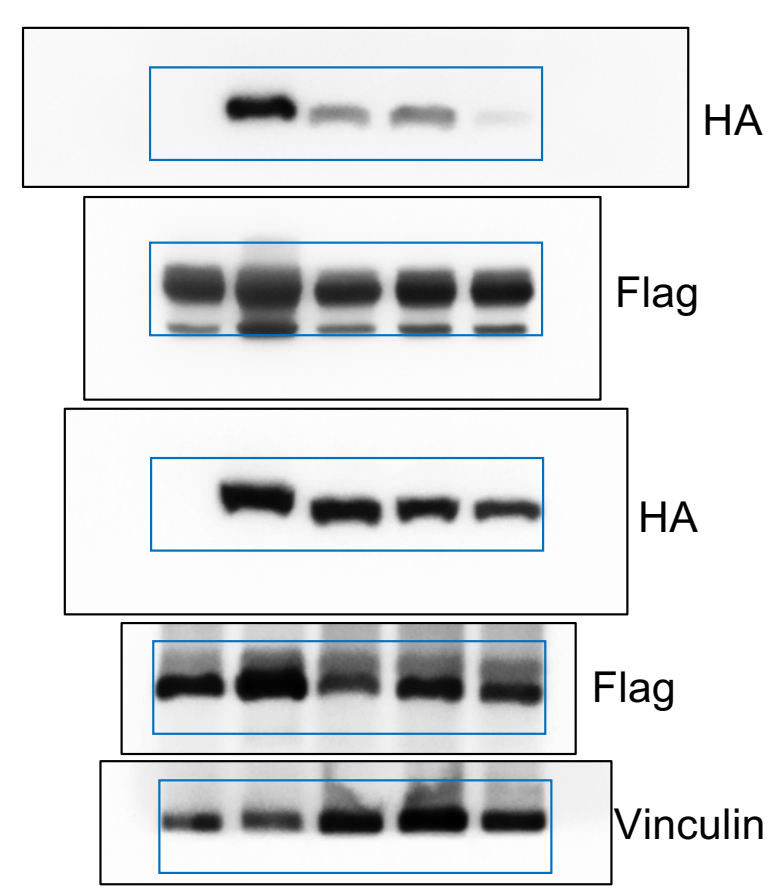

J

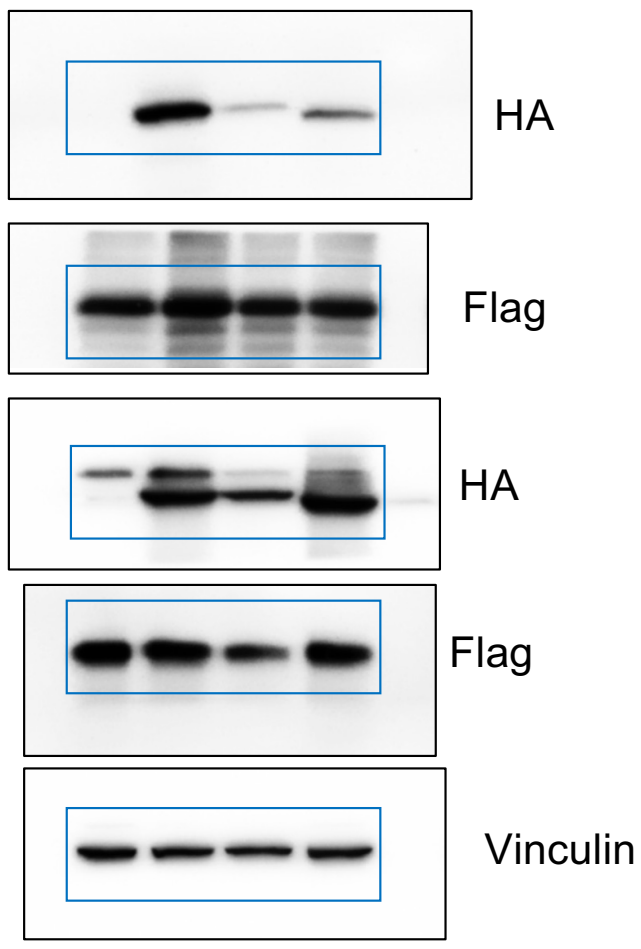

K

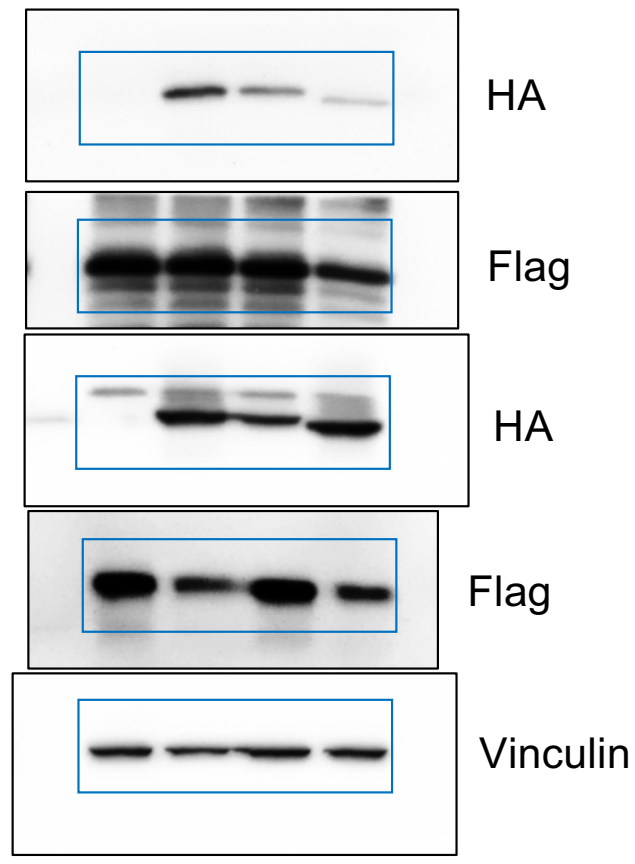

L

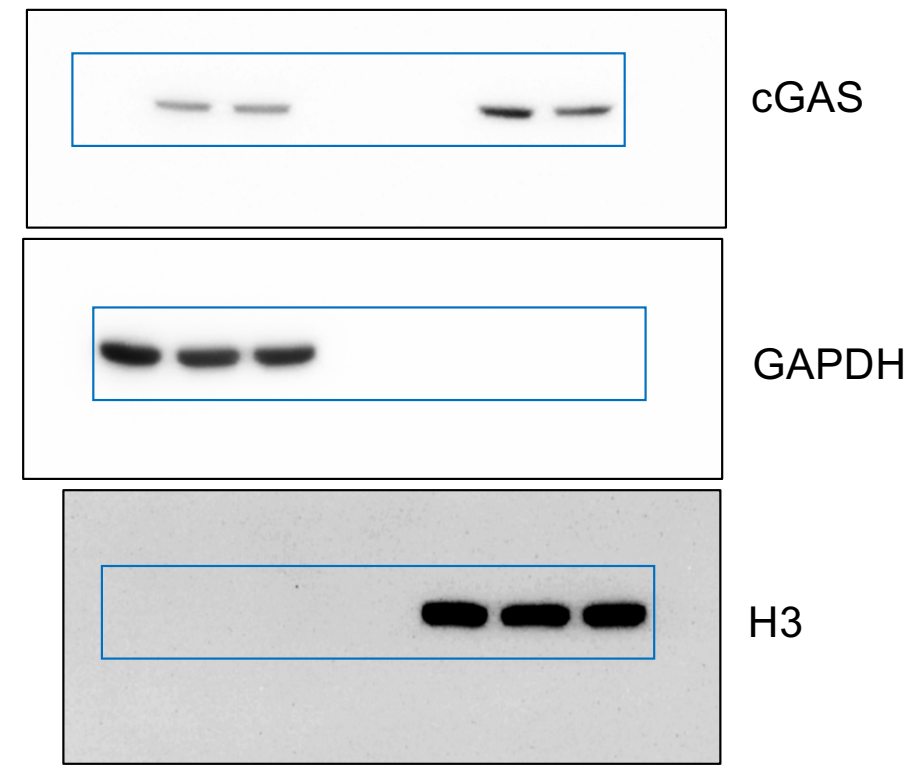

M

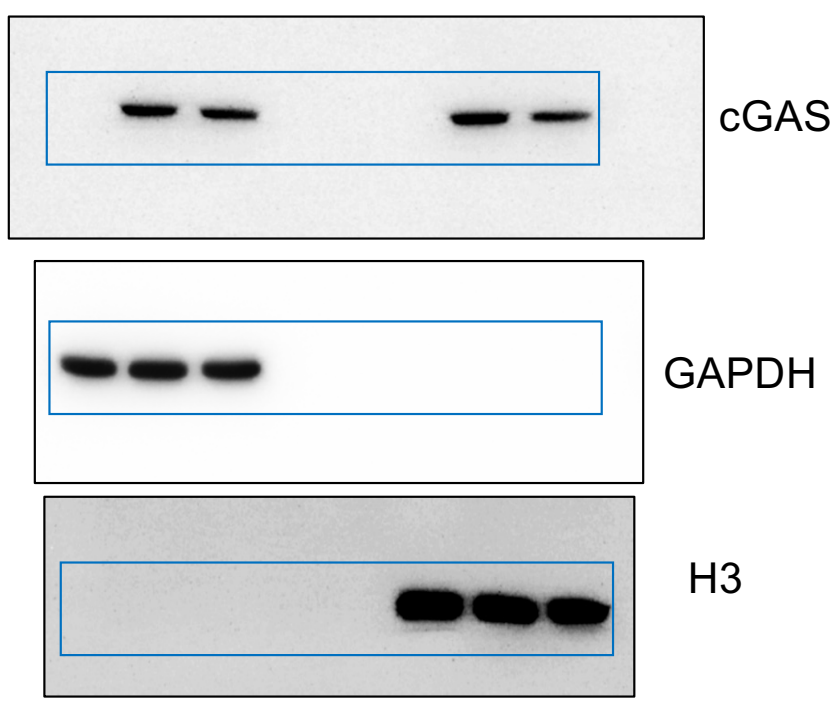

N

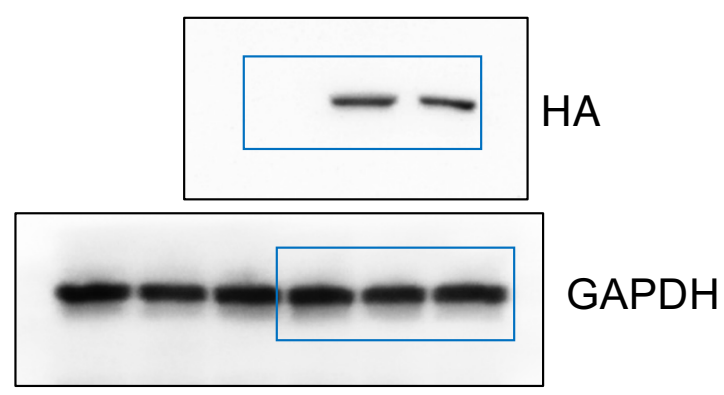

O

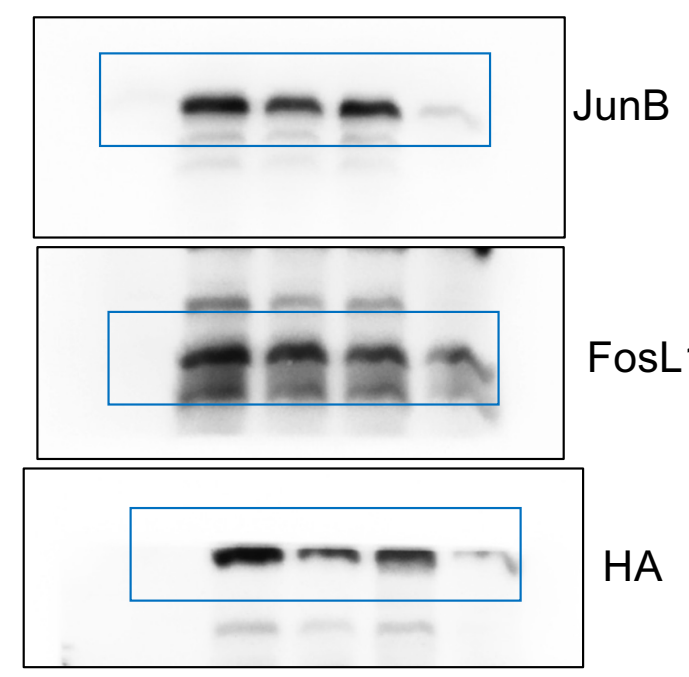

Full unedited blot for Supplementary Fig. 5

K

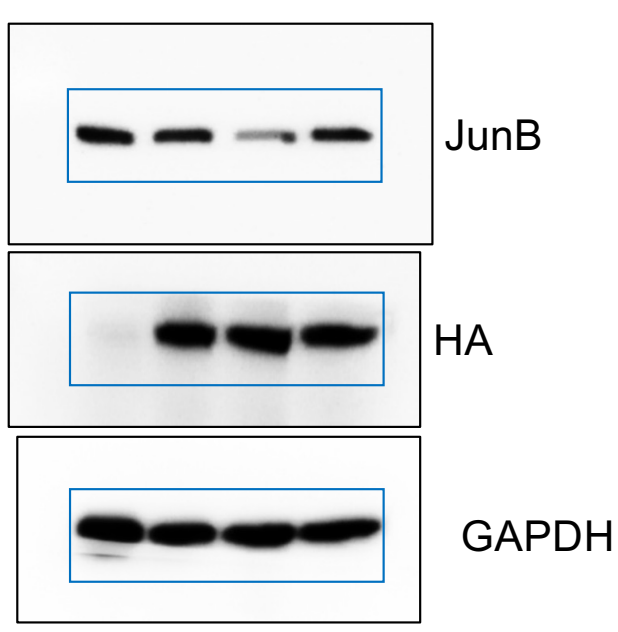

Full unedited blot for Supplementary Fig. 6

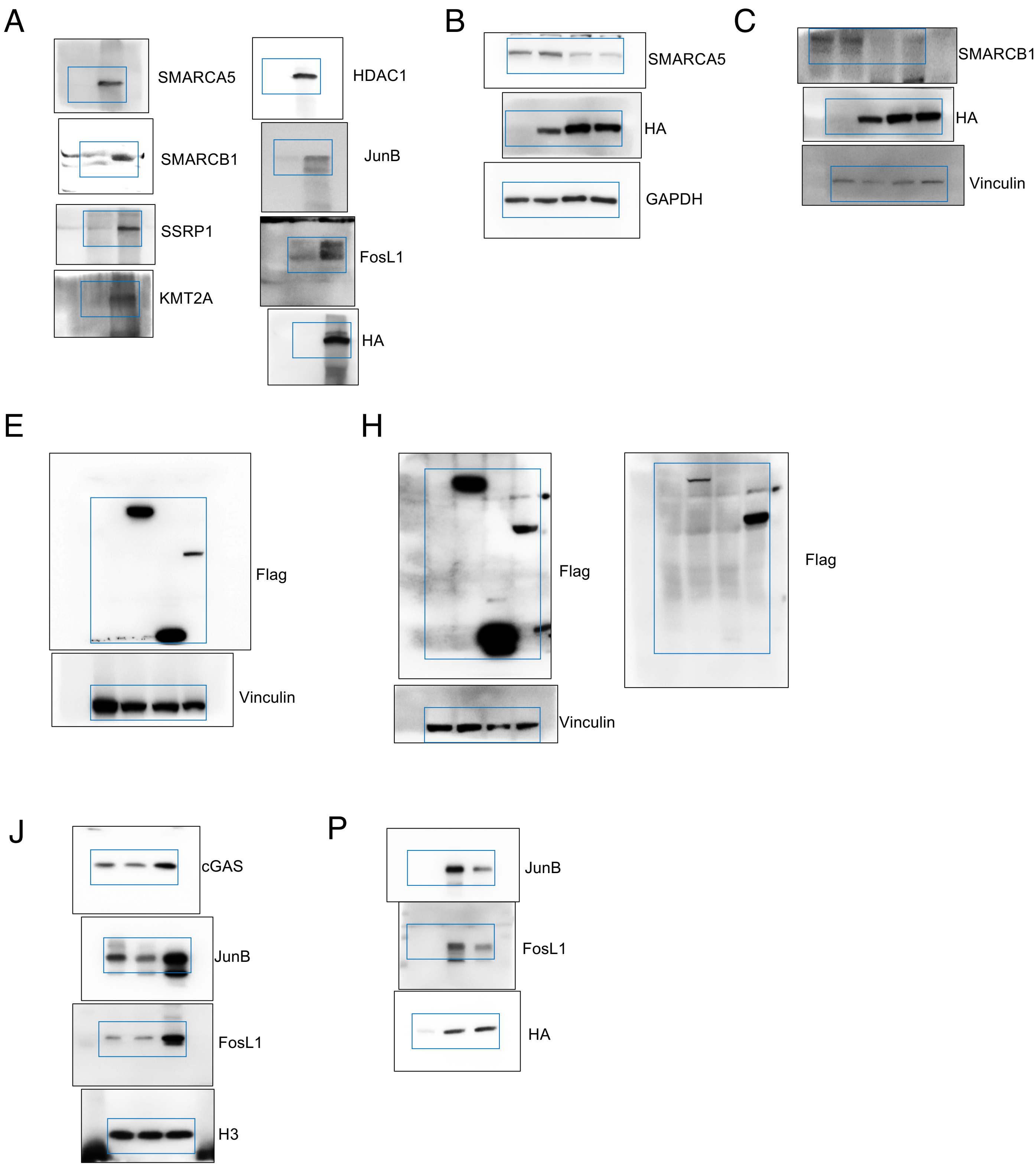

Full unedited blot for Supplementary Fig. 7

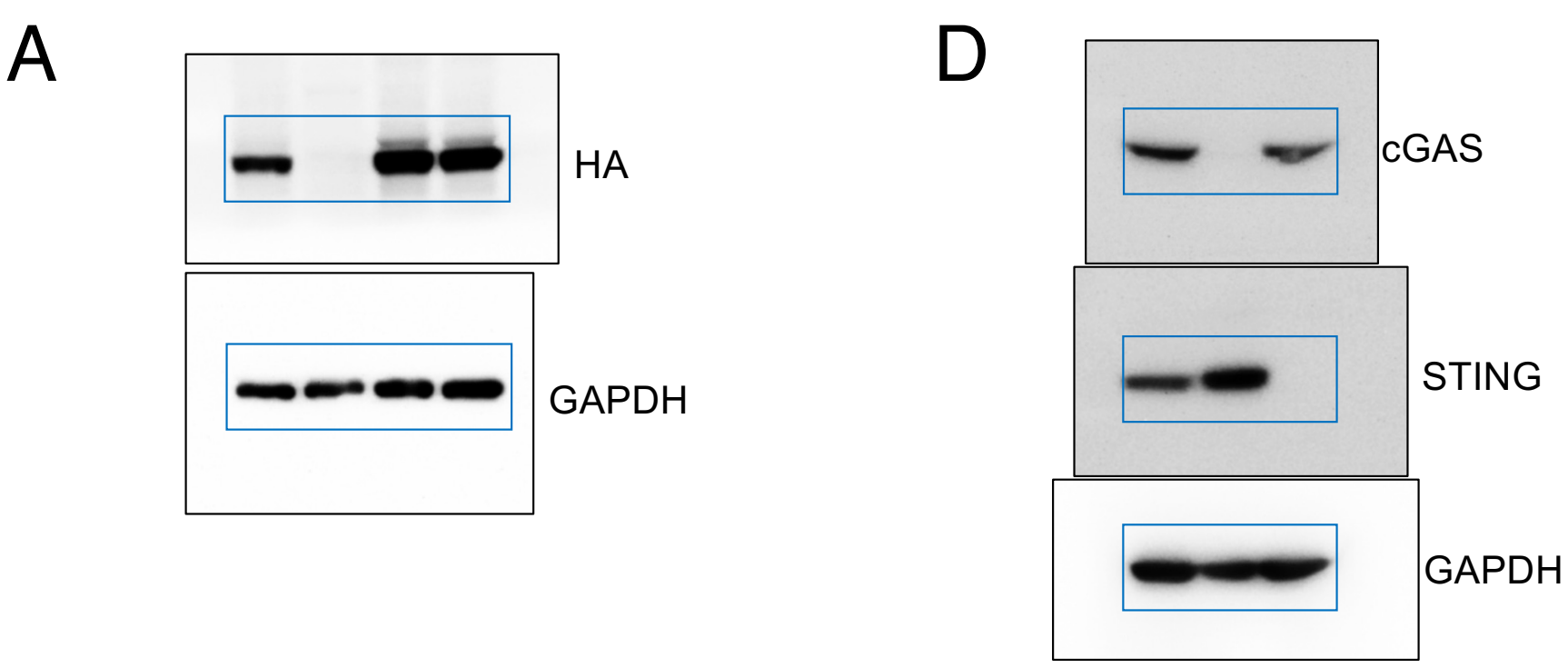

Full unedited blot for Supplementary Fig. 8

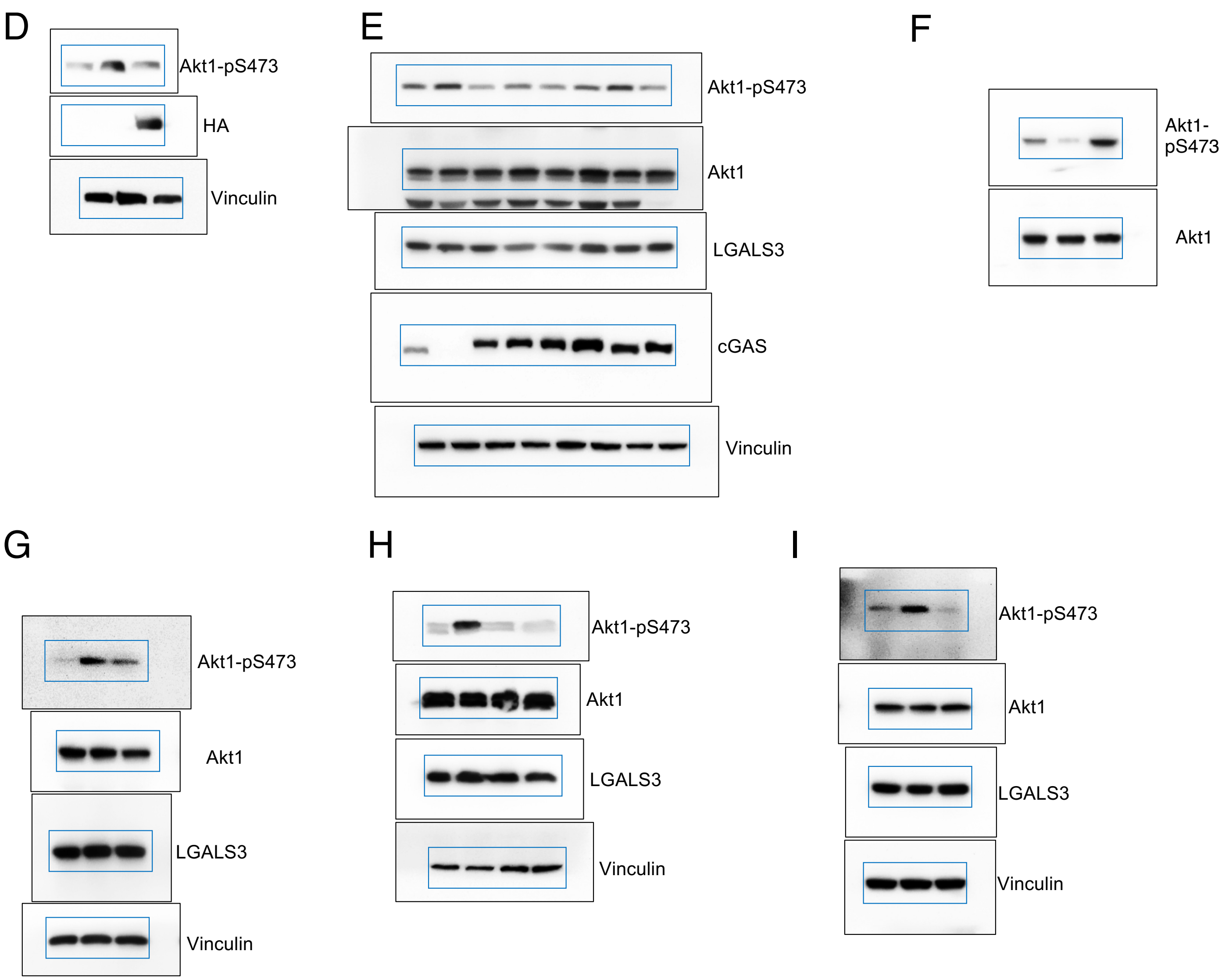

Full unedited blot for Supplementary Fig. 11

A

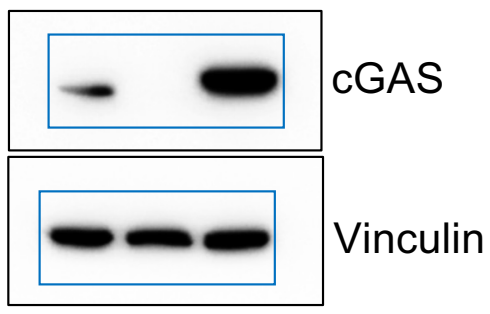

B

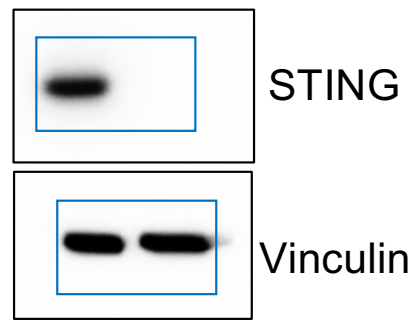

C

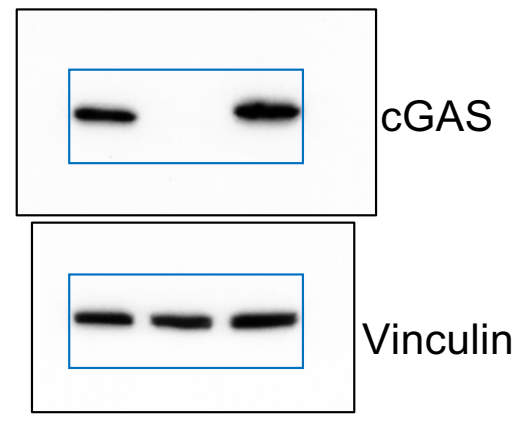

D

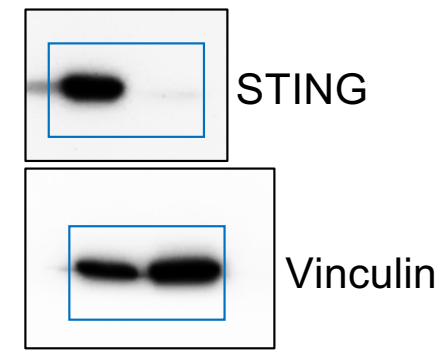

E

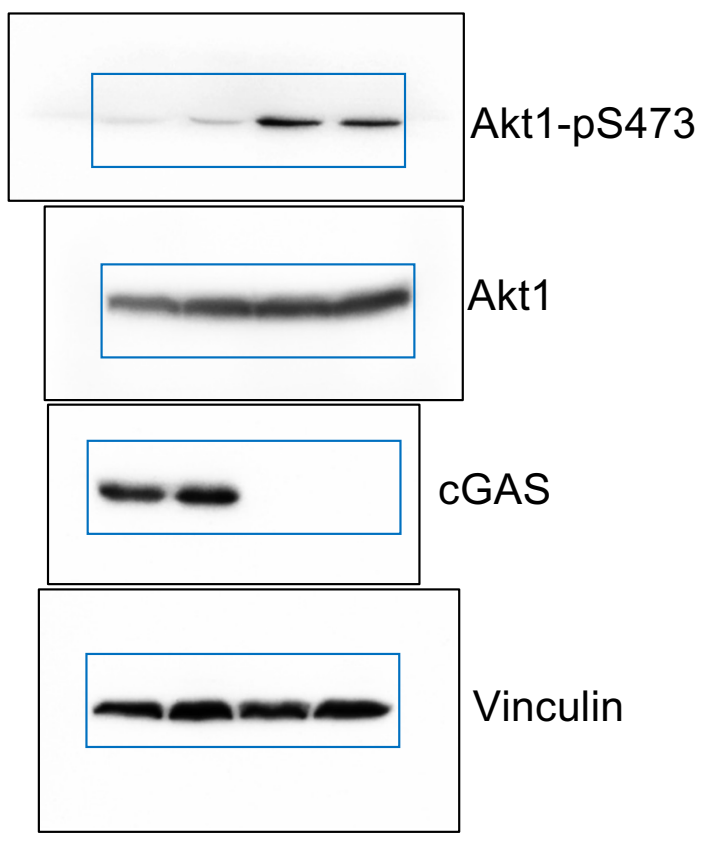

Supplement: Supplementary file 2 [file jci-136-205579-s016.pdf]
